# Optical Properties in Mid-Infrared Range of Silicon Oxide Thin Films with Different Stoichiometries

**DOI:** 10.3390/nano13202749

**Published:** 2023-10-12

**Authors:** Natalia Herguedas, Enrique Carretero

**Affiliations:** Departamento de Física Aplicada, Universidad de Zaragoza, C/Pedro Cerbuna, 12, 50009 Zaragoza, Spain; nherguedas@unizar.es

**Keywords:** thin film, optical properties, mid-infrared, silicon oxide, refractive index

## Abstract

SiO_x_ thin films were prepared using magnetron sputtering with different O_2_ flow rates on a silicon substrate. The samples were characterized using Fourier transform infrared spectroscopy in transmission and reflection, covering a spectral range of 5 to 25 μm. By employing a multilayer model, the values of the complex refractive index that best fit the experimental transmission and reflection results were optimized using the Brendel–Bormann oscillator model. The results demonstrate the significance of selecting an appropriate range of O_2_ flow rates to modify the SiO_x_ stoichiometry, as well as how the refractive index values can be altered between those of Si and SiO_2_ in the mid-infrared range.

## 1. Introduction

The mid-infrared spectral region is an area of great scientific and technological interest, but in many cases, it has not been explored as extensively as the visible or near-infrared regions. Presently, it is necessary to develop a basic understanding of the optical properties of materials in the mid-infrared range.

The deposition of thin films using magnetron sputtering enables the deposition of a material under many different conditions [1,2,3,4,5], allowing for control of the stoichiometry of composite materials [6,7,8,9,10]. This allows for the production of materials with a wide range of optical properties [11,12,13,14,15].

Thin-film SiO_2_ is a material that has been extensively studied and is widely used in numerous applications, such as materials with a low refractive index, a barrier for thermal diffusion, and for improving adhesion or enhancing mechanical strength, among many others [16,17,18,19,20,21,22,23,24]. The properties of SiO_2_ and SiO_x_ thin films have also been studied in the mid-infrared range [25,26,27], a spectral region that is of great interest in topics related to blackbody radiation at room temperature [28] or in astronomy, as well as in current research topics such as “epsilon near zero” metamaterials or the fabrication of perfect anti-reflective coatings at specific wavelengths [29,30,31,32,33,34]. In this regard, it is very interesting to have data on the refractive indices of different materials, which are often difficult to find. Therefore, it has been considered necessary to expand this knowledge by conducting a systematic study of the optical properties of thin films of SiO_x_ with different stoichiometries in order to provide these basic data to the scientific community.

The optical properties of SiO_x_ could be interesting since the properties of SiO_2_ do not always perfectly fit the requirements [35,36], for example, in the design of anti-reflective coatings with low angular dependence at a specific wavelength [31]. At this wavelength, the real part of the refractive index of a certain material equals one and the imaginary part is slightly higher than zero. This implies that, for a given material, these values of complex refractive indices occur at discrete and limited wavelengths. However, it is possible to modulate the wavelength at which this phenomenon occurs by controlling the stoichiometry of SiO_x_.

Other works, such as [37], have specifically studied the refractive indices of SiO_x_ deposited by reactive sputtering while varying the reactive gas flow. However, although the results were useful and of high quality, the study did not determine the stoichiometry of the deposited SiO_x_ under each deposition condition. Furthermore, from the results obtained in [37], it could be observed that there was little variation in the refractive indices obtained, which suggested that in all cases, the stoichiometry was close to that of SiO_2_.

In this study, we aimed to determine the refractive indices of SiO_x_ in the mid-infrared range using FTIR spectroscopy by controlling the stoichiometry while varying the O_2_ flow during deposition and determining the stoichiometry through EDS. Special attention was given to the study of slightly sub-oxidized SiO_x_, which allowed for the modulation of the optical properties of SiO_2_.

## 2. Materials and Methods

In this study, we deposited SiO_x_ thin films on a silicon substrate because it is transparent in the mid-infrared range. These silicon wafers had a thickness of 260~300 μm, 1~5 Ω/cm of resistance, and were orientated to <100> ± 1 deg.

These SiO_x_ thin films were obtained using a prototype industrial magnetron sputtering installation, comprising three vacuum chambers. The loading chamber and the transfer chamber, which were smaller in size, facilitated rapid sample introduction and extraction without breaking the vacuum in the process chamber where the sputtering process took place. The achievable pressures in these chambers were in the order of 10^−2^, 10^−7^, and 10^−7^ mbar for the loading, transfer, and process chambers, respectively.

The installation included 8 distinct magnetrons, 4 on each side, enabling the deposition of up to 4 different materials on each sample. The targets used had dimensions of 600 × 100 mm. The silicon target used had a purity of 99.999%.

Additionally, two pulsed DC power supplies were available, capable of delivering up to 10 kW of power. For this study, a power of 2 kW was utilized. Gas flows were regulated using mass flow controllers (MFCs). The substrate holder linearly moved at a constant velocity, ensuring that each point at the same height received the same exposure during the deposition process. Furthermore, points within the same vertical line had equal exposure due to the larger dimensions of the magnetrons compared with the sample.

Once the SiO_x_ thin film was deposited, its thickness was measured using a profilometer (DektakXT^®^ model), which had a precision in the order of nanometers.

Transmission and reflection spectra were measured with an FTIR spectrophotometer, specifically the Perkin-Elmer Spectrum 100 model, which operates in the infrared range from 4000 to 200 cm^−1^ (2.5 to 50 µm) with a step size of 2 cm^−1^ and a resolution of 16 cm^−1^, which is the maximum resolution allowed by the spectrophotometer and was chosen in order to avoid interference from the substrate. Measurements were conducted in the air, ensuring that they were repeatable and unaffected by atmospheric effects.

Samples were also analyzed using a Field Emission Scanning Electron Microscope (FESEM) Carl Zeiss MERLIN™ in order to obtain images and determine the proportion of the Si and O in the SiO_x_ thin films. This SEM equipment allowed for observations with a spatial resolution of up to 0.8 nm and acceleration voltages ranging from 0.02 to 30 kV. It was equipped with an EDS detector for energy analysis of the scattered X-rays, specifically an X-Max (20 mm^2^) detector with a Silicon Drift Detector (SSD) from Oxford Instruments. The EDS detector provided an energy resolution below 123 eV at the 5.9 keV Mn Ka.

## 3. Theoretical Background and Calculation

In this study, we experimentally determined the energetic factors of several SiO_x_ thin films, including transmission factors, coating reflectance, and substrate reflectance. Our objective was to extract the complex refractive index, $\tilde{n}=n+i\kappa$, which characterizes these materials.

First, the transmission, coating reflection, and substrate reflection spectra were measured using an FTIR spectrophotometer in the infrared range. To establish a relationship between these experimental energetic factors and the refractive index, a matrix formalism was employed, which was based on the foundations laid out in [38,39]. This formalism was derived from Maxwell’s equations while ensuring the continuity of the transverse components of the fields. To achieve this, the materials were assumed to be non-magnetic, linear, homogeneous, isotropic, without free charge density, and infinite in the plane of the layers.

**Figure 1:**
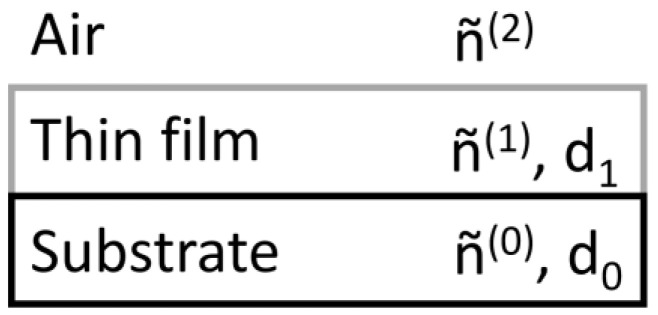
Schematic representation of the indices used for the mathematical model.

In our model, we considered a thin film (indexed as one, as shown in Figure 1), a substrate material of silicon (indexed as zero), and the incident medium of air (indexed as two). Each material, denoted as i, is characterized by its complex refractive index, ${\tilde{n}}^{\left(i\right)}$, and its corresponding thickness, $d_{i}$. The wave impinged on the thin film from the medium indexed as two. The analysis was divided into two parts: first, the reflection and transmission through the thin film without crossing the substrate, and then the subsequent consideration of the substrate’s effect.

For the analysis of the thin film, it was assumed that media zero and two were infinite. Under normal incidence measurements, the transverse electric and magnetic components were equal. When transitioning from medium j to medium i, the relationship between the wave amplitudes is given by:

(1) $$A^{\left(i\right)}=T^{\left(i,j\right)}\cdot A^{\left(j\right)}\;,$$

where $T^{\left(i,j\right)}$ is the transmission matrix at the interface between medium j and medium i:

(2) $$T^{\left(i,j\right)}=\frac{1}{2}\left(\begin{array}{cc} 1+\frac{{\tilde{n}}^{\left(j\right)}}{{\tilde{n}}^{\left(i\right)}} & 1-\frac{{\tilde{n}}^{\left(j\right)}}{{\tilde{n}}^{\left(i\right)}}\\ 1-\frac{{\tilde{n}}^{\left(j\right)}}{{\tilde{n}}^{\left(i\right)}} & 1+\frac{{\tilde{n}}^{\left(j\right)}}{{\tilde{n}}^{\left(i\right)}} \end{array}\right)\;.$$

Furthermore, as the wave propagated through a medium, its amplitude underwent a change, which is given by:

(3) $$A^{\left(i\right)}\left(x\right)=P^{\left(i\right)}\left(x\right)\cdot A^{\left(j\right)}\left(0\right)\;,$$

where $A^{\left(i\right)}\left(x\right)$ represents the amplitudes after propagating a distance x in medium i, and $P^{\left(i\right)}\left(x\right)$ is the propagation matrix in medium i, which has the following expression:

(4) $$P^{\left(i\right)}\left(x\right)=\frac{1}{2}\left(\begin{array}{cc} e^{jk{\tilde{n}}^{\left(i\right)}x} & 0\\ 0 & e^{-jk{\tilde{n}}^{\left(i\right)}x} \end{array}\right)\;.$$

where j is the imaginary unit and k is the wave number in a vacuum.

Using Equations (2) and (4), the amplitude in medium zero could be calculated from the amplitude in medium two as:

(5) $$A^{\left(0\right)}=T^{\left(0,1\right)}\cdot P^{\left(1\right)}\left(d_{1}\right)\cdot T^{\left(1,2\right)}=M\cdot A^{\left(2\right)}\;.$$

From this M matrix, the transmission (τ) and reflection (ρ) intensities in the thin film can be obtained as:

(6) $$\tau =\frac{{\tilde{n}}^{\left(0\right)}}{{\tilde{n}}^{\left(2\right)}}{\left|M_{11}-\frac{M_{12}M_{21}}{M_{22}}\right|}^{2}\;,$$

(7) $$\rho ={\left|\frac{M_{21}}{M_{22}}\right|}^{2}\;.$$

When studying the effect of substrate thickness, it is important to consider that the interferences occurring with the substrate have a much higher frequency than the spectral resolution of the spectrophotometer that we used. As a result, what was measured with the spectrophotometer was the incoherent sum of the intensities from multiple reflections at the interfaces. However, the resolution was enough to observe the monolayer interference.

Let $\tau_{c}$ represent the transmission of the coating, $\rho_{c}$ the reflection of the thin film if incident from air, ${\rho_{c}}^{\prime }$ the reflection of the thin film when incident from the substrate, $\rho_{v}$ the reflection at the substrate–air interface, $d_{0}$ the thickness of the substrate, and α the absorption of the substrate, which can be related to the imaginary part of its refractive index, κ, as:

(8) $$\alpha =\frac{4\pi \kappa }{\lambda }.$$

Then, the total transmission through the thin film and the substrate is given by:

(9) $$T\left(\lambda \right)=\frac{\left(1-\rho_{v}\right)\tau_{v}e^{-\alpha d}}{1-\rho_{c}^{\prime }\rho_{v}e^{-2\alpha d}}\;,$$

and the reflection on the side of the thin film is:

(10) $$R_{multilayer}\left(\lambda \right)=\rho_{c}+\frac{\rho_{v}\tau_{c}^{2}e^{-2\alpha d}}{1-\rho_{c}^{\prime }\rho_{v}e^{-2\alpha d}}\;,$$

and on the substrate side:

(11) $$R_{substrate}\left(\lambda \right)=\rho_{v}+\frac{{\left(1-\rho_{v}\right)}^{2}\rho_{c}^{\prime }e^{-2\alpha d}}{1-\rho_{c}^{\prime }\rho_{v}e^{-2\alpha d}}\;.$$

T(λ), $R_{multilayer}(\lambda )$, and $R_{substrate}(\lambda )$ are the experimental data that we obtained from the spectrophotometer. The substrate width, specified by the silicon manufacturer, was $d_{0}=3$00 μm, while the width of the SiO_x_ thin film was measured using a profilometer.

First, the refractive index of silicon was calculated. For this purpose, the same mathematical approach was used, but with the consideration that $d_{1}=0$, indicating the absence of a thin film. The spectra of the substrate were measured, and various values of n and κ were considered. The values that best fit the experimental spectra were selected. Subsequently, the same method was applied to the thin films.

The results obtained for n and κ using this method were quite noisy, so we adjusted them to a model. There were various mathematical models available to approximate n and κ. In this study, we used the Brendel–Bormann oscillator model [40], which constructs a dielectric function that satisfies the Kramers–Kronig relations and yields with a Gaussian shape for the imaginary part. The dielectric constant is given by the addition of different vibrational modes:

(12) $$\varepsilon =\varepsilon_{\infty }+\sum_{j=1}^{m}X_{j}(\nu )\;,$$

where ν is the wavenumber, i.e., the inverse of the wavelength (ν=1/λ); $\varepsilon_{\infty }$ is the high-frequency permittivity; and j refers to the different vibrational modes and the contribution of each one, $X_{j}(\nu )$, is defined by:

(13) $$X_{j}\left(\nu \right)=\frac{1}{\sqrt{2\pi }\sigma_{j}}\int_{-\infty }^{\infty }\exp\left(\frac{{\left(x-\nu_{oj}\right)}^{2}}{2\sigma_{j}^{2}}\right)\frac{\nu_{pj}^{2}}{x^{2}-\nu^{2}-i\nu_{\tau j}\nu }dx\;,$$

where $\sigma_{j}$ is the standard deviation of the Gaussian distribution, $\nu_{oj}$ is the center frequency, $\nu_{pj}$ is the plasma frequency (which gives the oscillator strength), and $\nu_{\tau j}$ is the damping constant.

As shown in Appendix A of [41], Equation (13) could be analytically solved and the result was:

(14) $$X_{j}\left(\nu \right)=\frac{i\sqrt{\pi }\nu_{pj}^{2}}{2\sqrt{2}\sigma_{j}a_{j}(\nu )}\left[\omega \left(\frac{a_{j}\left(\nu \right)-\nu_{oj}}{\sqrt{2}\sigma_{j}}\right)+\omega \left(\frac{a_{j}\left(\nu \right)+\nu_{oj}}{\sqrt{2}\sigma_{j}}\right)\right]\;,$$

where $a_{j}\left(\nu \right)=\sqrt{\nu^{2}+i\nu_{\tau j}\nu }$ must be chosen in such a way that $I\left(a_{j}\right)>0$, i.e.,

(15) $$R\left(a_{j}\left(\nu \right)\right)=\nu \sqrt{\frac{\sqrt{1+{\left(\nu_{\tau j}/\nu \right)}^{2}}+1}{2}}\;,$$

(16) $$I\left(a_{j}\left(\nu \right)\right)=\nu \sqrt{\frac{\sqrt{1+{\left(\nu_{\tau j}/\nu \right)}^{2}}+1}{2}}\;,$$

where R and I refer to the real and imaginary parts, respectively, and $\omega \left(z\right)$ is the Faddeeva function, defined by:

(17) $$\omega \left(z\right)=e^{-z^{2}}erfc\left(-iz\right)=e^{-z^{2}}\left(1+\frac{2i}{\sqrt{\pi }}\int_{0}^{z}e^{t^{2}}dt\right)\;.$$

The complex refractive index, $\tilde{n}$, is related to the dielectric function by $\varepsilon ={\tilde{n}}^{2}={\left(n+i\kappa \right)}^{2}$, so that it is possible to obtain n and κ from Equation (14), resulting in the following expressions:

(18) $$n=\frac{R\left(\varepsilon \right)\pm \sqrt{R{\left(\varepsilon \right)}^{2}+I{\left(\varepsilon \right)}^{2}}}{2}\;,$$

(19) $$\kappa =\frac{I\left(\varepsilon \right)}{n}\;.$$

Given these equations, we searched for the oscillator parameters that best fit the previously adjusted values of n and κ. The number of oscillators considered was determined depending on the number of peaks in the κ spectrum.

## 4. Results and Discussion

**Figure 2:**
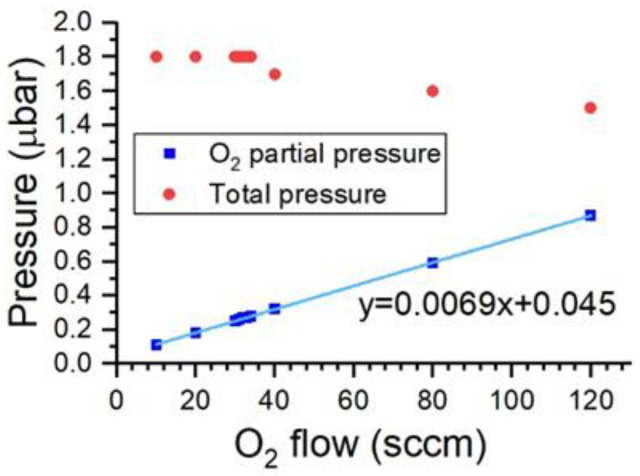
Relationship between pressure and the flow of gas introduced.

To obtain SiO_x_ with different stoichiometries, the main parameter that needed to be controlled was the oxygen flow that was introduced into the vacuum chamber during the deposition process. All the coatings were deposited with a total flow of 200 sccm (sum of the Ar and O_2_ flows). As shown in Figure 2, the partial pressure of oxygen linearly increased with the O_2_ flow that was introduced by a mass flow controller (MFC). A linear fit of this trend is shown in Figure 2 along with the equation of the least-squares fit line. Moreover, in Figure 2, the total pressure of the chamber is shown as a function of the O_2_ flow. In this case, it can be observed that the pressure remained relatively constant, with a slight decrease observed for high values of the O_2_ flow.

**Figure 3:**
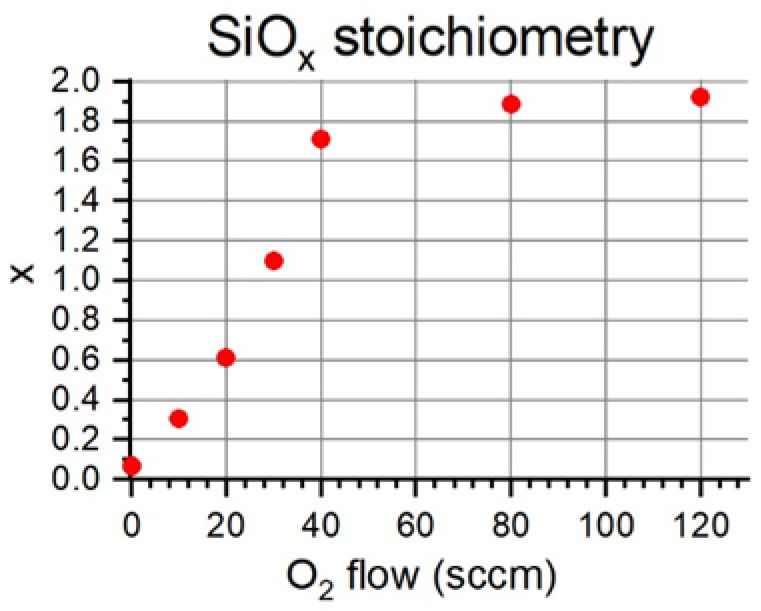
SiO_x_ stoichiometry as a function of the O_2_ flow introduced during the deposition process.

Figure 3 shows the evolution of the stoichiometry of the SiO_x_ samples as a function of the O_2_ flow with which they were deposited. It can be seen that for a flow of 0 sccm of O_2_, x = 0.06 was obtained, a value very close to zero. The value of 0.06 took into account possible contamination when handling the samples, being very likely that they had been slightly oxidized in their exposure to the atmosphere. Between the flows of 0 and 20 sccm of O_2_, x approximately linearly grew, reaching a value of x = 0.61 for 20 sccm of O_2_. For values between 20 and 40 sccm of O_2_, the trend had a higher slope, reaching a value of x = 1.71 for a flow of 40 sccm of O_2_. This increase in slope was due to the entry into the reactive mode of the deposition process and its consequent decrease in deposition rate. The decrease in deposition rate entailed a higher degree of oxidation of the evaporated material. For flow values greater than 40 sccm, the value of x tended “asymptotically” toward the expected value for the stoichiometry of SiO_2_ of x = 2, reaching a value of x = 1.92 for a flow of 120 sccm of O_2_. Additionally, X-ray diffraction confirmed the amorphous nature of the SiO_x_ samples, while Figure S1 in the Supplementary Material shows cross-sectional images of the thin films obtained using field emission scanning electron microscopy.

The fitting parameters for the dielectric permittivity value of each sample were determined using the Brendel–Bormann oscillator model. The obtained results are presented in Table 1 for the samples deposited with different O_2_ flows ranging from 10 to 120 sccm. The number of absorption peaks included in the model varied from four to six depending on the sample. This number was chosen in such a way that the model corresponded with the data, identifying the principal absorbance bands, which corresponded with those found in the literature [42,43,44]. This showed that resonances at approximately 440 and 810 cm^−1^ corresponded to Si-O-Si rocking and bonding bands, respectively, while the bands at 1060 and 1150 cm^−1^ corresponded to Si-O-Si stretching bands.

**Figure 4:**
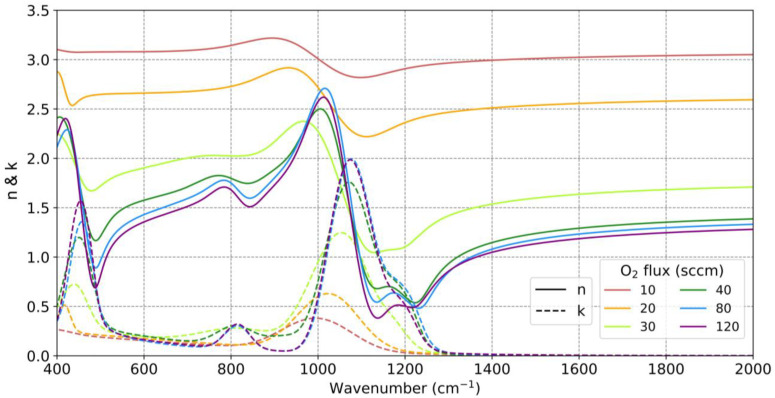
Complex refractive indices for SiO_x_ samples deposited with different reactive gas flows: real part n (solid line) and imaginary part k (dotted line).

Using the values from these fittings, we can calculate the complex refractive indices of the different SiO_x_ samples, as shown in Figure 4. It can be observed that the real part of the refractive index, n, decreased as the O_2_ flow increased. While pure Si had a refractive index of approximately 3.42, the sample deposited with 10 sccm of O_2_ exhibited an n value of around three for all wavelengths, accompanied by a weak absorption band around 1000 cm^−1^. The real part of the refractive index continued to decrease until reaching an approximate value of n = 2.5 for the sample deposited at 20 sccm. On the other hand, the absorption in the approximately 1000 cm^−1^ band increased, and this absorption band shifted to higher wave numbers (shorter wavelengths) as the O_2_ flow increased. In [45], this effect was explained by O back-bonding or a variation of the Si–O–Si bond angles. In [46], researchers showed that the absorption strength in each band linearly scaled with the oxygen concentration and that this vibration was specifically determined by the detailed character of the bonding geometry. A further explanation about geometry and atomic displacements can be found in [44,46].

For an O_2_ flow of 30 sccm (in which a value of x = 1.1 was obtained, indicating a stoichiometry very close to silicon monoxide), the trend in the complex refractive index followed the same pattern as in lower flows. However, at an O_2_ flow of 40 sccm (in which a value of x = 1.7 was reached), the complex refractive index values obtained bore a strong resemblance to those obtained at higher O_2_ flows, although they followed a similar trend as that of the previous cases. The similarity among the refractive indices deposited with flows of 40, 80, and 120 sccm suggested that starting from 40 sccm, the deposited material already exhibited characteristics very similar to those of SiO_2_. Despite the significant change in deposition conditions due to the substantial difference in the reactive gas flow, the samples barely altered their compositions, as observed in Figure 3, and the refractive indices showed minimal changes. The slight change in refractive indices when altering the O_2_ flow for high flow rates highlighted the significance of carefully selecting an appropriate range of reactive gas flows to effectively control the stoichiometry. In [37], samples were examined, each deposited with varying O_2_ flows, yet all displayed refractive indices similar to those of SiO_2_. This implied that these samples were likely deposited using high O_2_ flows that had a minimal impact on the stoichiometry.

However, it is interesting to note that the wavenumber at which n  = 1 could slightly vary was between 1322 and 1364 cm^−1^ (7.56 and 7.33 μm), corresponding to the values of the samples deposited with 40 and 120 sccm of O_2_. The sample deposited with 30 sccm of O_2_ never had a real part of the refractive index lower than one. However, it approached values close to one, indicating that the wavelength at which the real part of the refractive index equals one could potentially be controlled with flow rates between 30 and 40 sccm of O_2_. Therefore, the samples were subsequently deposited using an O_2_ flow slightly higher than 30 sccm. Additionally, the values of the dielectric permittivities are provided in Figures S2 and S3 of the Supplementary Material.

**Figure 5:**
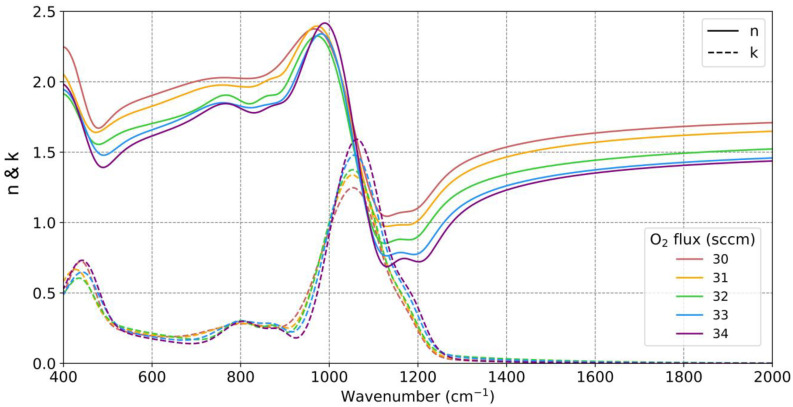
Complex refractive indices for SiO_x_ samples deposited with intermediate reactive gas flows: real part n (solid line) and imaginary part k (dotted line).

Following the same procedure as before, the samples were deposited with flows ranging from 31 to 34 sccm of O_2_. The fitting parameters for the dielectric permittivity using the Brendel–Bormann oscillator model are shown in Table 2. Likewise, Figure 5 displays the refractive indices obtained for these samples. A clear trend can be observed as the O_2_ flow increased, and this series of samples demonstrated the excellent reproducibility of the deposition process and the precise control of O_2_ flow achieved through the MFC. Starting from a flow of 31 sccm, regions with a real part of the refractive index lower than one appeared. For the case of 31 sccm, the cutoff point with n = 1 occurred at 1194 cm^−1^ (8.375 μm), with a value of $\tilde{n}$ = 1.000 + 0.289i. In this case, the imaginary part had a significant value, but it would allow the production of an anti-reflective coating with a residual reflectance of 2% under a normal incidence. In the case of 32 sccm, the cutoff occurred at 1228 cm^−1^ (8.143 μm) with a value of $\tilde{n}$ = 1.007 + 0.126i. These results demonstrated how by modulating the O_2_ flow during SiO_x_ deposition, we could customize the stoichiometry and adjust the refractive indices as desired. The values of the dielectric permittivity for these samples can be found in Figures S4 and S5 of the Supplementary Material.

**Figure 6:**
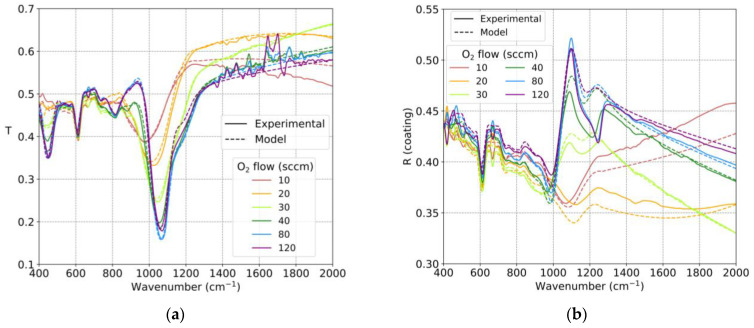
(**a**) Experimental transmission spectra (solid line) and spectra calculated with the obtained refractive indices (dotted line) for SiO_x_ samples deposited with different reactive gas flows. (**b**) Experimental reflectance spectra on the coated side (solid line) and spectra calculated with the obtained refractive indices (dotted line) for SiO_x_ samples deposited with different reactive gas flows.

Figure 6a shows the transmittance spectra of the fabricated samples, while Figure 6b presents the reflectance spectra from the coating side (the reflectance spectrum from the substrate side is included in Figure S6 of the Supplementary Material). In Figure 6, the excellent agreement between the experimental measurements and the calculated values based on the obtained refractive indices can be observed. The drop in transmission in the characteristic absorption bands of SiO_x_ is noticeable, with greater reductions for samples deposited with higher flows of O_2_. Additionally, there was an absorption peak around 600 cm^−1^ corresponding to crystalline Si originating from the substrate. It is also worth noting that the experimental measurements exhibited considerable noise between 1400 and 1800 cm^−1^ due to the absorption bands of gases present in the atmosphere. Regarding the reflectance values, a good agreement was also observed between the experimental measurements and the results calculated from the refractive indices using the employed model. However, it should be emphasized that the vertical scale was greatly amplified (the transmittance and reflectance spectra for the samples deposited with an O_2_ flow between 31 and 34 sccm are shown in Figures S7–S9 of the Supplementary Material). These results showed great agreement between the experimental values and the values calculated with the obtained refractive indices. The complex refractive index values for all the samples can be found in Table S1 of the Supplementary Material.

## 5. Conclusions

The results of this study demonstrated the controllability of SiO_x_ thin film stoichiometry by adjusting the introduced O_2_ flow during the deposition process. It was crucial to carefully select an appropriate range of flow rates to effectively vary the stoichiometry. The refractive indices of SiO_x_ thin films with different stoichiometries were determined using the Brendel–Bormann oscillator model. The findings highlighted the ability to modulate both the real part of the refractive index across the entire spectrum and the imaginary part in the absorption regions. Additionally, this study revealed the potential to modify the cutoff wavelength, where the real part of the refractive index equals unity. Furthermore, the agreement between the theoretically calculated transmittance and reflectance spectra based on the refractive indices and the experimentally obtained spectra was confirmed.

## Figures and Tables

**Table 1:** Brendel–Bormann oscillator parameters for SiO_x_ deposited at different O_2_ flows between 10 and 120 sccm.

O_2_ Flow	$\varepsilon_{\infty }$ (F/m)	$\nu_{oj}$ (cm^−1^)	$\nu_{pj}$ (cm^−1^)	$\nu_{\tau j}$ (cm^−1^)	$\sigma_{j}$ (cm^−1^)
10	9.50	400	71	0.89	34
		547	609	3.82	414
		978	430	0.58	69
		1059	247	5.62	60
20	6.95	416	119	2.50	13
		584	597	2.38	407
		1005	508	0.99	62
		1073	269	4.46	59
30	3.20	434	200	1.24	30
		523	528	4.78	412
		827	286	3.15	85
		1030	650	0.76	57
		1166	203	4.14	37
40	2.20	439	283	5.5	27
		551	402	37.0	259
		809	208	11.5	40
		928	200	16.5	44
		1046	664	4.0	39
		1164	293	5.5	44
80	2.05	231	436	87.9	420
		446	265	8.5	20
		813	191	17.2	26
		1052	690	5.0	34
		1171	281	10.6	42
120	1.90	313	456	2.5	580
		442	277	0.74	21
		813	171	0.04	28
		1049	662	0.01	35
		1172	216	3.5	42

**Table 2:** Brendel–Bormann oscillator parameters for SiO_x_ deposited at different O_2_ flows between 31 and 34 sccm.

O_2_ Flow	$\varepsilon_{\infty }$ (F/m)	$\nu_{oj}$ (cm^−1^)	$\nu_{pj}$ (cm^−1^)	$\nu_{\tau j}$ (cm^−1^)	$\sigma_{j}$ (cm^−1^)
31	3.0	423	184	6.5	33
		462	548	0.1	448
		812	258	14.8	68
		881	89	11.9	17
		1028	651	5.8	51
		1156	231	7.7	40
32	2.6	432	153	0.1	32
		537	590	0.1	564
		800	182	9.8	35
		874	126	1.5	23
		1027	627	0.1	51
		1158	202	0.1	38
33	2.4	437	189	3.2	36
		521	525	6.8	501
		805	224	14.0	51
		882	130	10.1	26
		1031	636	5.3	46
		1158	227	5.0	41
34	2.33	435	207	0.1	36
		478	484	0.1	443
		807	214	8.8	44
		890	136	4.0	26
		1036	644	0.6	44
		1163	244	3.1	43

## Data Availability

Data are available in the Supplementary Material.

## Supplementary Materials

The following supporting information can be downloaded at: https://www.mdpi.com/article/10.3390/nano13202749/s1, Figure S1: Cross-section images of SiO_x_ thin films deposited at different reactive gas flows; Figure S2: Real part of the relative dielectric permittivity in SiO_x_ samples deposited with different reactive gas flows; Figure S3: Imaginary part of the relative dielectric permittivity in SiO_x_ samples deposited with different reactive gas flows; Figure S4: Real part of relative dielectric permittivity in samples deposited with intermediate reactive gas flows; Figure S5: Imaginary part of relative dielectric permittivity in samples deposited with intermediate reactive gas flows; Figure S6: Experimental reflectance spectra on the substrate side (solid line) and calculated with the refractive indices obtained (dotted line) for SiO_x_ samples deposited with different reactive gas flows; Figure S7: Experimental transmission spectra (solid line) and calculated with the refractive indices obtained (dotted line) for SiO_x_ samples deposited with intermediate reactive gas flows; Figure S8: Experimental reflectance spectra on the coating side (solid line) and calculated with the refractive indices obtained (dotted line) for SiO_x_ samples deposited with intermediate reactive gas flows; Figure S9: Experimental reflectance spectra on the substrate side (solid line) and calculated with the refractive indices obtained (dotted line) for SiO_x_ samples deposited with intermediate reactive gas flows; Table S1: Complex refractive indices obtained. n is the real part, k is the imaginary part, the number after n and k refers to the O_2_ flow (in sccm) with which the sample was deposited.
