# Supplementary material for: Optical Properties in Mid-Infrared Range of Silicon Oxide Thin Films with Different Stoichiometries
Source: Nanomaterials (Basel). 2023 Oct 12;13(20):2749. doi: 10.3390/nano13202749 (PMC10609603; doi:10.3390/nano13202749)
Supplement: Supplementary file 1 [file nanomaterials-13-02749-s001.zip › nanomaterials-2645522-supplementary.pdf]

Supplementary Material

Optical Properties in Mid-Infrared Range of Silicon Oxide Thin Films with Different Stoichiometries

Natalia Herguedas, Enrique Carretero

Departamento de Física Aplicada, Universidad de Zaragoza, C/Pedro Cerbuna, 12, 50009 Zaragoza, Spain

Contents

Figure S1: Cross section images of SiO_x thin films deposited at different reactive gas flows.

Figure S2: Real part of the relative dielectric permittivity in SiO_x samples deposited with different reactive gas flows.

Figure S3: Imaginary part of the relative dielectric permittivity in SiO_x samples deposited with different reactive gas flows.

Figure S4: Real part of relative dielectric permittivity in samples deposited with intermediate reactive gas flows.

Figure S5: Imaginary part of relative dielectric permittivity in samples deposited with intermediate reactive gas flows.

Figure S6: Experimental reflectance spectra on the substrate side (solid line) and calculated with the refractive indices obtained (dotted line) for SiO_x samples deposited with different reactive gas flows.

Figure S7: Experimental transmission spectra (solid line) and calculated with the refractive indices obtained (dotted line) for SiO_x samples deposited with intermediate reactive gas flows.

Figure S8: Experimental reflectance spectra on the coating side (solid line) and calculated with the refractive indices obtained (dotted line) for SiO_x samples deposited with intermediate reactive gas flows.

Figure S9: Experimental reflectance spectra on the substrate side (solid line) and calculated with the refractive indices obtained (dotted line) for SiO_x samples deposited with intermediate reactive gas flows.

Table S1: Complex refractive indices obtained. n is the real part, k is the imaginary part, the number after n and k refers to the O_2 flow (in sccm) with which the sample was deposited.

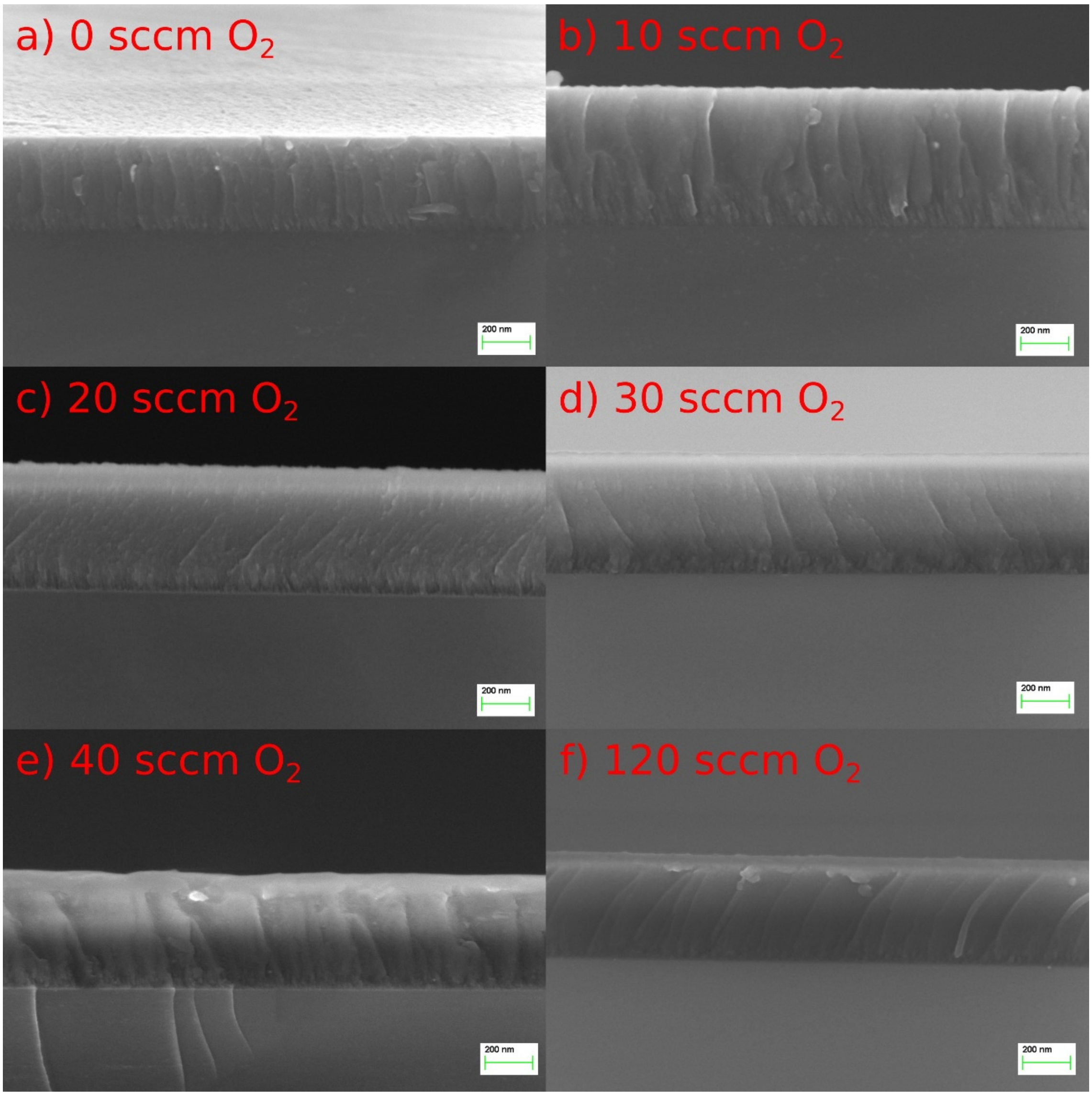

Figure S1: Cross section images of SiO_x thin films deposited at different reactive gas flows.

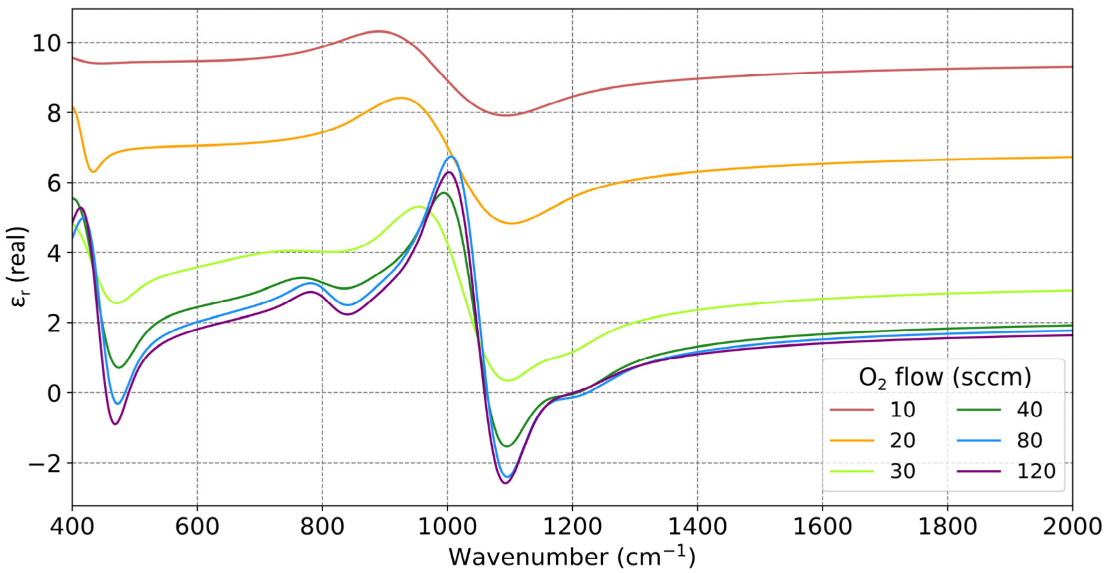

Figure S2: Real part of the relative dielectric permittivity in SiO_x samples deposited with different reactive gas flows.

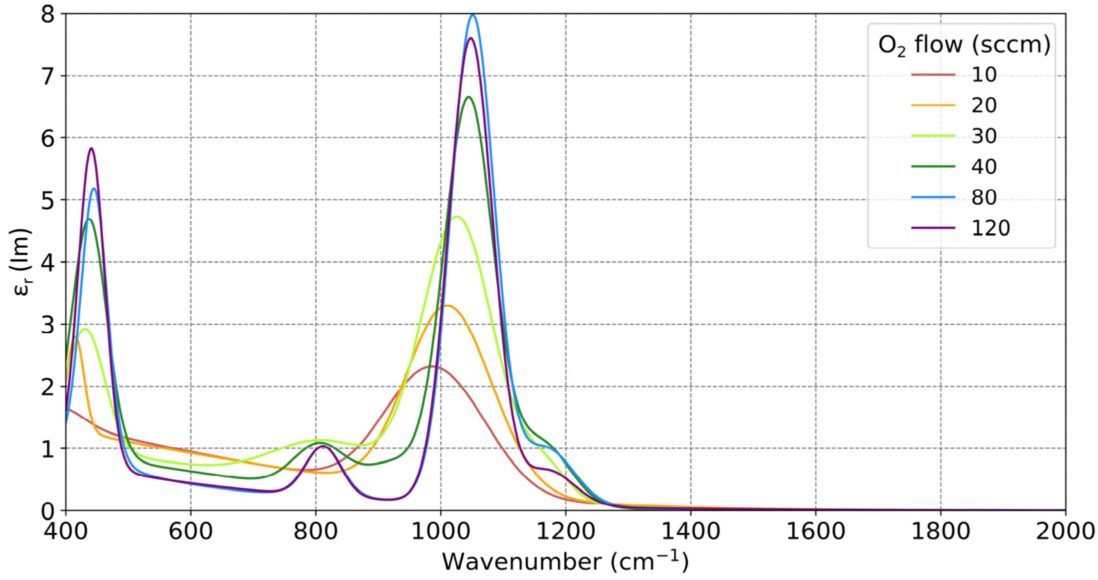

Figure S3: Imaginary part of the relative dielectric permittivity in SiO_x samples deposited with different reactive gas flows.

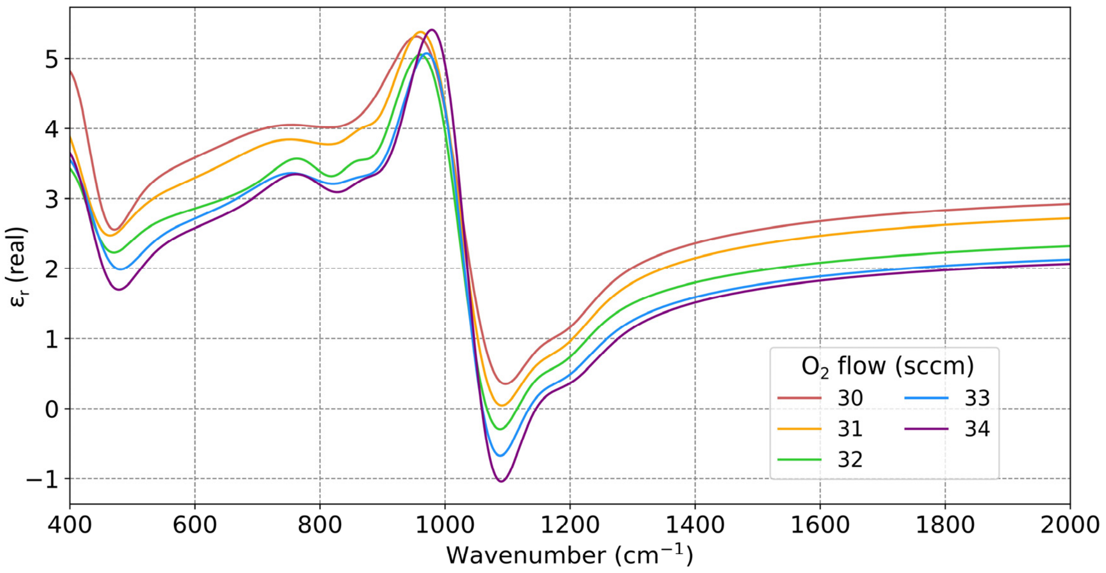

Figure S4: Real part of relative dielectric permittivity in samples deposited with intermediate reactive gas flows.

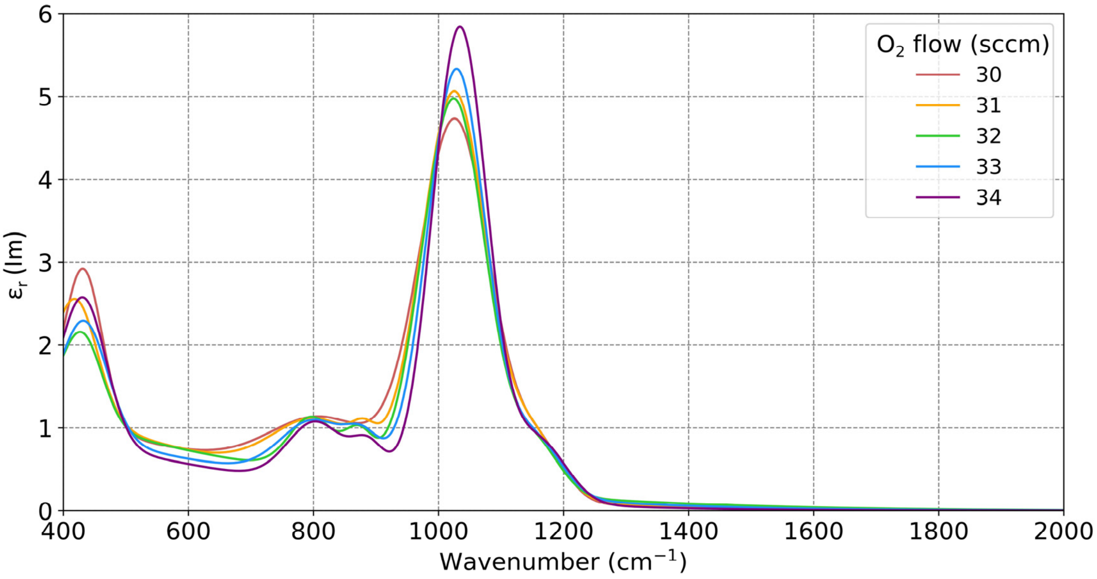

Figure S5: Imaginary part of relative dielectric permittivity in samples deposited with intermediate reactive gas flows.

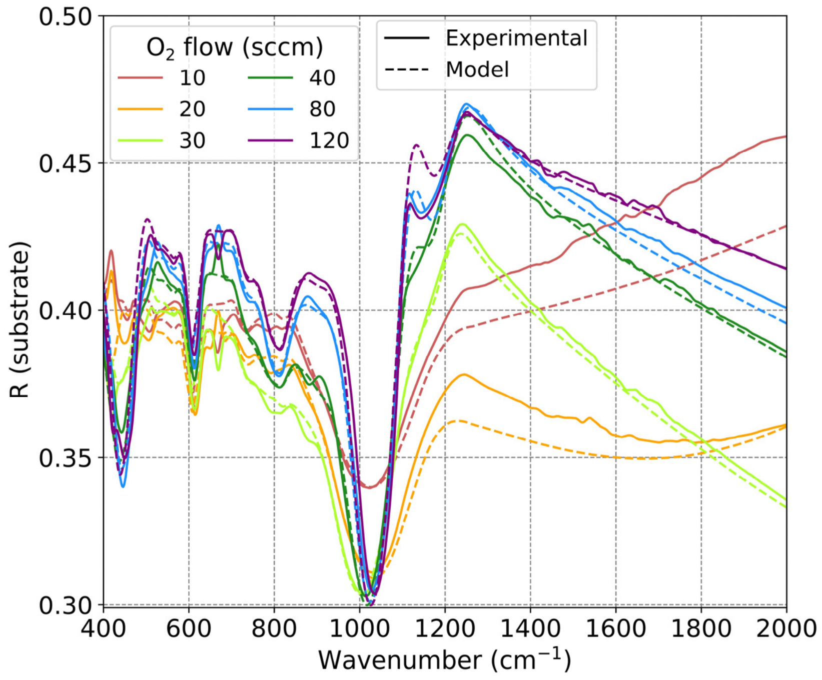

Figure S6: Experimental reflectance spectra on the substrate side (solid line) and calculated with the refractive indices obtained (dotted line) for SiO_x samples deposited with different reactive gas flows.

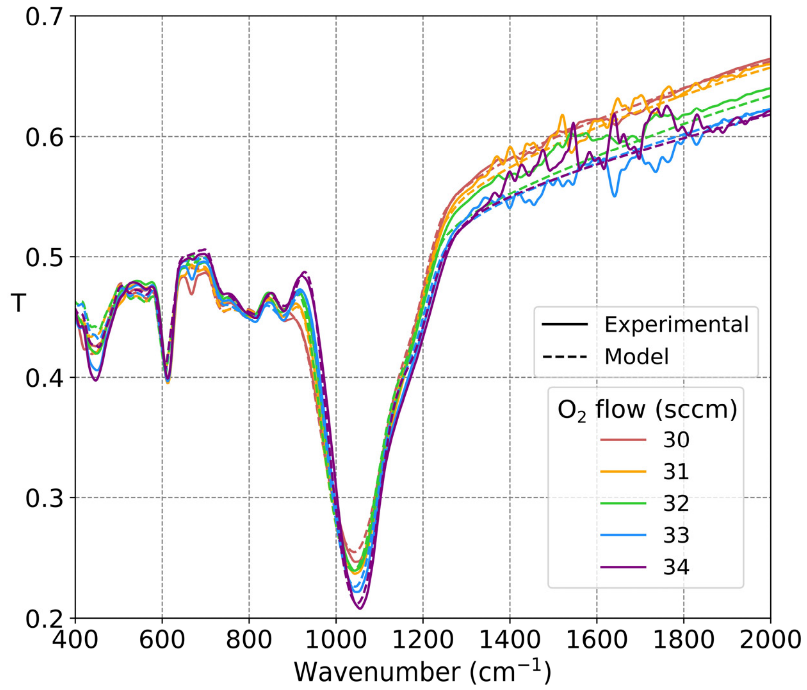

Figure S7: Experimental transmission spectra (solid line) and calculated with the refractive indices obtained (dotted line) for SiO_x samples deposited with intermediate reactive gas flows.

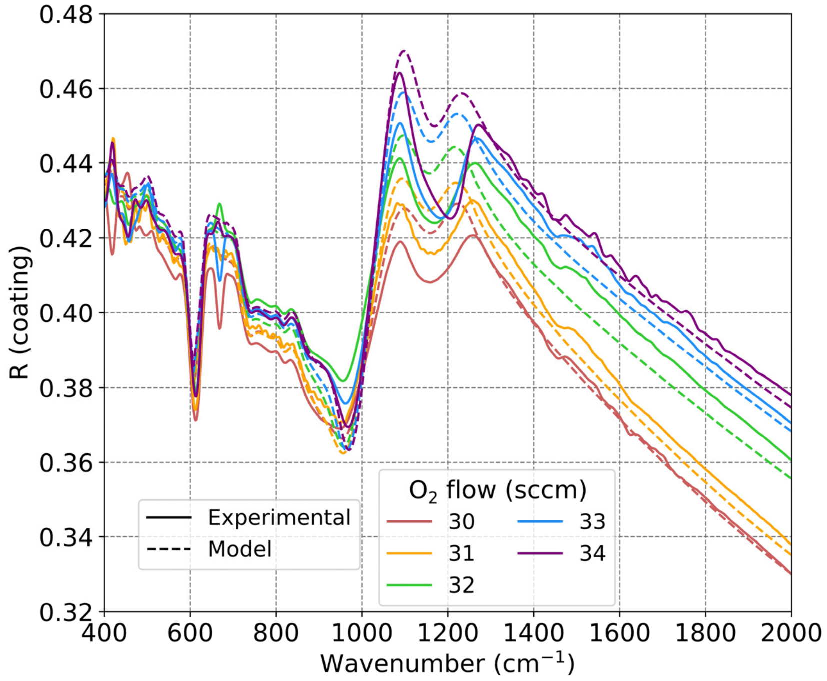

Figure S8: Experimental reflectance spectra on the coating side (solid line) and calculated with the refractive indices obtained (dotted line) for SiO_x samples deposited with intermediate reactive gas flows.

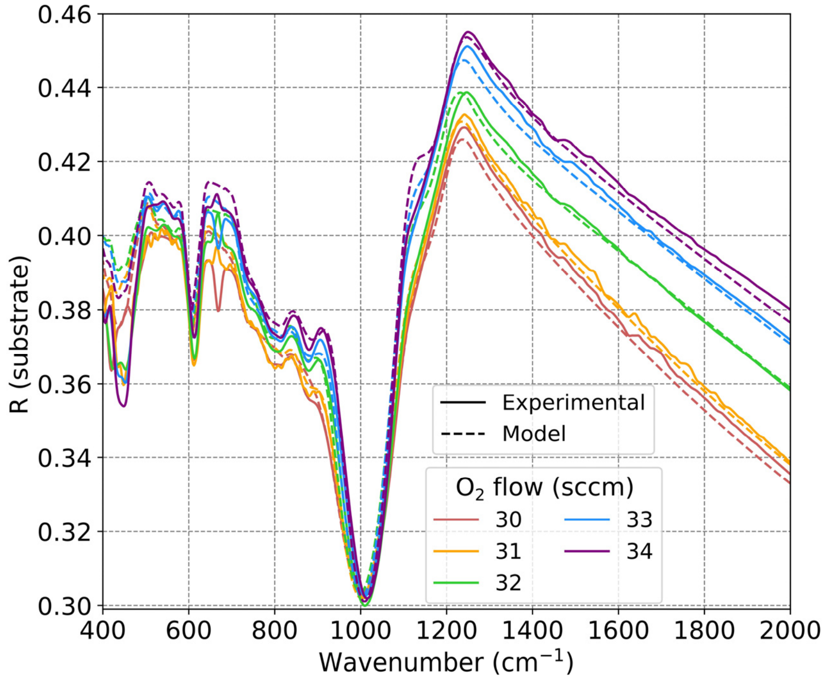

Figure S9: Experimental reflectance spectra on the substrate side (solid line) and calculated with the refractive indices obtained (dotted line) for SiO_x samples deposited with intermediate reactive gas flows.

Table S1: Complex refractive indices obtained. n is the real part, k is the imaginary part, the number after n and k refers to the O_2 flow (in sccm) with which the sample was deposited.

Wavenumber (cm^{-1})	Wavelength (µm)	n10	k10	n20	k20	n30	k30	n40	k40	n80	k80	n120	k120	n31	k31	n32	k32	n33	k33	n34	k34
2000	5.000	3.051	0.000	2.594	0.000	1.710	0.000	1.387	0.001	1.332	0.001	1.281	0.001	1.649	0.000	1.523	0.002	1.459	0.001	1.438	0.000
1998	5.005	3.051	0.000	2.594	0.000	1.710	0.000	1.387	0.001	1.332	0.001	1.281	0.001	1.648	0.000	1.523	0.002	1.459	0.001	1.437	0.000
1996	5.010	3.051	0.000	2.594	0.000	1.710	0.000	1.387	0.001	1.332	0.001	1.280	0.001	1.648	0.000	1.523	0.002	1.459	0.001	1.437	0.000
1994	5.015	3.051	0.000	2.594	0.000	1.709	0.000	1.386	0.001	1.331	0.001	1.280	0.001	1.648	0.000	1.522	0.002	1.458	0.001	1.437	0.000
1992	5.020	3.051	0.000	2.593	0.000	1.709	0.000	1.386	0.001	1.331	0.001	1.280	0.001	1.648	0.000	1.522	0.002	1.458	0.001	1.437	0.000
1990	5.025	3.050	0.000	2.593	0.000	1.709	0.000	1.386	0.001	1.331	0.001	1.280	0.001	1.647	0.000	1.522	0.002	1.458	0.001	1.437	0.000
1988	5.030	3.050	0.000	2.593	0.000	1.709	0.000	1.385	0.001	1.331	0.001	1.279	0.001	1.647	0.000	1.522	0.002	1.458	0.001	1.436	0.000
1986	5.035	3.050	0.000	2.593	0.000	1.709	0.000	1.385	0.001	1.330	0.001	1.279	0.001	1.647	0.000	1.521	0.002	1.457	0.001	1.436	0.000
1984	5.040	3.050	0.000	2.593	0.000	1.708	0.000	1.385	0.001	1.330	0.001	1.279	0.001	1.647	0.000	1.521	0.002	1.457	0.001	1.436	0.000
1982	5.045	3.050	0.000	2.593	0.000	1.708	0.000	1.385	0.001	1.330	0.001	1.279	0.001	1.647	0.000	1.521	0.002	1.457	0.001	1.436	0.000
1980	5.051	3.050	0.000	2.593	0.000	1.708	0.000	1.384	0.001	1.330	0.001	1.278	0.001	1.646	0.000	1.521	0.002	1.457	0.001	1.435	0.000
1978	5.056	3.050	0.000	2.593	0.000	1.708	0.000	1.384	0.001	1.329	0.001	1.278	0.001	1.646	0.001	1.520	0.002	1.456	0.001	1.435	0.000
1976	5.061	3.050	0.000	2.593	0.000	1.707	0.000	1.384	0.001	1.329	0.001	1.278	0.001	1.646	0.001	1.520	0.003	1.456	0.001	1.435	0.000
1974	5.066	3.050	0.000	2.592	0.000	1.707	0.000	1.384	0.001	1.329	0.001	1.277	0.001	1.646	0.001	1.520	0.003	1.456	0.001	1.435	0.000
1972	5.071	3.050	0.000	2.592	0.000	1.707	0.000	1.383	0.001	1.328	0.001	1.277	0.001	1.645	0.001	1.520	0.003	1.456	0.001	1.434	0.000
1970	5.076	3.050	0.000	2.592	0.000	1.707	0.000	1.383	0.001	1.328	0.001	1.277	0.001	1.645	0.001	1.519	0.003	1.455	0.001	1.434	0.000
1968	5.081	3.050	0.000	2.592	0.000	1.707	0.000	1.383	0.001	1.328	0.001	1.277	0.001	1.645	0.001	1.519	0.003	1.455	0.001	1.434	0.000
1966	5.086	3.049	0.000	2.592	0.000	1.706	0.000	1.382	0.001	1.327	0.001	1.276	0.001	1.645	0.001	1.519	0.003	1.455	0.001	1.433	0.000
1964	5.092	3.049	0.000	2.592	0.000	1.706	0.000	1.382	0.001	1.327	0.001	1.276	0.001	1.644	0.001	1.519	0.003	1.455	0.001	1.433	0.000
1962	5.097	3.049	0.000	2.592	0.000	1.706	0.000	1.382	0.001	1.327	0.001	1.276	0.001	1.644	0.001	1.518	0.003	1.454	0.001	1.433	0.000
1960	5.102	3.049	0.000	2.592	0.000	1.706	0.000	1.382	0.001	1.327	0.001	1.275	0.001	1.644	0.001	1.518	0.003	1.454	0.001	1.433	0.000
1958	5.107	3.049	0.000	2.591	0.000	1.705	0.000	1.381	0.001	1.326	0.001	1.275	0.001	1.644	0.001	1.518	0.003	1.454	0.001	1.432	0.000
1956	5.112	3.049	0.000	2.591	0.000	1.705	0.000	1.381	0.001	1.326	0.001	1.275	0.001	1.643	0.001	1.518	0.003	1.454	0.001	1.432	0.000
1954	5.118	3.049	0.000	2.591	0.000	1.705	0.000	1.381	0.001	1.326	0.001	1.275	0.001	1.643	0.001	1.517	0.003	1.453	0.001	1.432	0.000
1952	5.123	3.049	0.000	2.591	0.000	1.705	0.000	1.380	0.001	1.325	0.001	1.274	0.001	1.643	0.001	1.517	0.003	1.453	0.001	1.432	0.000
1950	5.128	3.049	0.000	2.591	0.000	1.704	0.000	1.380	0.001	1.325	0.001	1.274	0.001	1.643	0.001	1.517	0.003	1.453	0.001	1.431	0.000
1948	5.133	3.049	0.000	2.591	0.000	1.704	0.000	1.380	0.001	1.325	0.001	1.274	0.001	1.642	0.001	1.516	0.003	1.452	0.001	1.431	0.000
1946	5.139	3.049	0.000	2.591	0.000	1.704	0.000	1.380	0.001	1.324	0.001	1.273	0.001	1.642	0.001	1.516	0.003	1.452	0.001	1.431	0.000
1944	5.144	3.048	0.000	2.591	0.000	1.704	0.000	1.379	0.001	1.324	0.001	1.273	0.001	1.642	0.001	1.516	0.003	1.452	0.002	1.431	0.000
1942	5.149	3.048	0.000	2.590	0.000	1.703	0.000	1.379	0.001	1.324	0.001	1.273	0.001	1.642	0.001	1.516	0.003	1.452	0.002	1.430	0.000
1940	5.155	3.048	0.000	2.590	0.000	1.703	0.000	1.379	0.001	1.324	0.001	1.272	0.001	1.641	0.001	1.515	0.003	1.451	0.002	1.430	0.000

Wavenumber (cm^{-1})	Wavelength (µm)	n10	k10	n20	k20	n30	k30	n40	k40	n80	k80	n120	k120	n31	k31	n32	k32	n33	k33	n34	k34
1938	5.160	3.048	0.000	2.590	0.000	1.703	0.000	1.378	0.001	1.323	0.001	1.272	0.001	1.641	0.001	1.515	0.003	1.451	0.002	1.430	0.000
1936	5.165	3.048	0.000	2.590	0.000	1.703	0.000	1.378	0.001	1.323	0.001	1.272	0.001	1.641	0.001	1.515	0.003	1.451	0.002	1.429	0.000
1934	5.171	3.048	0.000	2.590	0.000	1.703	0.000	1.378	0.001	1.323	0.001	1.271	0.001	1.641	0.001	1.515	0.003	1.450	0.002	1.429	0.000
1932	5.176	3.048	0.000	2.590	0.000	1.702	0.000	1.377	0.001	1.322	0.001	1.271	0.001	1.640	0.001	1.514	0.003	1.450	0.002	1.429	0.000
1930	5.181	3.048	0.000	2.590	0.000	1.702	0.000	1.377	0.001	1.322	0.002	1.271	0.001	1.640	0.001	1.514	0.003	1.450	0.002	1.429	0.000
1928	5.187	3.048	0.000	2.590	0.000	1.702	0.000	1.377	0.001	1.322	0.002	1.271	0.001	1.640	0.001	1.514	0.003	1.450	0.002	1.428	0.000
1926	5.192	3.048	0.000	2.589	0.000	1.702	0.000	1.376	0.001	1.321	0.002	1.270	0.001	1.640	0.001	1.513	0.003	1.449	0.002	1.428	0.000
1924	5.198	3.048	0.000	2.589	0.000	1.701	0.000	1.376	0.001	1.321	0.002	1.270	0.001	1.639	0.001	1.513	0.003	1.449	0.002	1.428	0.000
1922	5.203	3.047	0.000	2.589	0.000	1.701	0.000	1.376	0.001	1.321	0.002	1.270	0.001	1.639	0.001	1.513	0.003	1.449	0.002	1.427	0.000
1920	5.208	3.047	0.000	2.589	0.000	1.701	0.000	1.376	0.001	1.320	0.002	1.269	0.001	1.639	0.001	1.513	0.003	1.448	0.002	1.427	0.000
1918	5.214	3.047	0.000	2.589	0.000	1.701	0.000	1.375	0.001	1.320	0.002	1.269	0.001	1.639	0.001	1.512	0.003	1.448	0.002	1.427	0.000
1916	5.219	3.047	0.000	2.589	0.000	1.700	0.000	1.375	0.001	1.320	0.002	1.269	0.001	1.638	0.001	1.512	0.003	1.448	0.002	1.427	0.000
1914	5.225	3.047	0.000	2.589	0.000	1.700	0.000	1.375	0.001	1.319	0.002	1.268	0.001	1.638	0.001	1.512	0.003	1.448	0.002	1.426	0.000
1912	5.230	3.047	0.000	2.588	0.000	1.700	0.000	1.374	0.001	1.319	0.002	1.268	0.001	1.638	0.001	1.511	0.003	1.447	0.002	1.426	0.000
1910	5.236	3.047	0.000	2.588	0.000	1.699	0.000	1.374	0.001	1.319	0.002	1.268	0.001	1.637	0.001	1.511	0.003	1.447	0.002	1.426	0.000
1908	5.241	3.047	0.000	2.588	0.000	1.699	0.000	1.374	0.001	1.318	0.002	1.267	0.001	1.637	0.001	1.511	0.004	1.447	0.002	1.425	0.000
1906	5.247	3.047	0.000	2.588	0.000	1.699	0.000	1.373	0.001	1.318	0.002	1.267	0.001	1.637	0.001	1.511	0.004	1.446	0.002	1.425	0.000
1904	5.252	3.047	0.000	2.588	0.000	1.699	0.000	1.373	0.001	1.318	0.002	1.267	0.001	1.637	0.001	1.510	0.004	1.446	0.002	1.425	0.000
1902	5.258	3.046	0.000	2.588	0.000	1.698	0.000	1.373	0.001	1.317	0.002	1.266	0.001	1.636	0.001	1.510	0.004	1.446	0.002	1.424	0.000
1900	5.263	3.046	0.000	2.588	0.000	1.698	0.000	1.372	0.001	1.317	0.002	1.266	0.001	1.636	0.001	1.510	0.004	1.445	0.002	1.424	0.000
1898	5.269	3.046	0.000	2.588	0.000	1.698	0.000	1.372	0.001	1.317	0.002	1.266	0.001	1.636	0.001	1.509	0.004	1.445	0.002	1.424	0.000
1896	5.274	3.046	0.000	2.587	0.000	1.698	0.000	1.372	0.001	1.316	0.002	1.265	0.001	1.635	0.001	1.509	0.004	1.445	0.002	1.424	0.000
1894	5.280	3.046	0.000	2.587	0.000	1.697	0.000	1.371	0.001	1.316	0.002	1.265	0.001	1.635	0.001	1.509	0.004	1.445	0.002	1.423	0.000
1892	5.285	3.046	0.000	2.587	0.000	1.697	0.000	1.371	0.001	1.316	0.002	1.265	0.001	1.635	0.001	1.508	0.004	1.444	0.002	1.423	0.000
1890	5.291	3.046	0.000	2.587	0.000	1.697	0.000	1.371	0.001	1.315	0.002	1.264	0.001	1.635	0.001	1.508	0.004	1.444	0.002	1.423	0.000
1888	5.297	3.046	0.000	2.587	0.000	1.697	0.000	1.370	0.001	1.315	0.002	1.264	0.001	1.634	0.001	1.508	0.004	1.444	0.002	1.422	0.000
1886	5.302	3.046	0.000	2.587	0.000	1.696	0.000	1.370	0.001	1.315	0.002	1.264	0.001	1.634	0.001	1.508	0.004	1.443	0.002	1.422	0.000
1884	5.308	3.046	0.000	2.587	0.000	1.696	0.000	1.370	0.001	1.314	0.002	1.263	0.001	1.634	0.001	1.507	0.004	1.443	0.002	1.422	0.000
1882	5.313	3.045	0.000	2.586	0.000	1.696	0.000	1.369	0.001	1.314	0.002	1.263	0.001	1.633	0.001	1.507	0.004	1.443	0.002	1.421	0.000
1880	5.319	3.045	0.000	2.586	0.000	1.695	0.000	1.369	0.001	1.313	0.002	1.263	0.001	1.633	0.001	1.507	0.004	1.442	0.002	1.421	0.001
1878	5.325	3.045	0.000	2.586	0.000	1.695	0.000	1.369	0.001	1.313	0.002	1.262	0.001	1.633	0.001	1.506	0.004	1.442	0.002	1.421	0.001
1876	5.330	3.045	0.000	2.586	0.000	1.695	0.000	1.368	0.001	1.313	0.002	1.262	0.001	1.633	0.001	1.506	0.004	1.442	0.002	1.420	0.001

Wavenumber (cm^{-1})	Wavelength (μm)	n10	k10	n20	k20	n30	k30	n40	k40	n80	k80	n120	k120	n31	k31	n32	k32	n33	k33	n34	k34
1874	5.336	3.045	0.000	2.586	0.000	1.695	0.000	1.368	0.001	1.312	0.002	1.261	0.001	1.632	0.001	1.506	0.004	1.441	0.002	1.420	0.001
1872	5.342	3.045	0.000	2.586	0.000	1.694	0.000	1.367	0.001	1.312	0.002	1.261	0.001	1.632	0.001	1.505	0.004	1.441	0.002	1.420	0.001
1870	5.348	3.045	0.000	2.585	0.000	1.694	0.000	1.367	0.001	1.312	0.002	1.261	0.001	1.632	0.001	1.505	0.004	1.441	0.002	1.419	0.001
1868	5.353	3.045	0.000	2.585	0.000	1.694	0.000	1.367	0.001	1.311	0.002	1.260	0.001	1.631	0.001	1.505	0.004	1.440	0.002	1.419	0.001
1866	5.359	3.045	0.000	2.585	0.000	1.693	0.000	1.366	0.001	1.311	0.002	1.260	0.001	1.631	0.001	1.504	0.004	1.440	0.002	1.419	0.001
1864	5.365	3.044	0.000	2.585	0.000	1.693	0.001	1.366	0.001	1.310	0.002	1.260	0.001	1.631	0.001	1.504	0.004	1.440	0.002	1.418	0.001
1862	5.371	3.044	0.000	2.585	0.000	1.693	0.001	1.366	0.001	1.310	0.002	1.259	0.001	1.630	0.001	1.504	0.004	1.439	0.002	1.418	0.001
1860	5.376	3.044	0.000	2.585	0.000	1.693	0.001	1.365	0.001	1.310	0.002	1.259	0.001	1.630	0.001	1.503	0.004	1.439	0.002	1.418	0.001
1858	5.382	3.044	0.000	2.585	0.001	1.692	0.001	1.365	0.001	1.309	0.002	1.259	0.002	1.630	0.001	1.503	0.005	1.439	0.002	1.417	0.001
1856	5.388	3.044	0.000	2.584	0.001	1.692	0.001	1.365	0.001	1.309	0.002	1.258	0.002	1.630	0.001	1.503	0.005	1.438	0.002	1.417	0.001
1854	5.394	3.044	0.000	2.584	0.001	1.692	0.001	1.364	0.001	1.309	0.002	1.258	0.002	1.629	0.001	1.502	0.005	1.438	0.002	1.417	0.001
1852	5.400	3.044	0.000	2.584	0.001	1.691	0.001	1.364	0.001	1.308	0.002	1.257	0.002	1.629	0.001	1.502	0.005	1.438	0.002	1.416	0.001
1850	5.405	3.044	0.000	2.584	0.001	1.691	0.001	1.363	0.001	1.308	0.002	1.257	0.002	1.629	0.001	1.502	0.005	1.437	0.002	1.416	0.001
1848	5.411	3.044	0.000	2.584	0.001	1.691	0.001	1.363	0.001	1.307	0.002	1.257	0.002	1.628	0.001	1.501	0.005	1.437	0.002	1.416	0.001
1846	5.417	3.043	0.000	2.584	0.001	1.691	0.001	1.363	0.001	1.307	0.002	1.256	0.002	1.628	0.001	1.501	0.005	1.437	0.003	1.415	0.001
1844	5.423	3.043	0.000	2.583	0.001	1.690	0.001	1.362	0.001	1.307	0.002	1.256	0.002	1.628	0.001	1.501	0.005	1.436	0.003	1.415	0.001
1842	5.429	3.043	0.000	2.583	0.001	1.690	0.001	1.362	0.001	1.306	0.002	1.255	0.002	1.627	0.001	1.500	0.005	1.436	0.003	1.415	0.001
1840	5.435	3.043	0.000	2.583	0.001	1.690	0.001	1.362	0.001	1.306	0.002	1.255	0.002	1.627	0.001	1.500	0.005	1.435	0.003	1.414	0.001
1838	5.441	3.043	0.000	2.583	0.001	1.689	0.001	1.361	0.001	1.305	0.002	1.255	0.002	1.627	0.001	1.500	0.005	1.435	0.003	1.414	0.001
1836	5.447	3.043	0.000	2.583	0.001	1.689	0.001	1.361	0.001	1.305	0.002	1.254	0.002	1.626	0.001	1.499	0.005	1.435	0.003	1.414	0.001
1834	5.453	3.043	0.000	2.583	0.001	1.689	0.001	1.360	0.001	1.305	0.002	1.254	0.002	1.626	0.001	1.499	0.005	1.434	0.003	1.413	0.001
1832	5.459	3.043	0.000	2.583	0.001	1.688	0.001	1.360	0.001	1.304	0.002	1.254	0.002	1.626	0.001	1.499	0.005	1.434	0.003	1.413	0.001
1830	5.464	3.043	0.001	2.582	0.001	1.688	0.001	1.360	0.001	1.304	0.002	1.253	0.002	1.625	0.001	1.498	0.005	1.434	0.003	1.413	0.001
1828	5.470	3.042	0.001	2.582	0.001	1.688	0.001	1.359	0.001	1.303	0.002	1.253	0.002	1.625	0.001	1.498	0.005	1.433	0.003	1.412	0.001
1826	5.476	3.042	0.001	2.582	0.001	1.687	0.001	1.359	0.001	1.303	0.002	1.252	0.002	1.625	0.001	1.498	0.005	1.433	0.003	1.412	0.001
1824	5.482	3.042	0.001	2.582	0.001	1.687	0.001	1.358	0.001	1.302	0.002	1.252	0.002	1.624	0.001	1.497	0.005	1.433	0.003	1.411	0.001
1822	5.488	3.042	0.001	2.582	0.001	1.687	0.001	1.358	0.001	1.302	0.002	1.251	0.002	1.624	0.001	1.497	0.005	1.432	0.003	1.411	0.001
1820	5.495	3.042	0.001	2.582	0.001	1.686	0.001	1.358	0.001	1.302	0.002	1.251	0.002	1.624	0.001	1.497	0.005	1.432	0.003	1.411	0.001
1818	5.501	3.042	0.001	2.581	0.001	1.686	0.001	1.357	0.001	1.301	0.002	1.251	0.002	1.623	0.001	1.496	0.005	1.431	0.003	1.410	0.001
1816	5.507	3.042	0.001	2.581	0.001	1.686	0.001	1.357	0.001	1.301	0.002	1.250	0.002	1.623	0.001	1.496	0.005	1.431	0.003	1.410	0.001
1814	5.513	3.042	0.001	2.581	0.001	1.685	0.001	1.356	0.001	1.300	0.002	1.250	0.002	1.623	0.001	1.495	0.006	1.431	0.003	1.410	0.001
1812	5.519	3.041	0.001	2.581	0.001	1.685	0.001	1.356	0.001	1.300	0.002	1.249	0.002	1.622	0.001	1.495	0.006	1.430	0.003	1.409	0.001

Wavenumber (cm^{-1})	Wavelength (μm)	n10	k10	n20	k20	n30	k30	n40	k40	n80	k80	n120	k120	n31	k31	n32	k32	n33	k33	n34	k34
1810	5.525	3.041	0.001	2.581	0.001	1.685	0.001	1.355	0.001	1.299	0.002	1.249	0.002	1.622	0.001	1.495	0.006	1.430	0.003	1.409	0.001
1808	5.531	3.041	0.001	2.580	0.001	1.684	0.001	1.355	0.001	1.299	0.002	1.249	0.002	1.622	0.001	1.494	0.006	1.430	0.003	1.408	0.001
1806	5.537	3.041	0.001	2.580	0.001	1.684	0.001	1.355	0.001	1.299	0.002	1.248	0.002	1.621	0.001	1.494	0.006	1.429	0.003	1.408	0.001
1804	5.543	3.041	0.001	2.580	0.001	1.684	0.001	1.354	0.001	1.298	0.002	1.248	0.002	1.621	0.001	1.494	0.006	1.429	0.003	1.408	0.001
1802	5.549	3.041	0.001	2.580	0.001	1.683	0.001	1.354	0.001	1.298	0.002	1.247	0.002	1.620	0.001	1.493	0.006	1.428	0.003	1.407	0.001
1800	5.556	3.041	0.001	2.580	0.001	1.683	0.001	1.353	0.001	1.297	0.002	1.247	0.002	1.620	0.001	1.493	0.006	1.428	0.003	1.407	0.001
1798	5.562	3.041	0.001	2.580	0.001	1.683	0.001	1.353	0.001	1.297	0.002	1.246	0.002	1.620	0.001	1.492	0.006	1.428	0.003	1.406	0.001
1796	5.568	3.040	0.001	2.579	0.001	1.682	0.001	1.352	0.001	1.296	0.002	1.246	0.002	1.619	0.001	1.492	0.006	1.427	0.003	1.406	0.001
1794	5.574	3.040	0.001	2.579	0.001	1.682	0.001	1.352	0.001	1.296	0.002	1.246	0.002	1.619	0.001	1.492	0.006	1.427	0.003	1.406	0.001
1792	5.580	3.040	0.001	2.579	0.001	1.682	0.001	1.352	0.001	1.295	0.002	1.245	0.002	1.619	0.001	1.491	0.006	1.426	0.003	1.405	0.001
1790	5.587	3.040	0.001	2.579	0.001	1.681	0.001	1.351	0.001	1.295	0.002	1.245	0.002	1.618	0.001	1.491	0.006	1.426	0.003	1.405	0.001
1788	5.593	3.040	0.001	2.579	0.001	1.681	0.001	1.351	0.001	1.294	0.002	1.244	0.002	1.618	0.001	1.491	0.006	1.426	0.003	1.404	0.001
1786	5.599	3.040	0.001	2.579	0.001	1.681	0.001	1.350	0.001	1.294	0.002	1.244	0.002	1.618	0.001	1.490	0.006	1.425	0.003	1.404	0.001
1784	5.605	3.040	0.001	2.578	0.001	1.680	0.001	1.350	0.001	1.294	0.002	1.243	0.002	1.617	0.001	1.490	0.006	1.425	0.003	1.404	0.001
1782	5.612	3.040	0.001	2.578	0.001	1.680	0.001	1.349	0.001	1.293	0.002	1.243	0.002	1.617	0.001	1.489	0.006	1.424	0.003	1.403	0.001
1780	5.618	3.039	0.001	2.578	0.001	1.680	0.001	1.349	0.001	1.293	0.002	1.242	0.002	1.616	0.001	1.489	0.006	1.424	0.004	1.403	0.001
1778	5.624	3.039	0.001	2.578	0.001	1.679	0.001	1.348	0.001	1.292	0.002	1.242	0.002	1.616	0.001	1.489	0.007	1.423	0.004	1.402	0.001
1776	5.631	3.039	0.001	2.578	0.001	1.679	0.001	1.348	0.001	1.292	0.002	1.241	0.002	1.616	0.001	1.488	0.007	1.423	0.004	1.402	0.001
1774	5.637	3.039	0.001	2.577	0.001	1.678	0.001	1.347	0.001	1.291	0.002	1.241	0.002	1.615	0.002	1.488	0.007	1.423	0.004	1.401	0.001
1772	5.643	3.039	0.001	2.577	0.001	1.678	0.001	1.347	0.001	1.291	0.002	1.240	0.002	1.615	0.002	1.487	0.007	1.422	0.004	1.401	0.001
1770	5.650	3.039	0.001	2.577	0.001	1.678	0.001	1.346	0.001	1.290	0.002	1.240	0.002	1.614	0.002	1.487	0.007	1.422	0.004	1.401	0.001
1768	5.656	3.039	0.001	2.577	0.001	1.677	0.001	1.346	0.001	1.290	0.002	1.240	0.002	1.614	0.002	1.487	0.007	1.421	0.004	1.400	0.001
1766	5.663	3.038	0.001	2.577	0.001	1.677	0.001	1.346	0.001	1.289	0.002	1.239	0.002	1.614	0.002	1.486	0.007	1.421	0.004	1.400	0.001
1764	5.669	3.038	0.001	2.576	0.001	1.677	0.001	1.345	0.001	1.289	0.002	1.239	0.002	1.613	0.002	1.486	0.007	1.421	0.004	1.399	0.001
1762	5.675	3.038	0.001	2.576	0.001	1.676	0.001	1.345	0.001	1.288	0.002	1.238	0.002	1.613	0.002	1.485	0.007	1.420	0.004	1.399	0.001
1760	5.682	3.038	0.001	2.576	0.001	1.676	0.001	1.344	0.001	1.288	0.002	1.238	0.002	1.612	0.002	1.485	0.007	1.420	0.004	1.398	0.001
1758	5.688	3.038	0.001	2.576	0.001	1.675	0.001	1.344	0.001	1.287	0.002	1.237	0.003	1.612	0.002	1.484	0.007	1.419	0.004	1.398	0.001
1756	5.695	3.038	0.001	2.576	0.001	1.675	0.001	1.343	0.001	1.287	0.002	1.237	0.003	1.612	0.002	1.484	0.007	1.419	0.004	1.398	0.001
1754	5.701	3.038	0.001	2.575	0.001	1.675	0.001	1.343	0.001	1.286	0.002	1.236	0.003	1.611	0.002	1.484	0.007	1.418	0.004	1.397	0.001
1752	5.708	3.037	0.001	2.575	0.001	1.674	0.001	1.342	0.001	1.286	0.002	1.236	0.003	1.611	0.002	1.483	0.007	1.418	0.004	1.397	0.001
1750	5.714	3.037	0.001	2.575	0.001	1.674	0.001	1.342	0.001	1.285	0.002	1.235	0.003	1.610	0.002	1.483	0.007	1.417	0.004	1.396	0.001
1748	5.721	3.037	0.001	2.575	0.001	1.674	0.001	1.341	0.001	1.285	0.002	1.235	0.003	1.610	0.002	1.482	0.008	1.417	0.004	1.396	0.001

Wavenumber (cm^{-1})	Wavelength (μm)	n10	k10	n20	k20	n30	k30	n40	k40	n80	k80	n120	k120	n31	k31	n32	k32	n33	k33	n34	k34
1746	5.727	3.037	0.001	2.575	0.001	1.673	0.001	1.341	0.001	1.284	0.002	1.234	0.003	1.610	0.002	1.482	0.008	1.416	0.004	1.395	0.001
1744	5.734	3.037	0.001	2.574	0.001	1.673	0.001	1.340	0.001	1.283	0.002	1.234	0.003	1.609	0.002	1.482	0.008	1.416	0.004	1.395	0.001
1742	5.741	3.037	0.001	2.574	0.001	1.672	0.001	1.340	0.001	1.283	0.002	1.233	0.003	1.609	0.002	1.481	0.008	1.416	0.004	1.394	0.001
1740	5.747	3.037	0.001	2.574	0.001	1.672	0.001	1.339	0.001	1.282	0.002	1.233	0.003	1.608	0.002	1.481	0.008	1.415	0.004	1.394	0.001
1738	5.754	3.036	0.001	2.574	0.001	1.671	0.001	1.339	0.001	1.282	0.002	1.232	0.003	1.608	0.002	1.480	0.008	1.415	0.004	1.393	0.001
1736	5.760	3.036	0.001	2.574	0.001	1.671	0.001	1.338	0.001	1.281	0.002	1.231	0.003	1.607	0.002	1.480	0.008	1.414	0.004	1.393	0.001
1734	5.767	3.036	0.001	2.573	0.001	1.671	0.001	1.337	0.001	1.281	0.002	1.231	0.003	1.607	0.002	1.479	0.008	1.414	0.004	1.392	0.001
1732	5.774	3.036	0.001	2.573	0.001	1.670	0.001	1.337	0.001	1.280	0.002	1.230	0.003	1.607	0.002	1.479	0.008	1.413	0.005	1.392	0.001
1730	5.780	3.036	0.001	2.573	0.001	1.670	0.001	1.336	0.001	1.280	0.002	1.230	0.003	1.606	0.002	1.478	0.008	1.413	0.005	1.391	0.001
1728	5.787	3.036	0.001	2.573	0.001	1.669	0.001	1.336	0.001	1.279	0.003	1.229	0.003	1.606	0.002	1.478	0.008	1.412	0.005	1.391	0.001
1726	5.794	3.036	0.001	2.573	0.001	1.669	0.001	1.335	0.001	1.278	0.003	1.229	0.003	1.605	0.002	1.478	0.008	1.412	0.005	1.390	0.001
1724	5.800	3.035	0.001	2.572	0.001	1.669	0.001	1.335	0.001	1.278	0.003	1.228	0.003	1.605	0.002	1.477	0.008	1.411	0.005	1.390	0.001
1722	5.807	3.035	0.001	2.572	0.001	1.668	0.001	1.334	0.001	1.277	0.003	1.228	0.003	1.604	0.002	1.477	0.008	1.411	0.005	1.390	0.001
1720	5.814	3.035	0.001	2.572	0.001	1.668	0.001	1.334	0.001	1.277	0.003	1.227	0.003	1.604	0.002	1.476	0.009	1.410	0.005	1.389	0.002
1718	5.821	3.035	0.001	2.572	0.001	1.667	0.001	1.333	0.001	1.276	0.003	1.227	0.003	1.603	0.002	1.476	0.009	1.410	0.005	1.389	0.002
1716	5.828	3.035	0.001	2.571	0.001	1.667	0.001	1.333	0.001	1.276	0.003	1.226	0.003	1.603	0.002	1.475	0.009	1.409	0.005	1.388	0.002
1714	5.834	3.035	0.001	2.571	0.001	1.666	0.001	1.332	0.001	1.275	0.003	1.226	0.003	1.602	0.002	1.475	0.009	1.409	0.005	1.387	0.002
1712	5.841	3.034	0.001	2.571	0.001	1.666	0.001	1.331	0.001	1.274	0.003	1.225	0.003	1.602	0.002	1.474	0.009	1.408	0.005	1.387	0.002
1710	5.848	3.034	0.001	2.571	0.001	1.666	0.001	1.331	0.001	1.274	0.003	1.224	0.003	1.602	0.002	1.474	0.009	1.408	0.005	1.386	0.002
1708	5.855	3.034	0.001	2.571	0.001	1.665	0.001	1.330	0.001	1.273	0.003	1.224	0.003	1.601	0.002	1.473	0.009	1.407	0.005	1.386	0.002
1706	5.862	3.034	0.001	2.570	0.002	1.665	0.001	1.330	0.001	1.273	0.003	1.223	0.003	1.601	0.002	1.473	0.009	1.407	0.005	1.385	0.002
1704	5.869	3.034	0.001	2.570	0.002	1.664	0.001	1.329	0.001	1.272	0.003	1.223	0.003	1.600	0.002	1.472	0.009	1.406	0.005	1.385	0.002
1702	5.875	3.034	0.001	2.570	0.002	1.664	0.001	1.329	0.001	1.271	0.003	1.222	0.003	1.600	0.002	1.472	0.009	1.406	0.005	1.384	0.002
1700	5.882	3.033	0.001	2.570	0.002	1.663	0.002	1.328	0.001	1.271	0.003	1.221	0.003	1.599	0.002	1.471	0.009	1.405	0.005	1.384	0.002
1698	5.889	3.033	0.001	2.569	0.002	1.663	0.002	1.327	0.001	1.270	0.003	1.221	0.003	1.599	0.002	1.471	0.009	1.405	0.005	1.383	0.002
1696	5.896	3.033	0.001	2.569	0.002	1.662	0.002	1.327	0.001	1.270	0.003	1.220	0.003	1.598	0.002	1.470	0.009	1.404	0.005	1.383	0.002
1694	5.903	3.033	0.001	2.569	0.002	1.662	0.002	1.326	0.001	1.269	0.003	1.220	0.003	1.598	0.002	1.470	0.010	1.404	0.005	1.382	0.002
1692	5.910	3.033	0.001	2.569	0.002	1.661	0.002	1.326	0.001	1.268	0.003	1.219	0.003	1.597	0.002	1.469	0.010	1.403	0.006	1.382	0.002
1690	5.917	3.033	0.001	2.568	0.002	1.661	0.002	1.325	0.001	1.268	0.003	1.219	0.004	1.597	0.002	1.469	0.010	1.403	0.006	1.381	0.002
1688	5.924	3.032	0.001	2.568	0.002	1.660	0.002	1.324	0.001	1.267	0.003	1.218	0.004	1.596	0.002	1.468	0.010	1.402	0.006	1.381	0.002
1686	5.931	3.032	0.001	2.568	0.002	1.660	0.002	1.324	0.001	1.266	0.003	1.217	0.004	1.596	0.003	1.468	0.010	1.401	0.006	1.380	0.002
1684	5.938	3.032	0.001	2.568	0.002	1.660	0.002	1.323	0.001	1.266	0.003	1.217	0.004	1.595	0.003	1.467	0.010	1.401	0.006	1.379	0.002

Wavenumber (cm^{-1})	Wavelength (µm)	n10	k10	n20	k20	n30	k30	n40	k40	n80	k80	n120	k120	n31	k31	n32	k32	n33	k33	n34	k34
1682	5.945	3.032	0.001	2.567	0.002	1.659	0.002	1.322	0.001	1.265	0.003	1.216	0.004	1.595	0.003	1.467	0.010	1.400	0.006	1.379	0.002
1680	5.952	3.032	0.001	2.567	0.002	1.659	0.002	1.322	0.001	1.264	0.003	1.215	0.004	1.594	0.003	1.466	0.010	1.400	0.006	1.378	0.002
1678	5.959	3.032	0.001	2.567	0.002	1.658	0.002	1.321	0.001	1.264	0.003	1.215	0.004	1.594	0.003	1.466	0.010	1.399	0.006	1.378	0.002
1676	5.967	3.031	0.001	2.567	0.002	1.658	0.002	1.321	0.001	1.263	0.003	1.214	0.004	1.593	0.003	1.465	0.010	1.399	0.006	1.377	0.002
1674	5.974	3.031	0.001	2.566	0.002	1.657	0.002	1.320	0.001	1.262	0.003	1.214	0.004	1.593	0.003	1.465	0.010	1.398	0.006	1.377	0.002
1672	5.981	3.031	0.002	2.566	0.002	1.657	0.002	1.319	0.001	1.262	0.003	1.213	0.004	1.592	0.003	1.464	0.011	1.398	0.006	1.376	0.002
1670	5.988	3.031	0.002	2.566	0.002	1.656	0.002	1.319	0.001	1.261	0.003	1.212	0.004	1.592	0.003	1.464	0.011	1.397	0.006	1.375	0.002
1668	5.995	3.031	0.002	2.566	0.002	1.656	0.002	1.318	0.001	1.260	0.003	1.212	0.004	1.591	0.003	1.463	0.011	1.396	0.006	1.375	0.002
1666	6.002	3.031	0.002	2.565	0.002	1.655	0.002	1.317	0.001	1.260	0.003	1.211	0.004	1.591	0.003	1.463	0.011	1.396	0.006	1.374	0.002
1664	6.010	3.030	0.002	2.565	0.002	1.655	0.002	1.317	0.001	1.259	0.003	1.210	0.004	1.590	0.003	1.462	0.011	1.395	0.006	1.374	0.002
1662	6.017	3.030	0.002	2.565	0.002	1.654	0.002	1.316	0.002	1.258	0.003	1.210	0.004	1.589	0.003	1.462	0.011	1.395	0.006	1.373	0.002
1660	6.024	3.030	0.002	2.565	0.002	1.654	0.002	1.315	0.002	1.258	0.003	1.209	0.004	1.589	0.003	1.461	0.011	1.394	0.007	1.372	0.002
1658	6.031	3.030	0.002	2.564	0.002	1.653	0.002	1.315	0.002	1.257	0.003	1.208	0.004	1.588	0.003	1.460	0.011	1.394	0.007	1.372	0.002
1656	6.039	3.030	0.002	2.564	0.002	1.652	0.002	1.314	0.002	1.256	0.003	1.208	0.004	1.588	0.003	1.460	0.011	1.393	0.007	1.371	0.002
1654	6.046	3.029	0.002	2.564	0.002	1.652	0.002	1.313	0.002	1.255	0.003	1.207	0.004	1.587	0.003	1.459	0.011	1.392	0.007	1.371	0.002
1652	6.053	3.029	0.002	2.564	0.002	1.651	0.002	1.312	0.002	1.255	0.003	1.206	0.004	1.587	0.003	1.459	0.011	1.392	0.007	1.370	0.002
1650	6.061	3.029	0.002	2.563	0.002	1.651	0.002	1.312	0.002	1.254	0.003	1.206	0.004	1.586	0.003	1.458	0.012	1.391	0.007	1.369	0.002
1648	6.068	3.029	0.002	2.563	0.002	1.650	0.002	1.311	0.002	1.253	0.003	1.205	0.004	1.586	0.003	1.458	0.012	1.390	0.007	1.369	0.002
1646	6.075	3.029	0.002	2.563	0.002	1.650	0.002	1.310	0.002	1.252	0.003	1.204	0.004	1.585	0.003	1.457	0.012	1.390	0.007	1.368	0.002
1644	6.083	3.029	0.002	2.563	0.002	1.649	0.002	1.310	0.002	1.252	0.003	1.203	0.004	1.584	0.003	1.457	0.012	1.389	0.007	1.367	0.002
1642	6.090	3.028	0.002	2.562	0.002	1.649	0.002	1.309	0.002	1.251	0.003	1.203	0.004	1.584	0.003	1.456	0.012	1.389	0.007	1.367	0.003
1640	6.098	3.028	0.002	2.562	0.002	1.648	0.002	1.308	0.002	1.250	0.003	1.202	0.004	1.583	0.003	1.455	0.012	1.388	0.007	1.366	0.003
1638	6.105	3.028	0.002	2.562	0.002	1.648	0.002	1.307	0.002	1.249	0.003	1.201	0.005	1.583	0.003	1.455	0.012	1.387	0.007	1.366	0.003
1636	6.112	3.028	0.002	2.561	0.002	1.647	0.002	1.307	0.002	1.249	0.003	1.201	0.005	1.582	0.003	1.454	0.012	1.387	0.007	1.365	0.003
1634	6.120	3.028	0.002	2.561	0.002	1.646	0.002	1.306	0.002	1.248	0.003	1.200	0.005	1.581	0.003	1.454	0.012	1.386	0.007	1.364	0.003
1632	6.127	3.027	0.002	2.561	0.003	1.646	0.002	1.305	0.002	1.247	0.003	1.199	0.005	1.581	0.003	1.453	0.012	1.385	0.008	1.364	0.003
1630	6.135	3.027	0.002	2.561	0.003	1.645	0.002	1.304	0.002	1.246	0.003	1.198	0.005	1.580	0.004	1.452	0.013	1.385	0.008	1.363	0.003
1628	6.143	3.027	0.002	2.560	0.003	1.645	0.002	1.304	0.002	1.245	0.003	1.198	0.005	1.580	0.004	1.452	0.013	1.384	0.008	1.362	0.003
1626	6.150	3.027	0.002	2.560	0.003	1.644	0.003	1.303	0.002	1.245	0.003	1.197	0.005	1.579	0.004	1.451	0.013	1.383	0.008	1.362	0.003
1624	6.158	3.027	0.002	2.560	0.003	1.644	0.003	1.302	0.002	1.244	0.004	1.196	0.005	1.578	0.004	1.451	0.013	1.383	0.008	1.361	0.003
1622	6.165	3.026	0.002	2.559	0.003	1.643	0.003	1.301	0.002	1.243	0.004	1.195	0.005	1.578	0.004	1.450	0.013	1.382	0.008	1.360	0.003
1620	6.173	3.026	0.002	2.559	0.003	1.642	0.003	1.301	0.002	1.242	0.004	1.195	0.005	1.577	0.004	1.449	0.013	1.381	0.008	1.359	0.003

Wavenumber (cm^{-1})	Wavelength (μm)	n10	k10	n20	k20	n30	k30	n40	k40	n80	k80	n120	k120	n31	k31	n32	k32	n33	k33	n34	k34
1618	6.180	3.026	0.002	2.559	0.003	1.642	0.003	1.300	0.002	1.241	0.004	1.194	0.005	1.577	0.004	1.449	0.013	1.381	0.008	1.359	0.003
1616	6.188	3.026	0.002	2.559	0.003	1.641	0.003	1.299	0.002	1.241	0.004	1.193	0.005	1.576	0.004	1.448	0.013	1.380	0.008	1.358	0.003
1614	6.196	3.026	0.002	2.558	0.003	1.641	0.003	1.298	0.002	1.240	0.004	1.192	0.005	1.575	0.004	1.448	0.013	1.379	0.008	1.357	0.003
1612	6.203	3.025	0.002	2.558	0.003	1.640	0.003	1.297	0.002	1.239	0.004	1.191	0.005	1.575	0.004	1.447	0.014	1.379	0.008	1.357	0.003
1610	6.211	3.025	0.002	2.558	0.003	1.639	0.003	1.297	0.002	1.238	0.004	1.191	0.005	1.574	0.004	1.446	0.014	1.378	0.008	1.356	0.003
1608	6.219	3.025	0.002	2.557	0.003	1.639	0.003	1.296	0.002	1.237	0.004	1.190	0.005	1.573	0.004	1.446	0.014	1.377	0.008	1.355	0.003
1606	6.227	3.025	0.002	2.557	0.003	1.638	0.003	1.295	0.002	1.236	0.004	1.189	0.005	1.573	0.004	1.445	0.014	1.377	0.009	1.354	0.003
1604	6.234	3.024	0.002	2.557	0.003	1.638	0.003	1.294	0.002	1.235	0.004	1.188	0.005	1.572	0.004	1.444	0.014	1.376	0.009	1.354	0.003
1602	6.242	3.024	0.002	2.556	0.003	1.637	0.003	1.293	0.002	1.235	0.004	1.187	0.005	1.571	0.004	1.444	0.014	1.375	0.009	1.353	0.003
1600	6.250	3.024	0.002	2.556	0.003	1.636	0.003	1.292	0.002	1.234	0.004	1.187	0.005	1.571	0.004	1.443	0.014	1.375	0.009	1.352	0.003
1598	6.258	3.024	0.002	2.556	0.003	1.636	0.003	1.292	0.002	1.233	0.004	1.186	0.006	1.570	0.004	1.442	0.014	1.374	0.009	1.351	0.003
1596	6.266	3.024	0.003	2.555	0.003	1.635	0.003	1.291	0.002	1.232	0.004	1.185	0.006	1.569	0.004	1.442	0.015	1.373	0.009	1.351	0.003
1594	6.274	3.023	0.003	2.555	0.003	1.634	0.003	1.290	0.002	1.231	0.004	1.184	0.006	1.569	0.004	1.441	0.015	1.372	0.009	1.350	0.003
1592	6.281	3.023	0.003	2.555	0.003	1.634	0.003	1.289	0.002	1.230	0.004	1.183	0.006	1.568	0.004	1.440	0.015	1.372	0.009	1.349	0.003
1590	6.289	3.023	0.003	2.555	0.003	1.633	0.003	1.288	0.002	1.229	0.004	1.182	0.006	1.567	0.004	1.440	0.015	1.371	0.009	1.348	0.004
1588	6.297	3.023	0.003	2.554	0.003	1.632	0.003	1.287	0.002	1.228	0.004	1.181	0.006	1.567	0.005	1.439	0.015	1.370	0.009	1.348	0.004
1586	6.305	3.023	0.003	2.554	0.003	1.632	0.003	1.286	0.002	1.227	0.004	1.181	0.006	1.566	0.005	1.438	0.015	1.369	0.009	1.347	0.004
1584	6.313	3.022	0.003	2.554	0.003	1.631	0.003	1.285	0.002	1.226	0.004	1.180	0.006	1.565	0.005	1.438	0.015	1.369	0.010	1.346	0.004
1582	6.321	3.022	0.003	2.553	0.004	1.630	0.003	1.284	0.002	1.225	0.004	1.179	0.006	1.565	0.005	1.437	0.015	1.368	0.010	1.345	0.004
1580	6.329	3.022	0.003	2.553	0.004	1.630	0.003	1.283	0.002	1.224	0.004	1.178	0.006	1.564	0.005	1.436	0.016	1.367	0.010	1.344	0.004
1578	6.337	3.022	0.003	2.553	0.004	1.629	0.003	1.283	0.002	1.223	0.004	1.177	0.006	1.563	0.005	1.436	0.016	1.366	0.010	1.344	0.004
1576	6.345	3.021	0.003	2.552	0.004	1.628	0.004	1.282	0.002	1.222	0.004	1.176	0.006	1.562	0.005	1.435	0.016	1.365	0.010	1.343	0.004
1574	6.353	3.021	0.003	2.552	0.004	1.628	0.004	1.281	0.002	1.221	0.004	1.175	0.006	1.562	0.005	1.434	0.016	1.365	0.010	1.342	0.004
1572	6.361	3.021	0.003	2.552	0.004	1.627	0.004	1.280	0.002	1.220	0.004	1.174	0.006	1.561	0.005	1.433	0.016	1.364	0.010	1.341	0.004
1570	6.369	3.021	0.003	2.551	0.004	1.626	0.004	1.279	0.002	1.219	0.004	1.173	0.006	1.560	0.005	1.433	0.016	1.363	0.010	1.340	0.004
1568	6.378	3.020	0.003	2.551	0.004	1.626	0.004	1.278	0.002	1.218	0.004	1.172	0.006	1.559	0.005	1.432	0.016	1.362	0.010	1.339	0.004
1566	6.386	3.020	0.003	2.551	0.004	1.625	0.004	1.277	0.002	1.217	0.004	1.171	0.006	1.559	0.005	1.431	0.016	1.361	0.010	1.339	0.004
1564	6.394	3.020	0.003	2.550	0.004	1.624	0.004	1.276	0.002	1.216	0.004	1.170	0.007	1.558	0.005	1.430	0.017	1.361	0.011	1.338	0.004
1562	6.402	3.020	0.003	2.550	0.004	1.623	0.004	1.275	0.002	1.215	0.004	1.169	0.007	1.557	0.005	1.430	0.017	1.360	0.011	1.337	0.004
1560	6.410	3.019	0.003	2.549	0.004	1.623	0.004	1.274	0.002	1.214	0.004	1.168	0.007	1.556	0.005	1.429	0.017	1.359	0.011	1.336	0.004
1558	6.418	3.019	0.003	2.549	0.004	1.622	0.004	1.273	0.002	1.213	0.005	1.167	0.007	1.556	0.005	1.428	0.017	1.358	0.011	1.335	0.004
1556	6.427	3.019	0.003	2.549	0.004	1.621	0.004	1.272	0.002	1.212	0.005	1.166	0.007	1.555	0.005	1.427	0.017	1.357	0.011	1.334	0.004

Wavenumber (cm^{-1})	Wavelength (μm)	n10	k10	n20	k20	n30	k30	n40	k40	n80	k80	n120	k120	n31	k31	n32	k32	n33	k33	n34	k34
1554	6.435	3.019	0.003	2.548	0.004	1.620	0.004	1.271	0.002	1.211	0.005	1.165	0.007	1.554	0.006	1.427	0.017	1.356	0.011	1.333	0.004
1552	6.443	3.018	0.003	2.548	0.004	1.620	0.004	1.270	0.002	1.210	0.005	1.164	0.007	1.553	0.006	1.426	0.017	1.356	0.011	1.332	0.005
1550	6.452	3.018	0.003	2.548	0.004	1.619	0.004	1.269	0.002	1.209	0.005	1.163	0.007	1.552	0.006	1.425	0.018	1.355	0.011	1.332	0.005
1548	6.460	3.018	0.003	2.547	0.004	1.618	0.004	1.268	0.002	1.208	0.005	1.162	0.007	1.552	0.006	1.424	0.018	1.354	0.011	1.331	0.005
1546	6.468	3.018	0.003	2.547	0.004	1.617	0.004	1.266	0.002	1.206	0.005	1.161	0.007	1.551	0.006	1.424	0.018	1.353	0.011	1.330	0.005
1544	6.477	3.017	0.003	2.547	0.005	1.617	0.004	1.265	0.002	1.205	0.005	1.160	0.007	1.550	0.006	1.423	0.018	1.352	0.012	1.329	0.005
1542	6.485	3.017	0.004	2.546	0.005	1.616	0.004	1.264	0.002	1.204	0.005	1.159	0.007	1.549	0.006	1.422	0.018	1.351	0.012	1.328	0.005
1540	6.494	3.017	0.004	2.546	0.005	1.615	0.004	1.263	0.002	1.203	0.005	1.158	0.007	1.548	0.006	1.421	0.018	1.350	0.012	1.327	0.005
1538	6.502	3.017	0.004	2.545	0.005	1.614	0.005	1.262	0.002	1.202	0.005	1.157	0.007	1.547	0.006	1.420	0.019	1.349	0.012	1.326	0.005
1536	6.510	3.016	0.004	2.545	0.005	1.613	0.005	1.261	0.002	1.201	0.005	1.156	0.007	1.547	0.006	1.419	0.019	1.348	0.012	1.325	0.005
1534	6.519	3.016	0.004	2.545	0.005	1.613	0.005	1.260	0.002	1.200	0.005	1.155	0.008	1.546	0.006	1.419	0.019	1.348	0.012	1.324	0.005
1532	6.527	3.016	0.004	2.544	0.005	1.612	0.005	1.259	0.002	1.198	0.005	1.154	0.008	1.545	0.006	1.418	0.019	1.347	0.012	1.323	0.005
1530	6.536	3.016	0.004	2.544	0.005	1.611	0.005	1.257	0.002	1.197	0.005	1.153	0.008	1.544	0.006	1.417	0.019	1.346	0.012	1.322	0.005
1528	6.545	3.015	0.004	2.543	0.005	1.610	0.005	1.256	0.003	1.196	0.005	1.152	0.008	1.543	0.006	1.416	0.019	1.345	0.013	1.321	0.005
1526	6.553	3.015	0.004	2.543	0.005	1.609	0.005	1.255	0.003	1.195	0.005	1.151	0.008	1.542	0.007	1.415	0.019	1.344	0.013	1.320	0.005
1524	6.562	3.015	0.004	2.543	0.005	1.608	0.005	1.254	0.003	1.193	0.005	1.149	0.008	1.541	0.007	1.414	0.020	1.343	0.013	1.319	0.005
1522	6.570	3.015	0.004	2.542	0.005	1.607	0.005	1.253	0.003	1.192	0.005	1.148	0.008	1.540	0.007	1.414	0.020	1.342	0.013	1.318	0.005
1520	6.579	3.014	0.004	2.542	0.005	1.607	0.005	1.252	0.003	1.191	0.005	1.147	0.008	1.540	0.007	1.413	0.020	1.341	0.013	1.317	0.006
1518	6.588	3.014	0.004	2.541	0.005	1.606	0.005	1.250	0.003	1.190	0.005	1.146	0.008	1.539	0.007	1.412	0.020	1.340	0.013	1.316	0.006
1516	6.596	3.014	0.004	2.541	0.005	1.605	0.005	1.249	0.003	1.188	0.005	1.145	0.008	1.538	0.007	1.411	0.020	1.339	0.013	1.315	0.006
1514	6.605	3.013	0.004	2.541	0.005	1.604	0.005	1.248	0.003	1.187	0.005	1.144	0.008	1.537	0.007	1.410	0.020	1.338	0.013	1.314	0.006
1512	6.614	3.013	0.004	2.540	0.006	1.603	0.005	1.247	0.003	1.186	0.006	1.142	0.008	1.536	0.007	1.409	0.021	1.337	0.014	1.313	0.006
1510	6.623	3.013	0.004	2.540	0.006	1.602	0.005	1.245	0.003	1.184	0.006	1.141	0.008	1.535	0.007	1.408	0.021	1.336	0.014	1.312	0.006
1508	6.631	3.013	0.004	2.539	0.006	1.601	0.006	1.244	0.003	1.183	0.006	1.140	0.009	1.534	0.007	1.407	0.021	1.335	0.014	1.311	0.006
1506	6.640	3.012	0.004	2.539	0.006	1.600	0.006	1.243	0.003	1.182	0.006	1.139	0.009	1.533	0.007	1.406	0.021	1.334	0.014	1.309	0.006
1504	6.649	3.012	0.004	2.539	0.006	1.599	0.006	1.241	0.003	1.180	0.006	1.137	0.009	1.532	0.007	1.405	0.021	1.333	0.014	1.308	0.006
1502	6.658	3.012	0.005	2.538	0.006	1.599	0.006	1.240	0.003	1.179	0.006	1.136	0.009	1.531	0.008	1.404	0.021	1.332	0.014	1.307	0.006
1500	6.667	3.011	0.005	2.538	0.006	1.598	0.006	1.239	0.003	1.177	0.006	1.135	0.009	1.530	0.008	1.404	0.022	1.331	0.014	1.306	0.006
1498	6.676	3.011	0.005	2.537	0.006	1.597	0.006	1.237	0.003	1.176	0.006	1.134	0.009	1.529	0.008	1.403	0.022	1.330	0.015	1.305	0.006
1496	6.684	3.011	0.005	2.537	0.006	1.596	0.006	1.236	0.003	1.174	0.006	1.132	0.009	1.528	0.008	1.402	0.022	1.328	0.015	1.304	0.006
1494	6.693	3.011	0.005	2.536	0.006	1.595	0.006	1.235	0.003	1.173	0.006	1.131	0.009	1.527	0.008	1.401	0.022	1.327	0.015	1.303	0.007
1492	6.702	3.010	0.005	2.536	0.006	1.594	0.006	1.233	0.003	1.172	0.006	1.130	0.009	1.526	0.008	1.400	0.022	1.326	0.015	1.301	0.007

Wavenumber (cm^{-1})	Wavelength (µm)	n10	k10	n20	k20	n30	k30	n40	k40	n80	k80	n120	k120	n31	k31	n32	k32	n33	k33	n34	k34
1490	6.711	3.010	0.005	2.535	0.006	1.593	0.006	1.232	0.003	1.170	0.006	1.128	0.009	1.525	0.008	1.399	0.023	1.325	0.015	1.300	0.007
1488	6.720	3.010	0.005	2.535	0.006	1.592	0.006	1.230	0.003	1.169	0.006	1.127	0.009	1.524	0.008	1.398	0.023	1.324	0.015	1.299	0.007
1486	6.729	3.009	0.005	2.535	0.006	1.591	0.006	1.229	0.003	1.167	0.006	1.125	0.010	1.523	0.008	1.397	0.023	1.323	0.015	1.298	0.007
1484	6.739	3.009	0.005	2.534	0.007	1.590	0.006	1.227	0.003	1.165	0.006	1.124	0.010	1.522	0.008	1.396	0.023	1.322	0.016	1.297	0.007
1482	6.748	3.009	0.005	2.534	0.007	1.589	0.007	1.226	0.003	1.164	0.006	1.123	0.010	1.521	0.008	1.395	0.023	1.321	0.016	1.295	0.007
1480	6.757	3.008	0.005	2.533	0.007	1.588	0.007	1.224	0.003	1.162	0.006	1.121	0.010	1.520	0.009	1.394	0.023	1.319	0.016	1.294	0.007
1478	6.766	3.008	0.005	2.533	0.007	1.587	0.007	1.223	0.003	1.161	0.007	1.120	0.010	1.519	0.009	1.393	0.024	1.318	0.016	1.293	0.007
1476	6.775	3.008	0.005	2.532	0.007	1.586	0.007	1.221	0.003	1.159	0.007	1.118	0.010	1.517	0.009	1.391	0.024	1.317	0.016	1.292	0.007
1474	6.784	3.007	0.005	2.532	0.007	1.585	0.007	1.220	0.003	1.158	0.007	1.117	0.010	1.516	0.009	1.390	0.024	1.316	0.016	1.290	0.007
1472	6.793	3.007	0.005	2.531	0.007	1.583	0.007	1.218	0.003	1.156	0.007	1.115	0.010	1.515	0.009	1.389	0.024	1.315	0.017	1.289	0.007
1470	6.803	3.007	0.006	2.531	0.007	1.582	0.007	1.217	0.003	1.154	0.007	1.114	0.010	1.514	0.009	1.388	0.024	1.313	0.017	1.288	0.008
1468	6.812	3.006	0.006	2.530	0.007	1.581	0.007	1.215	0.003	1.153	0.007	1.112	0.010	1.513	0.009	1.387	0.025	1.312	0.017	1.286	0.008
1466	6.821	3.006	0.006	2.530	0.007	1.580	0.007	1.214	0.003	1.151	0.007	1.111	0.010	1.512	0.009	1.386	0.025	1.311	0.017	1.285	0.008
1464	6.831	3.006	0.006	2.529	0.007	1.579	0.007	1.212	0.003	1.149	0.007	1.109	0.011	1.511	0.009	1.385	0.025	1.310	0.017	1.284	0.008
1462	6.840	3.006	0.006	2.529	0.008	1.578	0.007	1.210	0.004	1.147	0.007	1.108	0.011	1.509	0.010	1.384	0.025	1.308	0.017	1.282	0.008
1460	6.849	3.005	0.006	2.528	0.008	1.577	0.008	1.209	0.004	1.146	0.007	1.106	0.011	1.508	0.010	1.383	0.025	1.307	0.018	1.281	0.008
1458	6.859	3.005	0.006	2.528	0.008	1.576	0.008	1.207	0.004	1.144	0.007	1.105	0.011	1.507	0.010	1.382	0.026	1.306	0.018	1.279	0.008
1456	6.868	3.004	0.006	2.527	0.008	1.575	0.008	1.205	0.004	1.142	0.007	1.103	0.011	1.506	0.010	1.380	0.026	1.305	0.018	1.278	0.008
1454	6.878	3.004	0.006	2.527	0.008	1.573	0.008	1.203	0.004	1.140	0.007	1.101	0.011	1.505	0.010	1.379	0.026	1.303	0.018	1.277	0.008
1452	6.887	3.004	0.006	2.526	0.008	1.572	0.008	1.202	0.004	1.138	0.007	1.100	0.011	1.503	0.010	1.378	0.026	1.302	0.018	1.275	0.008
1450	6.897	3.003	0.006	2.526	0.008	1.571	0.008	1.200	0.004	1.136	0.008	1.098	0.011	1.502	0.010	1.377	0.026	1.301	0.018	1.274	0.009
1448	6.906	3.003	0.006	2.525	0.008	1.570	0.008	1.198	0.004	1.135	0.008	1.096	0.011	1.501	0.010	1.376	0.027	1.299	0.019	1.272	0.009
1446	6.916	3.003	0.006	2.525	0.008	1.569	0.008	1.196	0.004	1.133	0.008	1.095	0.012	1.500	0.011	1.374	0.027	1.298	0.019	1.271	0.009
1444	6.925	3.002	0.006	2.524	0.008	1.567	0.008	1.194	0.004	1.131	0.008	1.093	0.012	1.498	0.011	1.373	0.027	1.296	0.019	1.269	0.009
1442	6.935	3.002	0.007	2.524	0.008	1.566	0.008	1.193	0.004	1.129	0.008	1.091	0.012	1.497	0.011	1.372	0.027	1.295	0.019	1.268	0.009
1440	6.944	3.002	0.007	2.523	0.009	1.565	0.009	1.191	0.004	1.127	0.008	1.089	0.012	1.496	0.011	1.371	0.028	1.294	0.019	1.266	0.009
1438	6.954	3.001	0.007	2.522	0.009	1.564	0.009	1.189	0.004	1.125	0.008	1.088	0.012	1.494	0.011	1.370	0.028	1.292	0.020	1.265	0.009
1436	6.964	3.001	0.007	2.522	0.009	1.562	0.009	1.187	0.004	1.123	0.008	1.086	0.012	1.493	0.011	1.368	0.028	1.291	0.020	1.263	0.009
1434	6.974	3.001	0.007	2.521	0.009	1.561	0.009	1.185	0.004	1.121	0.008	1.084	0.012	1.492	0.011	1.367	0.028	1.289	0.020	1.261	0.009
1432	6.983	3.000	0.007	2.521	0.009	1.560	0.009	1.183	0.004	1.118	0.008	1.082	0.012	1.490	0.011	1.366	0.028	1.288	0.020	1.260	0.010
1430	6.993	3.000	0.007	2.520	0.009	1.558	0.009	1.181	0.004	1.116	0.008	1.080	0.013	1.489	0.012	1.364	0.029	1.286	0.020	1.258	0.010
1428	7.003	2.999	0.007	2.520	0.009	1.557	0.009	1.179	0.004	1.114	0.009	1.078	0.013	1.487	0.012	1.363	0.029	1.285	0.021	1.257	0.010

Wavenumber (cm^{-1})	Wavelength (μm)	n10	k10	n20	k20	n30	k30	n40	k40	n80	k80	n120	k120	n31	k31	n32	k32	n33	k33	n34	k34
1426	7.013	2.999	0.007	2.519	0.009	1.556	0.009	1.177	0.004	1.112	0.009	1.076	0.013	1.486	0.012	1.362	0.029	1.283	0.021	1.255	0.010
1424	7.022	2.999	0.007	2.518	0.009	1.554	0.009	1.175	0.004	1.110	0.009	1.074	0.013	1.485	0.012	1.360	0.029	1.282	0.021	1.253	0.010
1422	7.032	2.998	0.007	2.518	0.010	1.553	0.010	1.173	0.005	1.107	0.009	1.072	0.013	1.483	0.012	1.359	0.030	1.280	0.021	1.251	0.010
1420	7.042	2.998	0.007	2.517	0.010	1.552	0.010	1.170	0.005	1.105	0.009	1.070	0.013	1.482	0.012	1.358	0.030	1.278	0.021	1.250	0.010
1418	7.052	2.998	0.007	2.517	0.010	1.550	0.010	1.168	0.005	1.103	0.009	1.068	0.013	1.480	0.012	1.356	0.030	1.277	0.022	1.248	0.010
1416	7.062	2.997	0.008	2.516	0.010	1.549	0.010	1.166	0.005	1.100	0.009	1.066	0.013	1.479	0.013	1.355	0.030	1.275	0.022	1.246	0.011
1414	7.072	2.997	0.008	2.515	0.010	1.547	0.010	1.164	0.005	1.098	0.009	1.064	0.014	1.477	0.013	1.353	0.031	1.274	0.022	1.244	0.011
1412	7.082	2.996	0.008	2.515	0.010	1.546	0.010	1.161	0.005	1.096	0.009	1.062	0.014	1.476	0.013	1.352	0.031	1.272	0.022	1.243	0.011
1410	7.092	2.996	0.008	2.514	0.010	1.544	0.010	1.159	0.005	1.093	0.010	1.060	0.014	1.474	0.013	1.350	0.031	1.270	0.022	1.241	0.011
1408	7.102	2.995	0.008	2.514	0.010	1.543	0.010	1.157	0.005	1.091	0.010	1.058	0.014	1.472	0.013	1.349	0.031	1.268	0.023	1.239	0.011
1406	7.112	2.995	0.008	2.513	0.010	1.541	0.011	1.154	0.005	1.088	0.010	1.056	0.014	1.471	0.013	1.347	0.032	1.267	0.023	1.237	0.011
1404	7.123	2.995	0.008	2.512	0.011	1.540	0.011	1.152	0.005	1.086	0.010	1.053	0.014	1.469	0.014	1.346	0.032	1.265	0.023	1.235	0.011
1402	7.133	2.994	0.008	2.512	0.011	1.538	0.011	1.150	0.005	1.083	0.010	1.051	0.015	1.468	0.014	1.344	0.032	1.263	0.023	1.233	0.012
1400	7.143	2.994	0.008	2.511	0.011	1.537	0.011	1.147	0.005	1.080	0.010	1.049	0.015	1.466	0.014	1.343	0.032	1.261	0.024	1.231	0.012
1398	7.153	2.993	0.008	2.510	0.011	1.535	0.011	1.145	0.005	1.078	0.010	1.046	0.015	1.464	0.014	1.341	0.033	1.260	0.024	1.229	0.012
1396	7.163	2.993	0.009	2.510	0.011	1.533	0.011	1.142	0.005	1.075	0.010	1.044	0.015	1.463	0.014	1.340	0.033	1.258	0.024	1.227	0.012
1394	7.174	2.993	0.009	2.509	0.011	1.532	0.011	1.139	0.005	1.072	0.011	1.042	0.015	1.461	0.014	1.338	0.033	1.256	0.024	1.225	0.012
1392	7.184	2.992	0.009	2.508	0.011	1.530	0.012	1.137	0.006	1.069	0.011	1.039	0.015	1.459	0.015	1.336	0.033	1.254	0.025	1.223	0.012
1390	7.194	2.992	0.009	2.508	0.011	1.529	0.012	1.134	0.006	1.066	0.011	1.037	0.015	1.457	0.015	1.335	0.034	1.252	0.025	1.221	0.012
1388	7.205	2.991	0.009	2.507	0.012	1.527	0.012	1.131	0.006	1.063	0.011	1.034	0.016	1.456	0.015	1.333	0.034	1.250	0.025	1.219	0.013
1386	7.215	2.991	0.009	2.506	0.012	1.525	0.012	1.128	0.006	1.060	0.011	1.032	0.016	1.454	0.015	1.331	0.034	1.248	0.025	1.216	0.013
1384	7.225	2.990	0.009	2.506	0.012	1.523	0.012	1.126	0.006	1.057	0.011	1.029	0.016	1.452	0.015	1.330	0.035	1.246	0.026	1.214	0.013
1382	7.236	2.990	0.009	2.505	0.012	1.522	0.012	1.123	0.006	1.054	0.012	1.026	0.016	1.450	0.015	1.328	0.035	1.244	0.026	1.212	0.013
1380	7.246	2.989	0.009	2.504	0.012	1.520	0.012	1.120	0.006	1.051	0.012	1.024	0.016	1.448	0.016	1.326	0.035	1.242	0.026	1.210	0.013
1378	7.257	2.989	0.009	2.503	0.012	1.518	0.013	1.117	0.006	1.048	0.012	1.021	0.017	1.446	0.016	1.324	0.035	1.240	0.026	1.207	0.013
1376	7.267	2.988	0.010	2.503	0.012	1.516	0.013	1.114	0.006	1.045	0.012	1.018	0.017	1.444	0.016	1.322	0.036	1.238	0.027	1.205	0.014
1374	7.278	2.988	0.010	2.502	0.013	1.514	0.013	1.111	0.006	1.041	0.012	1.015	0.017	1.442	0.016	1.321	0.036	1.236	0.027	1.203	0.014
1372	7.289	2.987	0.010	2.501	0.013	1.512	0.013	1.108	0.007	1.038	0.012	1.012	0.017	1.440	0.016	1.319	0.036	1.233	0.027	1.200	0.014
1370	7.299	2.987	0.010	2.500	0.013	1.511	0.013	1.104	0.007	1.035	0.013	1.009	0.017	1.438	0.017	1.317	0.037	1.231	0.028	1.198	0.014
1368	7.310	2.987	0.010	2.500	0.013	1.509	0.013	1.101	0.007	1.031	0.013	1.007	0.017	1.436	0.017	1.315	0.037	1.229	0.028	1.195	0.014
1366	7.321	2.986	0.010	2.499	0.013	1.507	0.014	1.098	0.007	1.028	0.013	1.004	0.018	1.434	0.017	1.313	0.037	1.227	0.028	1.193	0.015
1364	7.331	2.986	0.010	2.498	0.013	1.505	0.014	1.095	0.007	1.024	0.013	1.000	0.018	1.432	0.017	1.311	0.037	1.224	0.028	1.190	0.015

Wavenumber (cm^{-1})	Wavelength (µm)	n10	k10	n20	k20	n30	k30	n40	k40	n80	k80	n120	k120	n31	k31	n32	k32	n33	k33	n34	k34
1362	7.342	2.985	0.010	2.497	0.013	1.503	0.014	1.091	0.007	1.020	0.014	0.997	0.018	1.430	0.018	1.309	0.038	1.222	0.029	1.187	0.015
1360	7.353	2.985	0.010	2.496	0.014	1.501	0.014	1.088	0.007	1.016	0.014	0.994	0.018	1.428	0.018	1.307	0.038	1.220	0.029	1.185	0.015
1358	7.364	2.984	0.011	2.496	0.014	1.498	0.014	1.084	0.007	1.013	0.014	0.991	0.019	1.426	0.018	1.305	0.038	1.217	0.029	1.182	0.015
1356	7.375	2.983	0.011	2.495	0.014	1.496	0.015	1.080	0.008	1.009	0.014	0.988	0.019	1.423	0.018	1.303	0.039	1.215	0.030	1.179	0.016
1354	7.386	2.983	0.011	2.494	0.014	1.494	0.015	1.077	0.008	1.005	0.015	0.984	0.019	1.421	0.018	1.301	0.039	1.212	0.030	1.176	0.016
1352	7.396	2.982	0.011	2.493	0.014	1.492	0.015	1.073	0.008	1.001	0.015	0.981	0.019	1.419	0.019	1.299	0.039	1.210	0.030	1.174	0.016
1350	7.407	2.982	0.011	2.492	0.014	1.490	0.015	1.069	0.008	0.996	0.015	0.977	0.019	1.416	0.019	1.296	0.040	1.207	0.031	1.171	0.016
1348	7.418	2.981	0.011	2.491	0.014	1.488	0.015	1.065	0.008	0.992	0.015	0.974	0.020	1.414	0.019	1.294	0.040	1.205	0.031	1.168	0.016
1346	7.429	2.981	0.011	2.491	0.015	1.485	0.016	1.061	0.008	0.988	0.016	0.970	0.020	1.412	0.019	1.292	0.040	1.202	0.031	1.165	0.017
1344	7.440	2.980	0.011	2.490	0.015	1.483	0.016	1.057	0.009	0.983	0.016	0.966	0.020	1.409	0.020	1.290	0.041	1.199	0.032	1.162	0.017
1342	7.452	2.980	0.011	2.489	0.015	1.481	0.016	1.053	0.009	0.979	0.016	0.962	0.021	1.407	0.020	1.287	0.041	1.196	0.032	1.158	0.017
1340	7.463	2.979	0.012	2.488	0.015	1.478	0.016	1.048	0.009	0.974	0.017	0.959	0.021	1.404	0.020	1.285	0.041	1.194	0.032	1.155	0.017
1338	7.474	2.979	0.012	2.487	0.015	1.476	0.016	1.044	0.009	0.969	0.017	0.955	0.021	1.402	0.021	1.282	0.042	1.191	0.033	1.152	0.018
1336	7.485	2.978	0.012	2.486	0.015	1.473	0.017	1.039	0.009	0.964	0.017	0.951	0.021	1.399	0.021	1.280	0.042	1.188	0.033	1.149	0.018
1334	7.496	2.977	0.012	2.485	0.016	1.471	0.017	1.035	0.010	0.959	0.018	0.946	0.022	1.396	0.021	1.278	0.042	1.185	0.034	1.145	0.018
1332	7.508	2.977	0.012	2.484	0.016	1.468	0.017	1.030	0.010	0.954	0.018	0.942	0.022	1.394	0.021	1.275	0.043	1.182	0.034	1.142	0.018
1330	7.519	2.976	0.012	2.483	0.016	1.465	0.017	1.025	0.010	0.949	0.019	0.938	0.022	1.391	0.022	1.272	0.043	1.179	0.034	1.138	0.019
1328	7.530	2.976	0.012	2.482	0.016	1.463	0.018	1.020	0.011	0.943	0.019	0.933	0.023	1.388	0.022	1.270	0.044	1.176	0.035	1.134	0.019
1326	7.541	2.975	0.013	2.481	0.016	1.460	0.018	1.015	0.011	0.938	0.020	0.929	0.023	1.385	0.022	1.267	0.044	1.172	0.035	1.131	0.019
1324	7.553	2.974	0.013	2.480	0.017	1.457	0.018	1.010	0.011	0.932	0.020	0.924	0.023	1.382	0.023	1.264	0.044	1.169	0.036	1.127	0.020
1322	7.564	2.974	0.013	2.479	0.017	1.454	0.018	1.004	0.012	0.926	0.021	0.919	0.024	1.379	0.023	1.262	0.045	1.166	0.036	1.123	0.020
1320	7.576	2.973	0.013	2.478	0.017	1.452	0.019	0.999	0.012	0.920	0.022	0.914	0.024	1.376	0.023	1.259	0.045	1.162	0.037	1.119	0.020
1318	7.587	2.973	0.013	2.477	0.017	1.449	0.019	0.993	0.012	0.914	0.022	0.909	0.025	1.373	0.024	1.256	0.046	1.159	0.037	1.115	0.021
1316	7.599	2.972	0.013	2.476	0.017	1.446	0.019	0.987	0.013	0.907	0.023	0.904	0.025	1.370	0.024	1.253	0.046	1.155	0.038	1.111	0.021
1314	7.610	2.971	0.013	2.475	0.018	1.442	0.019	0.981	0.014	0.901	0.024	0.899	0.026	1.366	0.024	1.250	0.046	1.152	0.038	1.106	0.022
1312	7.622	2.971	0.014	2.474	0.018	1.439	0.020	0.975	0.014	0.894	0.025	0.893	0.026	1.363	0.025	1.247	0.047	1.148	0.039	1.102	0.022
1310	7.634	2.970	0.014	2.473	0.018	1.436	0.020	0.969	0.015	0.887	0.026	0.888	0.027	1.360	0.025	1.244	0.047	1.144	0.039	1.098	0.023
1308	7.645	2.969	0.014	2.472	0.018	1.433	0.020	0.962	0.015	0.879	0.027	0.882	0.028	1.356	0.026	1.240	0.048	1.140	0.040	1.093	0.023
1306	7.657	2.968	0.014	2.470	0.018	1.429	0.021	0.955	0.016	0.872	0.028	0.876	0.028	1.353	0.026	1.237	0.048	1.136	0.040	1.088	0.024
1304	7.669	2.968	0.014	2.469	0.019	1.426	0.021	0.948	0.017	0.864	0.029	0.870	0.029	1.349	0.026	1.234	0.049	1.132	0.041	1.083	0.024
1302	7.680	2.967	0.014	2.468	0.019	1.422	0.021	0.941	0.018	0.856	0.031	0.863	0.030	1.345	0.027	1.230	0.049	1.128	0.042	1.078	0.025
1300	7.692	2.966	0.014	2.467	0.019	1.419	0.022	0.934	0.019	0.848	0.032	0.857	0.031	1.342	0.027	1.227	0.050	1.123	0.042	1.073	0.025

Wavenumber (cm^{-1})	Wavelength (μm)	n10	k10	n20	k20	n30	k30	n40	k40	n80	k80	n120	k120	n31	k31	n32	k32	n33	k33	n34	k34
1298	7.704	2.966	0.015	2.466	0.019	1.415	0.022	0.926	0.020	0.840	0.034	0.850	0.032	1.338	0.028	1.223	0.050	1.119	0.043	1.068	0.026
1296	7.716	2.965	0.015	2.464	0.019	1.411	0.022	0.918	0.021	0.831	0.036	0.843	0.033	1.334	0.028	1.219	0.051	1.114	0.044	1.062	0.027
1294	7.728	2.964	0.015	2.463	0.020	1.407	0.023	0.910	0.023	0.822	0.038	0.836	0.034	1.330	0.029	1.215	0.051	1.110	0.044	1.057	0.028
1292	7.740	2.963	0.015	2.462	0.020	1.403	0.023	0.902	0.024	0.812	0.040	0.829	0.035	1.325	0.030	1.212	0.052	1.105	0.045	1.051	0.029
1290	7.752	2.963	0.015	2.461	0.020	1.399	0.024	0.893	0.026	0.802	0.042	0.821	0.037	1.321	0.030	1.208	0.052	1.100	0.046	1.045	0.030
1288	7.764	2.962	0.015	2.459	0.020	1.395	0.024	0.884	0.028	0.792	0.045	0.813	0.039	1.317	0.031	1.203	0.053	1.095	0.047	1.039	0.031
1286	7.776	2.961	0.016	2.458	0.021	1.391	0.025	0.874	0.030	0.782	0.048	0.805	0.040	1.312	0.032	1.199	0.054	1.090	0.048	1.033	0.032
1284	7.788	2.960	0.016	2.456	0.021	1.386	0.025	0.865	0.032	0.771	0.051	0.797	0.042	1.307	0.032	1.195	0.054	1.084	0.049	1.026	0.033
1282	7.800	2.959	0.016	2.455	0.021	1.382	0.026	0.855	0.035	0.760	0.055	0.788	0.044	1.303	0.033	1.190	0.055	1.079	0.050	1.019	0.035
1280	7.813	2.959	0.016	2.454	0.021	1.377	0.027	0.845	0.038	0.749	0.059	0.779	0.047	1.298	0.034	1.186	0.056	1.073	0.051	1.013	0.036
1278	7.825	2.958	0.016	2.452	0.022	1.372	0.027	0.834	0.041	0.737	0.063	0.770	0.049	1.293	0.035	1.181	0.057	1.067	0.053	1.005	0.038
1276	7.837	2.957	0.017	2.451	0.022	1.367	0.028	0.823	0.044	0.725	0.068	0.761	0.052	1.287	0.036	1.176	0.057	1.061	0.054	0.998	0.040
1274	7.849	2.956	0.017	2.449	0.022	1.362	0.029	0.812	0.048	0.712	0.074	0.751	0.055	1.282	0.037	1.171	0.058	1.055	0.056	0.991	0.042
1272	7.862	2.955	0.017	2.448	0.022	1.357	0.030	0.800	0.053	0.699	0.080	0.741	0.059	1.277	0.038	1.166	0.059	1.049	0.057	0.983	0.044
1270	7.874	2.954	0.017	2.446	0.023	1.351	0.031	0.788	0.057	0.686	0.087	0.731	0.062	1.271	0.040	1.160	0.060	1.042	0.059	0.975	0.047
1268	7.886	2.953	0.017	2.444	0.023	1.345	0.032	0.776	0.063	0.672	0.094	0.720	0.067	1.265	0.041	1.155	0.062	1.035	0.061	0.967	0.049
1266	7.899	2.952	0.018	2.443	0.023	1.340	0.033	0.763	0.068	0.658	0.102	0.710	0.071	1.259	0.042	1.149	0.063	1.028	0.063	0.959	0.052
1264	7.911	2.951	0.018	2.441	0.024	1.334	0.035	0.750	0.075	0.644	0.112	0.698	0.076	1.253	0.044	1.143	0.064	1.021	0.066	0.950	0.056
1262	7.924	2.950	0.018	2.439	0.024	1.328	0.036	0.736	0.082	0.630	0.122	0.687	0.082	1.247	0.046	1.137	0.066	1.014	0.068	0.941	0.059
1260	7.937	2.949	0.018	2.438	0.024	1.321	0.038	0.723	0.090	0.615	0.133	0.675	0.088	1.240	0.048	1.131	0.067	1.006	0.071	0.932	0.063
1258	7.949	2.948	0.018	2.436	0.025	1.315	0.040	0.709	0.099	0.601	0.145	0.664	0.095	1.234	0.050	1.125	0.069	0.999	0.074	0.923	0.067
1256	7.962	2.947	0.019	2.434	0.025	1.308	0.042	0.695	0.109	0.586	0.159	0.652	0.102	1.227	0.052	1.118	0.071	0.991	0.077	0.914	0.072
1254	7.974	2.946	0.019	2.432	0.026	1.301	0.044	0.681	0.119	0.572	0.174	0.640	0.110	1.220	0.055	1.111	0.073	0.983	0.080	0.904	0.077
1252	7.987	2.945	0.019	2.430	0.026	1.294	0.047	0.666	0.131	0.558	0.190	0.627	0.119	1.213	0.057	1.104	0.075	0.975	0.084	0.895	0.082
1250	8.000	2.944	0.019	2.428	0.026	1.287	0.049	0.652	0.144	0.545	0.207	0.615	0.129	1.206	0.060	1.097	0.078	0.966	0.088	0.885	0.088
1248	8.013	2.943	0.020	2.426	0.027	1.280	0.052	0.638	0.158	0.533	0.226	0.603	0.139	1.198	0.063	1.090	0.081	0.957	0.093	0.875	0.095
1246	8.026	2.942	0.020	2.424	0.027	1.272	0.055	0.624	0.173	0.521	0.246	0.591	0.150	1.190	0.067	1.082	0.084	0.949	0.098	0.865	0.101
1244	8.039	2.941	0.020	2.422	0.028	1.265	0.059	0.611	0.189	0.511	0.267	0.579	0.163	1.183	0.070	1.074	0.087	0.940	0.103	0.855	0.109
1242	8.052	2.939	0.021	2.420	0.028	1.257	0.063	0.598	0.207	0.502	0.289	0.567	0.176	1.175	0.074	1.066	0.091	0.931	0.109	0.845	0.117
1240	8.065	2.938	0.021	2.418	0.029	1.249	0.067	0.586	0.226	0.495	0.312	0.556	0.190	1.167	0.079	1.058	0.095	0.922	0.115	0.835	0.126
1238	8.078	2.937	0.021	2.415	0.030	1.241	0.071	0.576	0.246	0.489	0.335	0.546	0.205	1.158	0.083	1.050	0.099	0.913	0.121	0.825	0.135
1236	8.091	2.936	0.022	2.413	0.030	1.233	0.076	0.566	0.267	0.485	0.358	0.536	0.220	1.150	0.088	1.041	0.103	0.903	0.128	0.815	0.145

Wavenumber (cm^{-1})	Wavelength (μm)	n10	k10	n20	k20	n30	k30	n40	k40	n80	k80	n120	k120	n31	k31	n32	k32	n33	k33	n34	k34
1234	8.104	2.934	0.022	2.411	0.031	1.224	0.081	0.558	0.289	0.483	0.381	0.527	0.237	1.142	0.094	1.033	0.109	0.894	0.136	0.806	0.155
1232	8.117	2.933	0.022	2.408	0.031	1.216	0.087	0.551	0.311	0.482	0.405	0.519	0.254	1.133	0.099	1.024	0.114	0.885	0.144	0.796	0.166
1230	8.130	2.932	0.023	2.406	0.032	1.208	0.093	0.545	0.334	0.482	0.427	0.511	0.271	1.125	0.105	1.015	0.120	0.876	0.152	0.787	0.178
1228	8.143	2.930	0.023	2.403	0.033	1.200	0.099	0.541	0.357	0.483	0.450	0.505	0.289	1.116	0.112	1.007	0.126	0.867	0.162	0.779	0.190
1226	8.157	2.929	0.024	2.401	0.034	1.191	0.106	0.539	0.381	0.486	0.471	0.500	0.307	1.108	0.119	0.998	0.133	0.858	0.171	0.770	0.203
1224	8.170	2.927	0.024	2.398	0.035	1.183	0.114	0.538	0.404	0.489	0.492	0.496	0.326	1.099	0.127	0.989	0.140	0.849	0.182	0.763	0.217
1222	8.183	2.926	0.025	2.396	0.035	1.175	0.121	0.538	0.426	0.494	0.513	0.493	0.344	1.091	0.135	0.980	0.148	0.841	0.192	0.755	0.230
1220	8.197	2.925	0.025	2.393	0.036	1.167	0.130	0.539	0.449	0.499	0.532	0.491	0.362	1.083	0.143	0.972	0.157	0.832	0.204	0.749	0.245
1218	8.210	2.923	0.026	2.390	0.037	1.159	0.138	0.542	0.470	0.505	0.551	0.489	0.380	1.075	0.152	0.963	0.166	0.825	0.216	0.743	0.260
1216	8.224	2.921	0.026	2.387	0.038	1.152	0.147	0.546	0.492	0.511	0.569	0.489	0.397	1.067	0.161	0.955	0.175	0.817	0.228	0.738	0.275
1214	8.237	2.920	0.027	2.384	0.040	1.145	0.157	0.550	0.512	0.518	0.586	0.489	0.414	1.059	0.171	0.947	0.185	0.810	0.241	0.733	0.290
1212	8.251	2.918	0.027	2.381	0.041	1.138	0.167	0.555	0.532	0.525	0.603	0.490	0.430	1.051	0.181	0.939	0.196	0.804	0.254	0.730	0.306
1210	8.264	2.917	0.028	2.378	0.042	1.131	0.177	0.561	0.551	0.533	0.618	0.491	0.446	1.044	0.192	0.931	0.207	0.798	0.268	0.727	0.321
1208	8.278	2.915	0.029	2.375	0.043	1.125	0.188	0.568	0.569	0.541	0.633	0.493	0.461	1.037	0.203	0.924	0.218	0.793	0.282	0.724	0.337
1206	8.292	2.913	0.029	2.372	0.045	1.119	0.199	0.575	0.586	0.549	0.647	0.495	0.476	1.031	0.214	0.917	0.230	0.788	0.296	0.723	0.352
1204	8.306	2.911	0.030	2.369	0.046	1.113	0.210	0.582	0.603	0.556	0.661	0.497	0.490	1.024	0.226	0.911	0.242	0.784	0.311	0.722	0.367
1202	8.319	2.910	0.031	2.365	0.048	1.108	0.222	0.590	0.619	0.564	0.673	0.500	0.504	1.019	0.238	0.906	0.255	0.781	0.325	0.721	0.382
1200	8.333	2.908	0.032	2.362	0.049	1.103	0.234	0.598	0.634	0.572	0.685	0.502	0.516	1.013	0.251	0.900	0.268	0.778	0.340	0.722	0.397
1198	8.347	2.906	0.033	2.359	0.051	1.099	0.245	0.606	0.648	0.580	0.696	0.505	0.529	1.008	0.263	0.896	0.281	0.776	0.355	0.722	0.411
1196	8.361	2.904	0.033	2.355	0.053	1.095	0.257	0.614	0.662	0.588	0.706	0.507	0.541	1.004	0.276	0.892	0.295	0.775	0.369	0.723	0.425
1194	8.375	2.902	0.034	2.352	0.055	1.092	0.270	0.622	0.675	0.595	0.716	0.509	0.552	1.000	0.289	0.888	0.308	0.774	0.383	0.725	0.439
1192	8.389	2.900	0.035	2.348	0.057	1.089	0.282	0.630	0.687	0.602	0.726	0.511	0.563	0.997	0.302	0.885	0.322	0.773	0.398	0.727	0.452
1190	8.403	2.898	0.036	2.344	0.059	1.087	0.294	0.638	0.698	0.608	0.734	0.512	0.573	0.994	0.316	0.883	0.335	0.773	0.411	0.729	0.465
1188	8.418	2.896	0.038	2.341	0.062	1.084	0.306	0.645	0.709	0.614	0.743	0.513	0.583	0.991	0.329	0.881	0.348	0.774	0.425	0.731	0.477
1186	8.432	2.894	0.039	2.337	0.064	1.083	0.318	0.653	0.720	0.620	0.750	0.514	0.593	0.989	0.342	0.880	0.362	0.774	0.438	0.733	0.489
1184	8.446	2.892	0.040	2.333	0.067	1.081	0.329	0.660	0.730	0.624	0.758	0.514	0.603	0.987	0.355	0.879	0.375	0.775	0.452	0.735	0.500
1182	8.460	2.890	0.041	2.329	0.069	1.080	0.341	0.667	0.739	0.628	0.765	0.514	0.612	0.986	0.368	0.878	0.388	0.776	0.464	0.738	0.511
1180	8.475	2.888	0.043	2.325	0.072	1.079	0.352	0.673	0.748	0.632	0.772	0.512	0.621	0.985	0.381	0.878	0.400	0.778	0.477	0.740	0.522
1178	8.489	2.886	0.044	2.321	0.075	1.078	0.364	0.678	0.757	0.634	0.778	0.510	0.631	0.984	0.394	0.878	0.413	0.779	0.489	0.741	0.532
1176	8.503	2.884	0.046	2.317	0.078	1.077	0.375	0.684	0.765	0.636	0.785	0.508	0.640	0.984	0.406	0.878	0.425	0.781	0.500	0.743	0.542
1174	8.518	2.882	0.047	2.313	0.082	1.077	0.386	0.688	0.773	0.637	0.792	0.504	0.650	0.984	0.419	0.879	0.437	0.782	0.512	0.744	0.552
1172	8.532	2.879	0.049	2.309	0.085	1.076	0.397	0.692	0.782	0.637	0.799	0.500	0.660	0.984	0.431	0.879	0.448	0.784	0.523	0.745	0.561

Wavenumber (cm^{-1})	Wavelength (μm)	n10	k10	n20	k20	n30	k30	n40	k40	n80	k80	n120	k120	n31	k31	n32	k32	n33	k33	n34	k34
1170	8.547	2.877	0.051	2.305	0.089	1.075	0.407	0.695	0.790	0.636	0.806	0.495	0.671	0.984	0.443	0.880	0.460	0.785	0.534	0.745	0.571
1168	8.562	2.875	0.052	2.301	0.093	1.075	0.418	0.698	0.798	0.634	0.814	0.489	0.683	0.984	0.455	0.881	0.471	0.786	0.545	0.745	0.580
1166	8.576	2.873	0.054	2.297	0.097	1.074	0.428	0.700	0.806	0.632	0.822	0.482	0.695	0.984	0.467	0.881	0.481	0.787	0.555	0.745	0.589
1164	8.591	2.871	0.056	2.293	0.101	1.073	0.439	0.701	0.815	0.628	0.831	0.475	0.709	0.984	0.478	0.881	0.492	0.787	0.566	0.744	0.599
1162	8.606	2.868	0.058	2.289	0.106	1.072	0.449	0.702	0.824	0.623	0.841	0.467	0.723	0.984	0.490	0.881	0.502	0.788	0.576	0.742	0.609
1160	8.621	2.866	0.061	2.285	0.110	1.071	0.460	0.702	0.833	0.618	0.852	0.458	0.739	0.984	0.502	0.881	0.513	0.788	0.587	0.740	0.619
1158	8.636	2.864	0.063	2.281	0.115	1.069	0.471	0.701	0.844	0.611	0.864	0.449	0.756	0.984	0.513	0.881	0.523	0.787	0.598	0.737	0.630
1156	8.651	2.862	0.065	2.276	0.120	1.068	0.482	0.700	0.854	0.605	0.878	0.440	0.775	0.983	0.525	0.880	0.534	0.786	0.609	0.734	0.641
1154	8.666	2.859	0.068	2.273	0.125	1.066	0.493	0.698	0.866	0.597	0.893	0.431	0.796	0.983	0.537	0.880	0.544	0.785	0.620	0.730	0.652
1152	8.681	2.857	0.070	2.269	0.131	1.064	0.504	0.696	0.879	0.589	0.910	0.422	0.819	0.982	0.548	0.878	0.555	0.784	0.631	0.726	0.665
1150	8.696	2.855	0.073	2.265	0.137	1.062	0.516	0.694	0.893	0.582	0.929	0.413	0.843	0.982	0.561	0.877	0.566	0.782	0.644	0.722	0.678
1148	8.711	2.853	0.076	2.261	0.143	1.060	0.528	0.691	0.908	0.574	0.950	0.405	0.869	0.981	0.573	0.875	0.578	0.780	0.656	0.718	0.693
1146	8.726	2.850	0.078	2.257	0.149	1.058	0.541	0.688	0.924	0.566	0.973	0.398	0.897	0.980	0.586	0.873	0.590	0.778	0.669	0.713	0.708
1144	8.741	2.848	0.081	2.254	0.155	1.055	0.555	0.686	0.942	0.560	0.998	0.392	0.927	0.979	0.599	0.870	0.602	0.776	0.683	0.708	0.725
1142	8.757	2.846	0.084	2.250	0.162	1.053	0.569	0.683	0.961	0.554	1.024	0.387	0.959	0.978	0.613	0.868	0.615	0.774	0.698	0.704	0.743
1140	8.772	2.844	0.088	2.247	0.169	1.051	0.583	0.681	0.981	0.549	1.053	0.383	0.992	0.977	0.627	0.865	0.629	0.771	0.714	0.699	0.762
1138	8.787	2.842	0.091	2.243	0.176	1.049	0.599	0.680	1.003	0.545	1.084	0.382	1.026	0.975	0.642	0.863	0.644	0.769	0.730	0.695	0.782
1136	8.803	2.840	0.094	2.240	0.184	1.047	0.615	0.680	1.026	0.543	1.117	0.382	1.062	0.974	0.657	0.860	0.659	0.767	0.748	0.692	0.804
1134	8.818	2.838	0.098	2.237	0.191	1.046	0.631	0.680	1.051	0.543	1.151	0.384	1.100	0.974	0.673	0.858	0.675	0.765	0.766	0.689	0.826
1132	8.834	2.836	0.102	2.234	0.199	1.045	0.649	0.682	1.076	0.545	1.187	0.388	1.138	0.973	0.690	0.856	0.692	0.764	0.785	0.688	0.850
1130	8.850	2.835	0.105	2.232	0.207	1.045	0.667	0.685	1.103	0.550	1.224	0.394	1.177	0.973	0.707	0.854	0.710	0.764	0.806	0.687	0.876
1128	8.865	2.833	0.109	2.229	0.216	1.045	0.686	0.689	1.131	0.556	1.262	0.403	1.217	0.973	0.725	0.853	0.729	0.764	0.827	0.688	0.902
1126	8.881	2.831	0.113	2.227	0.224	1.046	0.706	0.695	1.160	0.565	1.301	0.413	1.257	0.974	0.744	0.852	0.749	0.765	0.849	0.690	0.929
1124	8.897	2.829	0.117	2.225	0.233	1.048	0.726	0.703	1.190	0.577	1.341	0.426	1.298	0.976	0.763	0.852	0.770	0.767	0.872	0.693	0.957
1122	8.913	2.828	0.121	2.224	0.242	1.051	0.747	0.712	1.220	0.591	1.381	0.442	1.339	0.978	0.784	0.853	0.791	0.770	0.895	0.698	0.985
1120	8.929	2.827	0.126	2.222	0.252	1.054	0.768	0.724	1.251	0.608	1.421	0.460	1.380	0.981	0.804	0.855	0.813	0.774	0.919	0.704	1.014
1118	8.945	2.825	0.130	2.221	0.261	1.059	0.789	0.737	1.282	0.627	1.462	0.480	1.421	0.985	0.826	0.858	0.836	0.779	0.944	0.713	1.044
1116	8.961	2.824	0.134	2.220	0.271	1.065	0.811	0.753	1.314	0.649	1.502	0.503	1.461	0.989	0.847	0.863	0.859	0.786	0.970	0.722	1.074
1114	8.977	2.823	0.139	2.220	0.281	1.072	0.833	0.770	1.345	0.674	1.542	0.528	1.502	0.995	0.870	0.868	0.883	0.794	0.995	0.734	1.104
1112	8.993	2.822	0.144	2.219	0.291	1.080	0.855	0.789	1.376	0.702	1.582	0.555	1.541	1.002	0.892	0.875	0.908	0.803	1.021	0.747	1.134
1110	9.009	2.821	0.148	2.219	0.301	1.089	0.877	0.810	1.407	0.732	1.620	0.585	1.580	1.010	0.915	0.883	0.932	0.814	1.047	0.762	1.164
1108	9.025	2.820	0.153	2.220	0.311	1.099	0.900	0.834	1.437	0.765	1.658	0.618	1.618	1.019	0.938	0.892	0.957	0.826	1.073	0.779	1.193

Wavenumber (cm^{-1})	Wavelength (μm)	n10	k10	n20	k20	n30	k30	n40	k40	n80	k80	n120	k120	n31	k31	n32	k32	n33	k33	n34	k34
1106	9.042	2.820	0.158	2.221	0.322	1.111	0.922	0.859	1.467	0.800	1.695	0.652	1.655	1.030	0.961	0.903	0.981	0.840	1.099	0.797	1.223
1104	9.058	2.819	0.163	2.222	0.332	1.124	0.943	0.886	1.496	0.838	1.730	0.689	1.691	1.042	0.984	0.915	1.006	0.855	1.125	0.818	1.251
1102	9.074	2.819	0.168	2.223	0.343	1.137	0.965	0.915	1.524	0.878	1.764	0.729	1.726	1.054	1.007	0.928	1.030	0.871	1.151	0.839	1.279
1100	9.091	2.819	0.174	2.225	0.353	1.152	0.986	0.946	1.551	0.921	1.796	0.770	1.758	1.068	1.029	0.943	1.054	0.889	1.176	0.863	1.307
1098	9.107	2.818	0.179	2.228	0.364	1.168	1.006	0.979	1.577	0.966	1.826	0.814	1.790	1.084	1.052	0.959	1.078	0.909	1.200	0.887	1.333
1096	9.124	2.819	0.184	2.230	0.375	1.185	1.026	1.013	1.601	1.013	1.854	0.860	1.819	1.100	1.073	0.976	1.101	0.930	1.224	0.914	1.359
1094	9.141	2.819	0.189	2.234	0.386	1.203	1.045	1.049	1.624	1.062	1.880	0.907	1.847	1.118	1.095	0.995	1.124	0.952	1.248	0.941	1.384
1092	9.158	2.819	0.195	2.237	0.397	1.222	1.064	1.087	1.646	1.113	1.904	0.957	1.872	1.137	1.116	1.015	1.146	0.975	1.270	0.971	1.408
1090	9.174	2.820	0.200	2.241	0.408	1.242	1.082	1.126	1.666	1.166	1.925	1.008	1.895	1.157	1.136	1.035	1.167	0.999	1.292	1.001	1.430
1088	9.191	2.820	0.206	2.246	0.418	1.263	1.099	1.166	1.684	1.220	1.944	1.061	1.916	1.177	1.156	1.057	1.188	1.025	1.313	1.033	1.451
1086	9.208	2.821	0.211	2.251	0.429	1.284	1.115	1.207	1.700	1.276	1.960	1.115	1.934	1.199	1.175	1.081	1.208	1.052	1.333	1.066	1.471
1084	9.225	2.822	0.217	2.256	0.440	1.306	1.131	1.250	1.715	1.333	1.973	1.171	1.950	1.222	1.193	1.105	1.226	1.080	1.352	1.100	1.490
1082	9.242	2.824	0.222	2.262	0.450	1.329	1.146	1.293	1.727	1.391	1.984	1.227	1.963	1.246	1.210	1.130	1.244	1.109	1.369	1.135	1.507
1080	9.259	2.825	0.228	2.268	0.461	1.353	1.159	1.338	1.738	1.450	1.991	1.285	1.973	1.271	1.226	1.156	1.261	1.139	1.386	1.170	1.523
1078	9.276	2.827	0.233	2.274	0.471	1.377	1.172	1.383	1.747	1.509	1.995	1.344	1.980	1.296	1.241	1.182	1.277	1.170	1.401	1.207	1.537
1076	9.294	2.828	0.239	2.281	0.481	1.401	1.184	1.429	1.753	1.569	1.996	1.403	1.985	1.323	1.256	1.210	1.292	1.201	1.415	1.245	1.550
1074	9.311	2.830	0.244	2.289	0.491	1.427	1.195	1.475	1.757	1.630	1.995	1.462	1.986	1.349	1.269	1.238	1.305	1.233	1.428	1.283	1.561
1072	9.328	2.832	0.250	2.297	0.501	1.452	1.205	1.521	1.759	1.690	1.990	1.522	1.985	1.377	1.281	1.267	1.318	1.266	1.439	1.322	1.570
1070	9.346	2.835	0.255	2.305	0.510	1.478	1.213	1.568	1.759	1.750	1.981	1.582	1.980	1.405	1.292	1.296	1.329	1.300	1.450	1.361	1.578
1068	9.363	2.837	0.261	2.313	0.520	1.504	1.221	1.615	1.756	1.810	1.970	1.642	1.972	1.434	1.302	1.326	1.339	1.334	1.458	1.401	1.584
1066	9.381	2.840	0.266	2.322	0.529	1.531	1.228	1.662	1.752	1.870	1.955	1.702	1.962	1.463	1.311	1.356	1.348	1.368	1.465	1.441	1.588
1064	9.398	2.843	0.272	2.332	0.537	1.557	1.234	1.709	1.745	1.929	1.938	1.761	1.948	1.492	1.318	1.387	1.356	1.403	1.471	1.481	1.591
1062	9.416	2.846	0.277	2.341	0.546	1.584	1.239	1.755	1.735	1.986	1.917	1.819	1.931	1.522	1.325	1.418	1.362	1.438	1.476	1.522	1.591
1060	9.434	2.849	0.282	2.351	0.554	1.611	1.242	1.801	1.724	2.043	1.893	1.877	1.911	1.552	1.330	1.449	1.367	1.474	1.478	1.562	1.590
1058	9.452	2.852	0.287	2.362	0.561	1.638	1.245	1.846	1.710	2.098	1.867	1.933	1.889	1.583	1.334	1.481	1.371	1.509	1.480	1.603	1.588
1056	9.470	2.856	0.292	2.372	0.569	1.665	1.247	1.891	1.695	2.152	1.837	1.988	1.863	1.613	1.337	1.512	1.374	1.545	1.480	1.643	1.583
1054	9.488	2.860	0.297	2.383	0.576	1.692	1.247	1.934	1.677	2.204	1.805	2.042	1.835	1.643	1.338	1.544	1.375	1.580	1.478	1.683	1.577
1052	9.506	2.864	0.302	2.395	0.582	1.719	1.247	1.977	1.657	2.254	1.770	2.094	1.804	1.674	1.339	1.576	1.375	1.616	1.475	1.723	1.569
1050	9.524	2.868	0.307	2.406	0.589	1.746	1.246	2.019	1.635	2.301	1.733	2.144	1.771	1.704	1.338	1.607	1.374	1.651	1.470	1.762	1.559
1048	9.542	2.872	0.312	2.418	0.594	1.772	1.243	2.059	1.612	2.347	1.694	2.192	1.735	1.735	1.336	1.639	1.372	1.686	1.464	1.801	1.548
1046	9.560	2.876	0.317	2.430	0.600	1.799	1.240	2.098	1.586	2.391	1.652	2.238	1.697	1.765	1.332	1.670	1.368	1.721	1.457	1.839	1.535
1044	9.579	2.881	0.321	2.442	0.605	1.825	1.236	2.136	1.559	2.431	1.609	2.282	1.656	1.795	1.328	1.701	1.363	1.755	1.448	1.876	1.520

Wavenumber (cm^{-1})	Wavelength (μm)	n10	k10	n20	k20	n30	k30	n40	k40	n80	k80	n120	k120	n31	k31	n32	k32	n33	k33	n34	k34
1042	9.597	2.885	0.325	2.454	0.609	1.851	1.231	2.172	1.530	2.470	1.563	2.324	1.614	1.825	1.322	1.732	1.357	1.789	1.438	1.913	1.504
1040	9.615	2.890	0.330	2.467	0.613	1.876	1.225	2.207	1.500	2.505	1.517	2.363	1.570	1.854	1.315	1.762	1.350	1.822	1.426	1.949	1.486
1038	9.634	2.895	0.334	2.479	0.617	1.901	1.218	2.239	1.468	2.538	1.468	2.399	1.525	1.883	1.307	1.792	1.341	1.855	1.413	1.984	1.467
1036	9.653	2.900	0.338	2.492	0.620	1.926	1.210	2.270	1.435	2.568	1.419	2.433	1.478	1.912	1.298	1.822	1.332	1.887	1.399	2.018	1.446
1034	9.671	2.906	0.342	2.505	0.623	1.951	1.201	2.300	1.401	2.595	1.369	2.464	1.429	1.940	1.288	1.851	1.321	1.918	1.383	2.051	1.424
1032	9.690	2.911	0.345	2.518	0.625	1.975	1.192	2.327	1.365	2.619	1.318	2.492	1.380	1.967	1.277	1.880	1.310	1.948	1.366	2.083	1.401
1030	9.709	2.916	0.349	2.531	0.627	1.998	1.181	2.353	1.329	2.641	1.266	2.518	1.330	1.994	1.265	1.907	1.297	1.978	1.348	2.113	1.376
1028	9.728	2.922	0.352	2.544	0.628	2.021	1.170	2.376	1.292	2.659	1.214	2.540	1.279	2.021	1.252	1.935	1.283	2.007	1.329	2.142	1.351
1026	9.747	2.928	0.355	2.557	0.629	2.043	1.158	2.398	1.254	2.674	1.161	2.560	1.228	2.046	1.237	1.961	1.268	2.034	1.309	2.170	1.324
1024	9.766	2.933	0.358	2.570	0.630	2.065	1.146	2.417	1.215	2.687	1.109	2.577	1.176	2.071	1.222	1.987	1.252	2.061	1.288	2.197	1.296
1022	9.785	2.939	0.361	2.583	0.630	2.086	1.133	2.434	1.177	2.697	1.057	2.591	1.124	2.095	1.207	2.012	1.236	2.087	1.266	2.222	1.267
1020	9.804	2.945	0.364	2.596	0.629	2.107	1.119	2.450	1.137	2.704	1.005	2.602	1.073	2.119	1.190	2.037	1.218	2.111	1.243	2.246	1.238
1018	9.823	2.951	0.366	2.608	0.628	2.126	1.104	2.463	1.098	2.708	0.954	2.610	1.021	2.141	1.172	2.060	1.200	2.135	1.219	2.268	1.208
1016	9.843	2.958	0.369	2.621	0.627	2.146	1.089	2.474	1.059	2.709	0.904	2.616	0.970	2.163	1.154	2.083	1.181	2.157	1.194	2.289	1.177
1014	9.862	2.964	0.371	2.634	0.625	2.164	1.073	2.483	1.019	2.709	0.854	2.619	0.920	2.184	1.135	2.104	1.161	2.178	1.169	2.308	1.145
1012	9.881	2.970	0.373	2.646	0.623	2.182	1.057	2.491	0.980	2.705	0.806	2.620	0.871	2.204	1.115	2.125	1.141	2.198	1.143	2.326	1.113
1010	9.901	2.976	0.374	2.659	0.620	2.199	1.041	2.496	0.941	2.700	0.758	2.618	0.822	2.223	1.095	2.145	1.120	2.216	1.116	2.342	1.080
1008	9.921	2.983	0.376	2.671	0.617	2.215	1.024	2.499	0.903	2.692	0.712	2.614	0.774	2.241	1.075	2.164	1.098	2.234	1.089	2.356	1.048
1006	9.940	2.989	0.377	2.683	0.613	2.231	1.006	2.501	0.865	2.682	0.668	2.607	0.728	2.258	1.053	2.182	1.076	2.250	1.061	2.369	1.015
1004	9.960	2.996	0.378	2.695	0.609	2.245	0.988	2.501	0.827	2.670	0.625	2.599	0.683	2.274	1.032	2.198	1.053	2.264	1.034	2.380	0.981
1002	9.980	3.002	0.379	2.707	0.605	2.259	0.970	2.499	0.791	2.657	0.583	2.588	0.639	2.290	1.009	2.214	1.030	2.278	1.005	2.390	0.948
1000	10.000	3.009	0.380	2.718	0.600	2.272	0.951	2.495	0.755	2.642	0.543	2.576	0.597	2.304	0.987	2.229	1.006	2.290	0.977	2.399	0.915
998	10.020	3.015	0.380	2.730	0.595	2.285	0.933	2.490	0.720	2.625	0.505	2.562	0.556	2.317	0.964	2.243	0.982	2.300	0.948	2.405	0.881
996	10.040	3.022	0.381	2.741	0.590	2.296	0.914	2.483	0.686	2.607	0.469	2.546	0.518	2.329	0.941	2.255	0.958	2.310	0.919	2.410	0.848
994	10.060	3.028	0.381	2.752	0.584	2.307	0.894	2.475	0.653	2.588	0.434	2.529	0.480	2.340	0.918	2.267	0.934	2.318	0.891	2.414	0.815
992	10.081	3.035	0.381	2.762	0.578	2.317	0.875	2.466	0.621	2.568	0.401	2.511	0.445	2.350	0.894	2.277	0.909	2.325	0.862	2.416	0.783
990	10.101	3.041	0.381	2.772	0.572	2.326	0.856	2.456	0.591	2.547	0.370	2.492	0.411	2.359	0.871	2.287	0.884	2.330	0.833	2.417	0.751
988	10.121	3.048	0.380	2.782	0.565	2.334	0.836	2.444	0.561	2.526	0.341	2.471	0.379	2.367	0.847	2.295	0.860	2.335	0.805	2.417	0.719
986	10.142	3.054	0.379	2.792	0.558	2.342	0.816	2.431	0.533	2.503	0.314	2.450	0.349	2.374	0.824	2.302	0.835	2.338	0.776	2.415	0.688
984	10.163	3.060	0.379	2.801	0.551	2.349	0.797	2.418	0.506	2.481	0.288	2.428	0.321	2.380	0.800	2.309	0.810	2.340	0.748	2.412	0.657
982	10.183	3.067	0.377	2.810	0.543	2.355	0.777	2.403	0.481	2.458	0.264	2.406	0.295	2.385	0.776	2.314	0.785	2.340	0.721	2.407	0.627
980	10.204	3.073	0.376	2.819	0.535	2.360	0.758	2.388	0.457	2.434	0.242	2.383	0.270	2.389	0.753	2.318	0.761	2.340	0.693	2.402	0.598

Wavenumber (cm^{-1})	Wavelength (μm)	n10	k10	n20	k20	n30	k30	n40	k40	n80	k80	n120	k120	n31	k31	n32	k32	n33	k33	n34	k34
978	10.225	3.079	0.375	2.827	0.527	2.364	0.738	2.372	0.434	2.411	0.222	2.360	0.247	2.392	0.730	2.321	0.736	2.338	0.667	2.395	0.569
976	10.246	3.085	0.373	2.835	0.519	2.368	0.719	2.356	0.413	2.388	0.203	2.336	0.226	2.395	0.707	2.324	0.712	2.336	0.640	2.387	0.541
974	10.267	3.091	0.371	2.842	0.511	2.371	0.700	2.339	0.392	2.364	0.186	2.313	0.206	2.396	0.684	2.325	0.688	2.332	0.614	2.378	0.514
972	10.288	3.097	0.369	2.849	0.502	2.373	0.681	2.322	0.374	2.341	0.170	2.289	0.188	2.396	0.662	2.325	0.664	2.327	0.589	2.368	0.488
970	10.309	3.103	0.367	2.856	0.493	2.375	0.662	2.305	0.356	2.318	0.156	2.266	0.171	2.396	0.639	2.325	0.641	2.321	0.564	2.357	0.463
968	10.331	3.109	0.365	2.863	0.485	2.376	0.643	2.288	0.340	2.296	0.143	2.243	0.156	2.394	0.618	2.323	0.618	2.315	0.540	2.345	0.439
966	10.352	3.115	0.362	2.869	0.476	2.376	0.625	2.270	0.325	2.273	0.131	2.220	0.143	2.392	0.596	2.321	0.595	2.307	0.517	2.333	0.416
964	10.373	3.120	0.360	2.875	0.466	2.375	0.607	2.253	0.311	2.252	0.120	2.198	0.130	2.389	0.575	2.318	0.573	2.299	0.495	2.319	0.393
962	10.395	3.126	0.357	2.880	0.457	2.374	0.589	2.235	0.299	2.230	0.111	2.175	0.119	2.385	0.555	2.314	0.551	2.290	0.473	2.305	0.372
960	10.417	3.131	0.354	2.885	0.448	2.373	0.572	2.218	0.288	2.210	0.102	2.154	0.109	2.381	0.535	2.309	0.529	2.280	0.452	2.290	0.352
958	10.438	3.136	0.351	2.890	0.439	2.370	0.555	2.201	0.277	2.189	0.094	2.132	0.100	2.375	0.516	2.304	0.509	2.269	0.432	2.275	0.333
956	10.460	3.141	0.347	2.894	0.429	2.367	0.538	2.185	0.268	2.170	0.088	2.112	0.092	2.369	0.497	2.297	0.488	2.258	0.412	2.259	0.315
954	10.482	3.146	0.344	2.898	0.420	2.364	0.522	2.168	0.260	2.151	0.082	2.092	0.085	2.363	0.478	2.291	0.469	2.246	0.394	2.243	0.298
952	10.504	3.151	0.341	2.901	0.410	2.360	0.507	2.153	0.252	2.132	0.076	2.072	0.079	2.356	0.461	2.283	0.449	2.234	0.376	2.226	0.282
950	10.526	3.156	0.337	2.904	0.401	2.356	0.491	2.137	0.246	2.114	0.072	2.053	0.074	2.348	0.443	2.275	0.431	2.221	0.359	2.209	0.268
948	10.549	3.160	0.333	2.907	0.392	2.351	0.476	2.122	0.240	2.097	0.068	2.035	0.069	2.340	0.427	2.266	0.413	2.207	0.344	2.192	0.254
946	10.571	3.164	0.329	2.910	0.382	2.346	0.462	2.107	0.235	2.080	0.064	2.017	0.065	2.331	0.411	2.256	0.396	2.193	0.329	2.174	0.242
944	10.593	3.169	0.325	2.912	0.373	2.340	0.448	2.093	0.230	2.063	0.061	2.000	0.062	2.322	0.396	2.246	0.379	2.179	0.315	2.156	0.231
942	10.616	3.173	0.321	2.914	0.364	2.334	0.435	2.080	0.226	2.048	0.059	1.983	0.059	2.312	0.381	2.236	0.363	2.165	0.302	2.138	0.221
940	10.638	3.177	0.317	2.915	0.354	2.328	0.422	2.067	0.223	2.032	0.056	1.967	0.057	2.302	0.368	2.225	0.348	2.150	0.290	2.121	0.212
938	10.661	3.180	0.313	2.916	0.345	2.321	0.409	2.054	0.220	2.018	0.055	1.951	0.055	2.291	0.354	2.213	0.334	2.135	0.279	2.103	0.204
936	10.684	3.184	0.308	2.917	0.336	2.314	0.397	2.042	0.218	2.003	0.053	1.936	0.053	2.280	0.342	2.201	0.320	2.120	0.270	2.085	0.197
934	10.707	3.187	0.304	2.917	0.327	2.307	0.386	2.030	0.215	1.990	0.052	1.922	0.052	2.269	0.330	2.189	0.307	2.104	0.261	2.068	0.192
932	10.730	3.190	0.299	2.918	0.319	2.299	0.375	2.019	0.214	1.976	0.051	1.908	0.051	2.258	0.320	2.176	0.295	2.089	0.253	2.050	0.188
930	10.753	3.193	0.295	2.918	0.310	2.292	0.364	2.009	0.212	1.963	0.050	1.894	0.050	2.246	0.309	2.163	0.284	2.074	0.246	2.034	0.184
928	10.776	3.196	0.290	2.917	0.301	2.284	0.355	1.999	0.211	1.951	0.049	1.881	0.049	2.234	0.300	2.150	0.274	2.059	0.240	2.017	0.182
926	10.799	3.199	0.286	2.916	0.293	2.276	0.345	1.989	0.210	1.938	0.049	1.868	0.049	2.222	0.291	2.136	0.264	2.044	0.235	2.001	0.181
924	10.823	3.201	0.281	2.916	0.285	2.268	0.336	1.980	0.209	1.926	0.049	1.856	0.049	2.210	0.284	2.122	0.256	2.029	0.231	1.986	0.180
922	10.846	3.204	0.276	2.914	0.277	2.259	0.328	1.971	0.208	1.915	0.048	1.844	0.049	2.198	0.277	2.108	0.248	2.015	0.228	1.971	0.181
920	10.870	3.206	0.271	2.913	0.269	2.251	0.320	1.962	0.207	1.904	0.048	1.833	0.049	2.186	0.271	2.094	0.241	2.001	0.225	1.957	0.182
918	10.893	3.208	0.267	2.911	0.261	2.243	0.313	1.954	0.206	1.893	0.048	1.821	0.049	2.174	0.265	2.080	0.235	1.987	0.224	1.944	0.185
916	10.917	3.209	0.262	2.910	0.254	2.234	0.306	1.946	0.205	1.882	0.049	1.810	0.049	2.161	0.261	2.066	0.231	1.974	0.224	1.932	0.187

Wavenumber (cm^{-1})	Wavelength (μm)	n10	k10	n20	k20	n30	k30	n40	k40	n80	k80	n120	k120	n31	k31	n32	k32	n33	k33	n34	k34
914	10.941	3.211	0.257	2.908	0.246	2.226	0.300	1.938	0.205	1.871	0.049	1.800	0.049	2.149	0.258	2.052	0.227	1.961	0.224	1.920	0.191
912	10.965	3.213	0.253	2.905	0.239	2.217	0.294	1.930	0.204	1.861	0.049	1.789	0.050	2.138	0.255	2.039	0.224	1.949	0.225	1.909	0.195
910	10.989	3.214	0.248	2.903	0.232	2.209	0.288	1.923	0.204	1.851	0.050	1.779	0.050	2.126	0.253	2.025	0.222	1.938	0.226	1.900	0.199
908	11.013	3.215	0.243	2.900	0.226	2.201	0.284	1.916	0.203	1.841	0.050	1.769	0.051	2.116	0.252	2.013	0.221	1.927	0.228	1.891	0.203
906	11.038	3.216	0.238	2.898	0.219	2.193	0.279	1.909	0.202	1.831	0.051	1.759	0.051	2.105	0.252	2.000	0.221	1.917	0.231	1.883	0.208
904	11.062	3.217	0.234	2.895	0.213	2.185	0.275	1.902	0.202	1.821	0.052	1.749	0.052	2.095	0.252	1.988	0.222	1.908	0.234	1.876	0.213
902	11.086	3.217	0.229	2.892	0.207	2.177	0.271	1.895	0.202	1.812	0.053	1.739	0.053	2.086	0.253	1.977	0.223	1.899	0.237	1.869	0.217
900	11.111	3.218	0.225	2.888	0.201	2.169	0.268	1.888	0.201	1.802	0.053	1.730	0.054	2.077	0.254	1.966	0.226	1.892	0.241	1.864	0.222
898	11.136	3.218	0.220	2.885	0.195	2.161	0.265	1.882	0.201	1.793	0.055	1.720	0.055	2.070	0.256	1.957	0.228	1.885	0.245	1.859	0.226
896	11.161	3.218	0.216	2.882	0.190	2.153	0.262	1.875	0.201	1.783	0.056	1.711	0.056	2.063	0.258	1.948	0.232	1.878	0.249	1.855	0.230
894	11.186	3.218	0.211	2.878	0.185	2.146	0.260	1.869	0.201	1.774	0.057	1.701	0.057	2.056	0.261	1.940	0.236	1.873	0.253	1.852	0.234
892	11.211	3.218	0.207	2.875	0.180	2.139	0.258	1.862	0.201	1.765	0.059	1.692	0.058	2.051	0.263	1.933	0.240	1.868	0.257	1.849	0.237
890	11.236	3.218	0.203	2.871	0.175	2.132	0.257	1.856	0.201	1.756	0.061	1.682	0.060	2.046	0.266	1.927	0.244	1.864	0.261	1.847	0.240
888	11.261	3.218	0.198	2.867	0.170	2.125	0.256	1.849	0.201	1.747	0.063	1.673	0.062	2.042	0.268	1.922	0.248	1.860	0.264	1.845	0.243
886	11.287	3.217	0.194	2.864	0.166	2.119	0.255	1.843	0.201	1.737	0.065	1.663	0.064	2.039	0.270	1.917	0.252	1.857	0.268	1.843	0.245
884	11.312	3.217	0.190	2.860	0.162	2.112	0.254	1.837	0.202	1.728	0.067	1.654	0.066	2.036	0.272	1.914	0.256	1.855	0.271	1.841	0.246
882	11.338	3.216	0.186	2.856	0.158	2.106	0.253	1.831	0.203	1.719	0.070	1.645	0.068	2.034	0.273	1.911	0.260	1.852	0.274	1.839	0.248
880	11.364	3.215	0.182	2.852	0.154	2.100	0.253	1.824	0.204	1.710	0.073	1.635	0.071	2.032	0.274	1.909	0.263	1.851	0.276	1.838	0.249
878	11.390	3.214	0.179	2.848	0.151	2.095	0.253	1.818	0.205	1.701	0.076	1.626	0.074	2.030	0.275	1.908	0.266	1.849	0.278	1.836	0.249
876	11.416	3.213	0.175	2.844	0.147	2.089	0.253	1.812	0.206	1.692	0.080	1.616	0.078	2.028	0.275	1.906	0.268	1.848	0.280	1.834	0.249
874	11.442	3.212	0.171	2.841	0.144	2.084	0.253	1.807	0.208	1.683	0.084	1.607	0.082	2.027	0.274	1.906	0.270	1.847	0.282	1.832	0.249
872	11.468	3.211	0.168	2.837	0.141	2.079	0.254	1.801	0.210	1.675	0.089	1.598	0.087	2.025	0.274	1.905	0.271	1.846	0.283	1.830	0.249
870	11.494	3.210	0.164	2.833	0.138	2.074	0.255	1.795	0.212	1.666	0.094	1.588	0.092	2.023	0.273	1.905	0.271	1.845	0.284	1.828	0.249
868	11.521	3.208	0.161	2.829	0.136	2.070	0.255	1.790	0.214	1.658	0.100	1.579	0.097	2.021	0.271	1.904	0.271	1.844	0.284	1.826	0.249
866	11.547	3.207	0.158	2.825	0.133	2.066	0.256	1.785	0.217	1.650	0.106	1.570	0.103	2.019	0.270	1.904	0.271	1.843	0.285	1.823	0.248
864	11.574	3.205	0.155	2.821	0.131	2.062	0.257	1.780	0.220	1.642	0.112	1.562	0.110	2.016	0.269	1.903	0.270	1.841	0.285	1.820	0.248
862	11.601	3.204	0.152	2.817	0.129	2.058	0.258	1.775	0.223	1.635	0.119	1.554	0.118	2.013	0.267	1.902	0.268	1.840	0.285	1.817	0.248
860	11.628	3.202	0.149	2.814	0.127	2.054	0.259	1.771	0.226	1.628	0.127	1.546	0.126	2.010	0.266	1.900	0.267	1.839	0.285	1.814	0.248
858	11.655	3.200	0.146	2.810	0.125	2.051	0.260	1.767	0.230	1.621	0.135	1.539	0.134	2.007	0.265	1.898	0.265	1.838	0.285	1.811	0.248
856	11.682	3.198	0.143	2.806	0.123	2.048	0.262	1.763	0.234	1.615	0.144	1.532	0.144	2.004	0.264	1.896	0.263	1.836	0.285	1.807	0.249
854	11.710	3.197	0.141	2.803	0.122	2.045	0.263	1.759	0.238	1.610	0.153	1.526	0.153	2.000	0.263	1.894	0.262	1.835	0.285	1.804	0.250
852	11.737	3.195	0.138	2.799	0.120	2.043	0.264	1.756	0.242	1.605	0.163	1.521	0.164	1.997	0.263	1.891	0.260	1.833	0.285	1.801	0.251

Wavenumber (cm^{-1})	Wavelength (µm)	n10	k10	n20	k20	n30	k30	n40	k40	n80	k80	n120	k120	n31	k31	n32	k32	n33	k33	n34	k34
850	11.765	3.193	0.136	2.796	0.119	2.040	0.265	1.754	0.246	1.602	0.173	1.517	0.175	1.993	0.263	1.887	0.259	1.832	0.285	1.798	0.252
848	11.792	3.191	0.134	2.792	0.118	2.038	0.267	1.751	0.250	1.599	0.183	1.513	0.186	1.990	0.263	1.884	0.258	1.830	0.285	1.795	0.254
846	11.820	3.189	0.131	2.789	0.117	2.036	0.268	1.749	0.255	1.597	0.194	1.511	0.198	1.987	0.263	1.880	0.257	1.828	0.285	1.792	0.255
844	11.848	3.187	0.129	2.785	0.116	2.034	0.269	1.748	0.259	1.596	0.204	1.510	0.209	1.984	0.264	1.876	0.257	1.827	0.285	1.789	0.258
842	11.876	3.185	0.127	2.782	0.115	2.032	0.270	1.747	0.263	1.596	0.215	1.510	0.221	1.981	0.265	1.872	0.257	1.825	0.286	1.787	0.260
840	11.905	3.183	0.125	2.779	0.114	2.031	0.271	1.746	0.268	1.597	0.226	1.511	0.233	1.978	0.266	1.868	0.257	1.823	0.286	1.785	0.263
838	11.933	3.181	0.124	2.776	0.113	2.030	0.273	1.746	0.272	1.599	0.236	1.514	0.245	1.975	0.267	1.864	0.258	1.822	0.287	1.783	0.265
836	11.962	3.179	0.122	2.773	0.113	2.028	0.274	1.746	0.276	1.603	0.246	1.517	0.256	1.973	0.268	1.860	0.260	1.820	0.288	1.781	0.268
834	11.990	3.177	0.120	2.770	0.112	2.027	0.275	1.747	0.280	1.607	0.256	1.522	0.266	1.971	0.270	1.857	0.262	1.819	0.289	1.780	0.271
832	12.019	3.175	0.119	2.767	0.112	2.026	0.276	1.748	0.284	1.612	0.265	1.528	0.276	1.969	0.271	1.853	0.264	1.818	0.290	1.780	0.274
830	12.048	3.173	0.117	2.764	0.111	2.026	0.277	1.749	0.288	1.619	0.273	1.535	0.286	1.968	0.273	1.851	0.267	1.817	0.291	1.780	0.277
828	12.077	3.171	0.116	2.761	0.111	2.025	0.277	1.751	0.291	1.626	0.281	1.543	0.294	1.966	0.274	1.848	0.270	1.817	0.292	1.780	0.280
826	12.107	3.169	0.115	2.758	0.111	2.024	0.278	1.753	0.294	1.633	0.287	1.552	0.301	1.965	0.275	1.846	0.273	1.816	0.293	1.780	0.283
824	12.136	3.166	0.114	2.756	0.111	2.024	0.279	1.756	0.297	1.642	0.293	1.561	0.308	1.965	0.277	1.845	0.276	1.816	0.294	1.781	0.286
822	12.165	3.164	0.113	2.753	0.111	2.024	0.279	1.759	0.300	1.651	0.298	1.571	0.313	1.964	0.278	1.844	0.280	1.816	0.295	1.782	0.288
820	12.195	3.162	0.112	2.751	0.111	2.024	0.280	1.761	0.302	1.660	0.301	1.582	0.317	1.963	0.279	1.844	0.283	1.816	0.297	1.784	0.290
818	12.225	3.160	0.111	2.748	0.111	2.023	0.280	1.765	0.304	1.670	0.304	1.593	0.319	1.963	0.280	1.844	0.286	1.816	0.298	1.786	0.293
816	12.255	3.158	0.110	2.746	0.111	2.023	0.281	1.768	0.305	1.680	0.305	1.604	0.321	1.963	0.281	1.845	0.290	1.817	0.299	1.788	0.294
814	12.285	3.157	0.109	2.743	0.111	2.023	0.281	1.772	0.306	1.690	0.305	1.614	0.321	1.963	0.282	1.846	0.293	1.817	0.299	1.790	0.296
812	12.315	3.155	0.108	2.741	0.111	2.023	0.281	1.775	0.307	1.700	0.305	1.625	0.320	1.963	0.283	1.848	0.295	1.818	0.300	1.793	0.297
810	12.346	3.153	0.108	2.739	0.111	2.023	0.281	1.779	0.307	1.709	0.303	1.636	0.317	1.963	0.284	1.850	0.298	1.819	0.301	1.796	0.298
808	12.376	3.151	0.107	2.737	0.112	2.024	0.281	1.783	0.307	1.718	0.300	1.646	0.314	1.964	0.284	1.852	0.300	1.821	0.301	1.799	0.298
806	12.407	3.149	0.107	2.735	0.112	2.024	0.281	1.787	0.306	1.727	0.296	1.656	0.310	1.964	0.284	1.855	0.302	1.822	0.301	1.802	0.299
804	12.438	3.147	0.106	2.732	0.112	2.024	0.281	1.790	0.305	1.736	0.291	1.665	0.304	1.965	0.285	1.858	0.303	1.824	0.301	1.805	0.298
802	12.469	3.145	0.106	2.731	0.112	2.024	0.281	1.794	0.304	1.743	0.285	1.673	0.298	1.965	0.285	1.862	0.304	1.825	0.301	1.808	0.298
800	12.500	3.144	0.106	2.729	0.113	2.025	0.281	1.798	0.302	1.750	0.279	1.681	0.291	1.966	0.285	1.865	0.304	1.827	0.301	1.812	0.297
798	12.531	3.142	0.105	2.727	0.113	2.025	0.280	1.801	0.300	1.757	0.272	1.687	0.283	1.967	0.285	1.869	0.304	1.829	0.300	1.815	0.296
796	12.563	3.140	0.105	2.725	0.114	2.025	0.280	1.805	0.297	1.762	0.264	1.693	0.274	1.967	0.284	1.872	0.303	1.830	0.300	1.818	0.294
794	12.594	3.138	0.105	2.723	0.114	2.026	0.279	1.808	0.294	1.767	0.256	1.698	0.265	1.968	0.284	1.876	0.302	1.832	0.299	1.821	0.292
792	12.626	3.137	0.105	2.721	0.114	2.026	0.279	1.811	0.291	1.771	0.247	1.702	0.256	1.969	0.284	1.880	0.301	1.834	0.297	1.824	0.290
790	12.658	3.135	0.105	2.720	0.115	2.027	0.278	1.814	0.288	1.774	0.239	1.706	0.247	1.970	0.283	1.883	0.299	1.836	0.296	1.827	0.288
788	12.690	3.134	0.105	2.718	0.115	2.027	0.277	1.816	0.284	1.776	0.230	1.708	0.237	1.970	0.282	1.886	0.296	1.838	0.294	1.830	0.285

Wavenumber (cm^{-1})	Wavelength (μm)	n10	k10	n20	k20	n30	k30	n40	k40	n80	k80	n120	k120	n31	k31	n32	k32	n33	k33	n34	k34
786	12.723	3.132	0.105	2.716	0.116	2.027	0.276	1.818	0.280	1.778	0.221	1.709	0.227	1.971	0.281	1.890	0.293	1.839	0.293	1.832	0.282
784	12.755	3.131	0.105	2.715	0.116	2.028	0.275	1.820	0.276	1.778	0.212	1.710	0.217	1.972	0.280	1.893	0.290	1.841	0.291	1.835	0.278
782	12.788	3.129	0.105	2.713	0.117	2.028	0.274	1.822	0.272	1.778	0.203	1.709	0.208	1.973	0.279	1.895	0.287	1.842	0.288	1.837	0.275
780	12.821	3.128	0.105	2.712	0.117	2.028	0.273	1.824	0.267	1.777	0.194	1.708	0.198	1.973	0.278	1.898	0.283	1.844	0.286	1.839	0.271
778	12.853	3.126	0.105	2.710	0.118	2.028	0.272	1.825	0.262	1.775	0.185	1.706	0.189	1.974	0.277	1.900	0.279	1.845	0.284	1.840	0.267
776	12.887	3.125	0.105	2.709	0.118	2.029	0.271	1.825	0.258	1.773	0.177	1.703	0.180	1.974	0.275	1.902	0.275	1.846	0.281	1.842	0.263
774	12.920	3.123	0.105	2.708	0.119	2.029	0.270	1.826	0.253	1.770	0.169	1.700	0.172	1.975	0.274	1.903	0.270	1.848	0.278	1.843	0.259
772	12.953	3.122	0.106	2.706	0.120	2.029	0.268	1.826	0.248	1.767	0.161	1.696	0.163	1.975	0.272	1.904	0.265	1.849	0.275	1.844	0.254
770	12.987	3.121	0.106	2.705	0.120	2.029	0.267	1.826	0.243	1.763	0.154	1.691	0.156	1.976	0.271	1.905	0.261	1.849	0.272	1.845	0.250
768	13.021	3.120	0.106	2.704	0.121	2.029	0.266	1.825	0.238	1.759	0.147	1.686	0.149	1.976	0.269	1.905	0.256	1.850	0.269	1.845	0.245
766	13.055	3.118	0.106	2.703	0.121	2.030	0.264	1.825	0.233	1.754	0.141	1.681	0.142	1.977	0.267	1.905	0.251	1.851	0.266	1.845	0.241
764	13.089	3.117	0.107	2.702	0.122	2.030	0.263	1.824	0.228	1.749	0.135	1.675	0.136	1.977	0.265	1.905	0.246	1.851	0.263	1.845	0.236
762	13.123	3.116	0.107	2.700	0.122	2.030	0.261	1.822	0.223	1.744	0.130	1.669	0.131	1.977	0.264	1.905	0.241	1.851	0.259	1.845	0.231
760	13.158	3.115	0.107	2.699	0.123	2.030	0.260	1.821	0.218	1.738	0.125	1.663	0.126	1.977	0.262	1.904	0.236	1.851	0.256	1.844	0.227
758	13.193	3.114	0.108	2.698	0.124	2.029	0.258	1.819	0.213	1.733	0.120	1.657	0.121	1.977	0.260	1.902	0.231	1.851	0.252	1.843	0.222
756	13.228	3.113	0.108	2.697	0.124	2.029	0.256	1.816	0.209	1.727	0.116	1.650	0.117	1.977	0.258	1.901	0.226	1.851	0.249	1.842	0.217
754	13.263	3.112	0.109	2.696	0.125	2.029	0.255	1.814	0.204	1.721	0.112	1.644	0.114	1.977	0.255	1.899	0.222	1.850	0.245	1.840	0.213
752	13.298	3.111	0.109	2.695	0.125	2.029	0.253	1.811	0.200	1.715	0.109	1.637	0.110	1.977	0.253	1.896	0.217	1.850	0.242	1.839	0.208
750	13.333	3.110	0.109	2.694	0.126	2.029	0.251	1.808	0.196	1.709	0.106	1.631	0.108	1.976	0.251	1.894	0.213	1.849	0.238	1.837	0.204
748	13.369	3.109	0.110	2.693	0.127	2.028	0.250	1.805	0.192	1.704	0.103	1.625	0.106	1.976	0.249	1.891	0.209	1.848	0.235	1.835	0.200
746	13.405	3.108	0.110	2.692	0.127	2.028	0.248	1.802	0.188	1.698	0.101	1.618	0.104	1.975	0.247	1.888	0.205	1.847	0.231	1.833	0.195
744	13.441	3.107	0.111	2.691	0.128	2.027	0.246	1.799	0.184	1.692	0.099	1.612	0.102	1.975	0.245	1.885	0.201	1.846	0.228	1.830	0.191
742	13.477	3.106	0.111	2.691	0.129	2.027	0.245	1.795	0.181	1.686	0.097	1.606	0.101	1.974	0.243	1.882	0.197	1.844	0.225	1.827	0.187
740	13.514	3.105	0.112	2.690	0.129	2.026	0.243	1.791	0.177	1.681	0.096	1.600	0.100	1.973	0.240	1.879	0.194	1.843	0.221	1.825	0.184
738	13.550	3.104	0.112	2.689	0.130	2.026	0.241	1.788	0.174	1.675	0.095	1.595	0.099	1.973	0.238	1.875	0.191	1.841	0.218	1.822	0.180
736	13.587	3.103	0.113	2.688	0.130	2.025	0.239	1.784	0.171	1.670	0.094	1.589	0.099	1.972	0.236	1.871	0.188	1.839	0.215	1.818	0.177
734	13.624	3.103	0.113	2.687	0.131	2.024	0.238	1.780	0.169	1.665	0.093	1.584	0.099	1.971	0.234	1.868	0.185	1.837	0.212	1.815	0.173
732	13.661	3.102	0.114	2.686	0.132	2.023	0.236	1.776	0.166	1.660	0.092	1.579	0.099	1.970	0.232	1.864	0.183	1.835	0.208	1.812	0.170
730	13.699	3.101	0.114	2.686	0.132	2.023	0.234	1.771	0.164	1.655	0.092	1.574	0.099	1.968	0.229	1.860	0.181	1.833	0.206	1.808	0.167
728	13.736	3.100	0.115	2.685	0.133	2.022	0.232	1.767	0.162	1.650	0.092	1.569	0.099	1.967	0.227	1.856	0.179	1.831	0.203	1.805	0.164
726	13.774	3.100	0.115	2.684	0.134	2.021	0.231	1.763	0.160	1.645	0.092	1.564	0.099	1.966	0.225	1.852	0.177	1.828	0.200	1.801	0.162
724	13.812	3.099	0.116	2.684	0.134	2.020	0.229	1.759	0.159	1.641	0.091	1.559	0.100	1.964	0.223	1.848	0.175	1.826	0.197	1.798	0.159

Wavenumber (cm^{-1})	Wavelength (μm)	n10	k10	n20	k20	n30	k30	n40	k40	n80	k80	n120	k120	n31	k31	n32	k32	n33	k33	n34	k34
722	13.850	3.098	0.116	2.683	0.135	2.019	0.227	1.755	0.157	1.636	0.092	1.555	0.100	1.963	0.221	1.844	0.174	1.823	0.195	1.794	0.157
720	13.889	3.098	0.117	2.682	0.135	2.017	0.226	1.750	0.156	1.632	0.092	1.551	0.101	1.961	0.219	1.840	0.173	1.820	0.192	1.790	0.155
718	13.928	3.097	0.118	2.681	0.136	2.016	0.224	1.746	0.155	1.627	0.092	1.546	0.101	1.960	0.217	1.837	0.172	1.818	0.190	1.786	0.153
716	13.966	3.096	0.118	2.681	0.137	2.015	0.222	1.742	0.154	1.623	0.092	1.542	0.102	1.958	0.215	1.833	0.171	1.815	0.187	1.782	0.151
714	14.006	3.096	0.119	2.680	0.137	2.014	0.221	1.738	0.153	1.619	0.093	1.538	0.103	1.956	0.214	1.829	0.170	1.812	0.185	1.778	0.149
712	14.045	3.095	0.119	2.680	0.138	2.013	0.219	1.734	0.152	1.615	0.093	1.534	0.104	1.955	0.212	1.825	0.170	1.809	0.183	1.775	0.148
710	14.085	3.095	0.120	2.679	0.139	2.011	0.218	1.730	0.152	1.611	0.093	1.531	0.104	1.953	0.210	1.822	0.169	1.806	0.181	1.771	0.147
708	14.124	3.094	0.120	2.678	0.139	2.010	0.216	1.726	0.151	1.607	0.094	1.527	0.105	1.951	0.208	1.818	0.169	1.803	0.180	1.767	0.145
706	14.164	3.094	0.121	2.678	0.140	2.008	0.215	1.722	0.151	1.603	0.094	1.523	0.106	1.949	0.207	1.815	0.169	1.800	0.178	1.763	0.144
704	14.205	3.093	0.122	2.677	0.141	2.007	0.213	1.718	0.151	1.600	0.095	1.520	0.107	1.947	0.205	1.811	0.169	1.797	0.176	1.759	0.143
702	14.245	3.093	0.122	2.677	0.141	2.005	0.212	1.714	0.151	1.596	0.096	1.516	0.108	1.945	0.204	1.808	0.169	1.794	0.175	1.755	0.143
700	14.286	3.092	0.123	2.676	0.142	2.004	0.210	1.710	0.151	1.592	0.096	1.513	0.108	1.943	0.202	1.805	0.169	1.790	0.174	1.752	0.142
698	14.327	3.092	0.123	2.676	0.142	2.002	0.209	1.707	0.151	1.589	0.097	1.510	0.109	1.941	0.201	1.801	0.169	1.787	0.172	1.748	0.141
696	14.368	3.091	0.124	2.675	0.143	2.001	0.208	1.703	0.151	1.585	0.098	1.506	0.110	1.938	0.199	1.798	0.170	1.784	0.171	1.744	0.141
694	14.409	3.091	0.124	2.675	0.144	1.999	0.207	1.700	0.152	1.582	0.098	1.503	0.111	1.936	0.198	1.795	0.170	1.781	0.170	1.741	0.141
692	14.451	3.090	0.125	2.674	0.144	1.997	0.205	1.696	0.152	1.578	0.099	1.500	0.112	1.934	0.197	1.792	0.171	1.778	0.169	1.737	0.140
690	14.493	3.090	0.126	2.674	0.145	1.996	0.204	1.693	0.153	1.575	0.100	1.497	0.113	1.932	0.196	1.790	0.171	1.775	0.168	1.734	0.140
688	14.535	3.089	0.126	2.673	0.146	1.994	0.203	1.689	0.153	1.572	0.101	1.493	0.114	1.929	0.195	1.787	0.172	1.771	0.168	1.730	0.140
686	14.577	3.089	0.127	2.673	0.146	1.992	0.202	1.686	0.154	1.568	0.101	1.490	0.115	1.927	0.194	1.784	0.172	1.768	0.167	1.727	0.140
684	14.620	3.089	0.127	2.673	0.147	1.990	0.201	1.683	0.155	1.565	0.102	1.487	0.116	1.925	0.193	1.781	0.173	1.765	0.167	1.723	0.140
682	14.663	3.088	0.128	2.672	0.148	1.988	0.200	1.680	0.155	1.562	0.103	1.484	0.116	1.922	0.192	1.779	0.174	1.762	0.166	1.720	0.141
680	14.706	3.088	0.129	2.672	0.148	1.986	0.199	1.677	0.156	1.559	0.104	1.481	0.117	1.920	0.191	1.776	0.174	1.759	0.166	1.717	0.141
678	14.749	3.088	0.129	2.671	0.149	1.985	0.198	1.674	0.157	1.555	0.105	1.478	0.118	1.917	0.190	1.774	0.175	1.756	0.166	1.714	0.141
676	14.793	3.087	0.130	2.671	0.150	1.983	0.197	1.671	0.158	1.552	0.106	1.475	0.119	1.915	0.190	1.771	0.176	1.753	0.165	1.710	0.142
674	14.837	3.087	0.130	2.671	0.150	1.981	0.196	1.668	0.159	1.549	0.107	1.472	0.120	1.913	0.189	1.769	0.177	1.750	0.165	1.707	0.142
672	14.881	3.087	0.131	2.670	0.151	1.979	0.195	1.665	0.160	1.546	0.107	1.469	0.121	1.910	0.188	1.767	0.178	1.747	0.165	1.704	0.143
670	14.925	3.086	0.132	2.670	0.151	1.977	0.195	1.662	0.161	1.543	0.108	1.466	0.122	1.908	0.188	1.765	0.178	1.744	0.165	1.701	0.143
668	14.970	3.086	0.132	2.669	0.152	1.975	0.194	1.659	0.161	1.540	0.109	1.463	0.123	1.905	0.187	1.762	0.179	1.741	0.165	1.698	0.144
666	15.015	3.086	0.133	2.669	0.153	1.973	0.193	1.657	0.162	1.537	0.110	1.460	0.124	1.903	0.187	1.760	0.180	1.738	0.165	1.696	0.144
664	15.060	3.085	0.134	2.669	0.153	1.971	0.193	1.654	0.163	1.533	0.111	1.457	0.125	1.900	0.187	1.758	0.181	1.736	0.166	1.693	0.145
662	15.106	3.085	0.134	2.668	0.154	1.969	0.192	1.651	0.164	1.530	0.112	1.454	0.126	1.898	0.186	1.756	0.182	1.733	0.166	1.690	0.146
660	15.152	3.085	0.135	2.668	0.155	1.967	0.192	1.649	0.166	1.527	0.113	1.451	0.127	1.895	0.186	1.754	0.183	1.730	0.166	1.687	0.146

Wavenumber (cm^{-1})	Wavelength (μm)	n10	k10	n20	k20	n30	k30	n40	k40	n80	k80	n120	k120	n31	k31	n32	k32	n33	k33	n34	k34
658	15.198	3.085	0.135	2.668	0.155	1.965	0.191	1.646	0.167	1.524	0.114	1.449	0.128	1.893	0.186	1.752	0.184	1.727	0.166	1.685	0.147
656	15.244	3.084	0.136	2.667	0.156	1.963	0.191	1.643	0.168	1.521	0.115	1.446	0.129	1.890	0.186	1.750	0.185	1.725	0.167	1.682	0.148
654	15.291	3.084	0.137	2.667	0.157	1.960	0.191	1.641	0.169	1.518	0.116	1.443	0.130	1.888	0.186	1.748	0.186	1.722	0.167	1.679	0.149
652	15.337	3.084	0.137	2.667	0.157	1.958	0.190	1.638	0.170	1.515	0.117	1.440	0.131	1.886	0.186	1.746	0.187	1.719	0.168	1.677	0.149
650	15.385	3.084	0.138	2.667	0.158	1.956	0.190	1.636	0.171	1.512	0.119	1.437	0.132	1.883	0.186	1.744	0.188	1.717	0.168	1.674	0.150
648	15.432	3.083	0.139	2.666	0.159	1.954	0.190	1.634	0.172	1.509	0.120	1.434	0.133	1.881	0.186	1.743	0.188	1.714	0.169	1.672	0.151
646	15.480	3.083	0.139	2.666	0.159	1.952	0.189	1.631	0.173	1.506	0.121	1.431	0.134	1.878	0.187	1.741	0.189	1.712	0.169	1.669	0.152
644	15.528	3.083	0.140	2.666	0.160	1.950	0.189	1.629	0.174	1.503	0.122	1.428	0.136	1.876	0.187	1.739	0.190	1.709	0.170	1.667	0.153
642	15.576	3.083	0.140	2.666	0.161	1.948	0.189	1.626	0.175	1.500	0.123	1.425	0.137	1.874	0.187	1.737	0.191	1.707	0.171	1.664	0.154
640	15.625	3.083	0.141	2.665	0.161	1.946	0.189	1.624	0.177	1.497	0.124	1.422	0.138	1.871	0.187	1.736	0.192	1.704	0.171	1.662	0.155
638	15.674	3.082	0.142	2.665	0.162	1.944	0.189	1.622	0.178	1.493	0.125	1.419	0.139	1.869	0.188	1.734	0.193	1.702	0.172	1.659	0.155
636	15.723	3.082	0.142	2.665	0.162	1.942	0.189	1.619	0.179	1.490	0.127	1.416	0.140	1.867	0.188	1.732	0.194	1.699	0.173	1.657	0.156
634	15.773	3.082	0.143	2.665	0.163	1.940	0.189	1.617	0.180	1.487	0.128	1.413	0.141	1.864	0.189	1.730	0.195	1.697	0.174	1.655	0.157
632	15.823	3.082	0.144	2.664	0.164	1.937	0.189	1.615	0.181	1.484	0.129	1.410	0.142	1.862	0.189	1.729	0.196	1.695	0.174	1.652	0.158
630	15.873	3.082	0.144	2.664	0.164	1.935	0.189	1.612	0.182	1.481	0.130	1.407	0.143	1.860	0.190	1.727	0.197	1.692	0.175	1.650	0.159
628	15.924	3.082	0.145	2.664	0.165	1.933	0.190	1.610	0.183	1.478	0.132	1.404	0.145	1.857	0.190	1.726	0.199	1.690	0.176	1.648	0.160
626	15.974	3.081	0.145	2.664	0.166	1.931	0.190	1.608	0.185	1.475	0.133	1.400	0.146	1.855	0.191	1.724	0.200	1.688	0.177	1.645	0.161
624	16.026	3.081	0.146	2.663	0.166	1.929	0.190	1.605	0.186	1.471	0.134	1.397	0.147	1.853	0.192	1.722	0.201	1.686	0.178	1.643	0.162
622	16.077	3.081	0.147	2.663	0.167	1.927	0.190	1.603	0.187	1.468	0.135	1.394	0.148	1.851	0.192	1.721	0.202	1.683	0.179	1.641	0.163
620	16.129	3.081	0.147	2.663	0.168	1.925	0.191	1.601	0.188	1.465	0.137	1.391	0.149	1.848	0.193	1.719	0.203	1.681	0.179	1.639	0.164
618	16.181	3.081	0.148	2.663	0.168	1.923	0.191	1.598	0.189	1.462	0.138	1.388	0.151	1.846	0.194	1.718	0.204	1.679	0.180	1.636	0.165
616	16.234	3.081	0.149	2.663	0.169	1.921	0.191	1.596	0.191	1.458	0.140	1.384	0.152	1.844	0.194	1.716	0.205	1.677	0.181	1.634	0.166
614	16.287	3.081	0.149	2.662	0.170	1.918	0.192	1.593	0.192	1.455	0.141	1.381	0.153	1.842	0.195	1.715	0.206	1.674	0.182	1.632	0.167
612	16.340	3.081	0.150	2.662	0.170	1.916	0.192	1.591	0.193	1.451	0.142	1.378	0.155	1.840	0.196	1.713	0.207	1.672	0.183	1.630	0.168
610	16.393	3.080	0.151	2.662	0.171	1.914	0.192	1.589	0.194	1.448	0.144	1.374	0.156	1.838	0.197	1.712	0.208	1.670	0.184	1.627	0.169
608	16.447	3.080	0.151	2.662	0.172	1.912	0.193	1.586	0.196	1.445	0.145	1.371	0.157	1.835	0.198	1.710	0.209	1.668	0.185	1.625	0.170
606	16.502	3.080	0.152	2.662	0.172	1.910	0.194	1.584	0.197	1.441	0.147	1.367	0.159	1.833	0.199	1.708	0.210	1.666	0.186	1.623	0.171
604	16.556	3.080	0.153	2.662	0.173	1.908	0.194	1.581	0.198	1.438	0.148	1.364	0.160	1.831	0.200	1.707	0.211	1.664	0.187	1.620	0.173
602	16.611	3.080	0.153	2.661	0.174	1.906	0.195	1.579	0.199	1.434	0.150	1.360	0.161	1.829	0.201	1.705	0.213	1.661	0.188	1.618	0.174
600	16.667	3.080	0.154	2.661	0.174	1.904	0.195	1.576	0.201	1.430	0.152	1.356	0.163	1.827	0.202	1.704	0.214	1.659	0.189	1.616	0.175
598	16.722	3.080	0.154	2.661	0.175	1.902	0.196	1.573	0.202	1.427	0.153	1.352	0.164	1.825	0.203	1.702	0.215	1.657	0.190	1.613	0.176
596	16.779	3.080	0.155	2.661	0.176	1.899	0.197	1.571	0.203	1.423	0.155	1.349	0.166	1.823	0.204	1.701	0.216	1.655	0.191	1.611	0.177

Wavenumber (cm^{-1})	Wavelength (µm)	n10	k10	n20	k20	n30	k30	n40	k40	n80	k80	n120	k120	n31	k31	n32	k32	n33	k33	n34	k34
594	16.835	3.080	0.156	2.661	0.176	1.897	0.197	1.568	0.204	1.419	0.157	1.345	0.167	1.821	0.205	1.699	0.217	1.652	0.192	1.609	0.178
592	16.892	3.080	0.156	2.661	0.177	1.895	0.198	1.565	0.206	1.415	0.158	1.341	0.169	1.819	0.206	1.698	0.218	1.650	0.194	1.606	0.179
590	16.949	3.080	0.157	2.660	0.178	1.893	0.199	1.562	0.207	1.411	0.160	1.337	0.170	1.817	0.207	1.696	0.219	1.648	0.195	1.604	0.180
588	17.007	3.080	0.158	2.660	0.178	1.891	0.199	1.559	0.208	1.407	0.162	1.333	0.172	1.815	0.208	1.695	0.221	1.646	0.196	1.601	0.182
586	17.065	3.080	0.158	2.660	0.179	1.889	0.200	1.557	0.210	1.403	0.164	1.328	0.173	1.812	0.209	1.693	0.222	1.643	0.197	1.599	0.183
584	17.123	3.080	0.159	2.660	0.180	1.886	0.201	1.554	0.211	1.399	0.165	1.324	0.175	1.810	0.211	1.692	0.223	1.641	0.198	1.596	0.184
582	17.182	3.079	0.160	2.660	0.180	1.884	0.202	1.550	0.212	1.395	0.167	1.320	0.177	1.808	0.212	1.690	0.224	1.639	0.199	1.594	0.185
580	17.241	3.079	0.160	2.660	0.181	1.882	0.203	1.547	0.214	1.390	0.169	1.315	0.178	1.806	0.213	1.689	0.225	1.636	0.200	1.591	0.186
578	17.301	3.079	0.161	2.659	0.182	1.880	0.204	1.544	0.215	1.386	0.171	1.310	0.180	1.804	0.214	1.687	0.227	1.634	0.202	1.588	0.188
576	17.361	3.079	0.162	2.659	0.182	1.877	0.204	1.541	0.217	1.381	0.173	1.306	0.182	1.802	0.215	1.685	0.228	1.631	0.203	1.585	0.189
574	17.422	3.079	0.162	2.659	0.183	1.875	0.205	1.537	0.218	1.376	0.175	1.301	0.184	1.800	0.217	1.684	0.229	1.629	0.204	1.583	0.190
572	17.483	3.079	0.163	2.659	0.184	1.873	0.206	1.534	0.220	1.372	0.178	1.296	0.185	1.798	0.218	1.682	0.230	1.626	0.206	1.580	0.192
570	17.544	3.079	0.164	2.659	0.184	1.870	0.207	1.530	0.221	1.367	0.180	1.291	0.187	1.796	0.219	1.680	0.232	1.624	0.207	1.577	0.193
568	17.606	3.079	0.164	2.659	0.185	1.868	0.208	1.526	0.223	1.362	0.182	1.285	0.189	1.794	0.221	1.679	0.233	1.621	0.208	1.574	0.194
566	17.668	3.079	0.165	2.659	0.186	1.866	0.209	1.522	0.224	1.356	0.184	1.280	0.191	1.791	0.222	1.677	0.234	1.618	0.210	1.571	0.196
564	17.730	3.079	0.166	2.658	0.186	1.863	0.210	1.518	0.226	1.351	0.187	1.274	0.193	1.789	0.223	1.675	0.236	1.615	0.211	1.567	0.197
562	17.794	3.079	0.166	2.658	0.187	1.861	0.211	1.514	0.228	1.346	0.189	1.268	0.195	1.787	0.225	1.674	0.237	1.612	0.213	1.564	0.199
560	17.857	3.079	0.167	2.658	0.188	1.858	0.212	1.510	0.229	1.340	0.192	1.262	0.197	1.785	0.226	1.672	0.238	1.609	0.214	1.561	0.200
558	17.921	3.079	0.168	2.658	0.188	1.855	0.214	1.505	0.231	1.334	0.194	1.256	0.200	1.782	0.228	1.670	0.240	1.606	0.216	1.557	0.202
556	17.986	3.079	0.168	2.658	0.189	1.853	0.215	1.500	0.233	1.328	0.197	1.250	0.202	1.780	0.229	1.668	0.241	1.603	0.218	1.554	0.204
554	18.051	3.079	0.169	2.657	0.190	1.850	0.216	1.495	0.235	1.322	0.200	1.243	0.204	1.778	0.231	1.666	0.242	1.600	0.219	1.550	0.206
552	18.116	3.079	0.170	2.657	0.190	1.847	0.217	1.490	0.237	1.315	0.203	1.236	0.207	1.775	0.232	1.664	0.244	1.597	0.221	1.546	0.208
550	18.182	3.079	0.171	2.657	0.191	1.844	0.218	1.485	0.239	1.308	0.206	1.229	0.209	1.773	0.234	1.662	0.245	1.593	0.223	1.542	0.210
548	18.248	3.079	0.171	2.657	0.192	1.841	0.220	1.479	0.241	1.301	0.209	1.221	0.212	1.770	0.236	1.660	0.247	1.590	0.225	1.538	0.212
546	18.315	3.079	0.172	2.657	0.192	1.838	0.221	1.473	0.243	1.294	0.212	1.213	0.215	1.768	0.237	1.658	0.248	1.586	0.228	1.534	0.214
544	18.382	3.079	0.173	2.656	0.193	1.835	0.222	1.467	0.246	1.287	0.216	1.205	0.217	1.765	0.239	1.656	0.250	1.582	0.230	1.529	0.216
542	18.450	3.079	0.173	2.656	0.194	1.831	0.224	1.460	0.249	1.279	0.219	1.196	0.220	1.762	0.241	1.653	0.252	1.579	0.232	1.525	0.219
540	18.519	3.079	0.174	2.656	0.195	1.828	0.225	1.453	0.251	1.270	0.223	1.187	0.224	1.760	0.243	1.651	0.253	1.575	0.235	1.520	0.222
538	18.587	3.079	0.175	2.656	0.195	1.824	0.227	1.446	0.254	1.262	0.227	1.178	0.227	1.757	0.244	1.648	0.255	1.570	0.238	1.515	0.224
536	18.657	3.079	0.175	2.655	0.196	1.821	0.229	1.438	0.258	1.252	0.232	1.168	0.230	1.754	0.246	1.646	0.257	1.566	0.241	1.510	0.228
534	18.727	3.079	0.176	2.655	0.197	1.817	0.230	1.430	0.261	1.243	0.236	1.157	0.234	1.751	0.248	1.643	0.259	1.562	0.244	1.505	0.231
532	18.797	3.079	0.177	2.655	0.197	1.812	0.232	1.421	0.265	1.233	0.241	1.146	0.238	1.748	0.251	1.640	0.261	1.557	0.248	1.499	0.235

Wavenumber (cm^{-1})	Wavelength (μm)	n10	k10	n20	k20	n30	k30	n40	k40	n80	k80	n120	k120	n31	k31	n32	k32	n33	k33	n34	k34
530	18.868	3.079	0.178	2.655	0.198	1.808	0.234	1.412	0.269	1.222	0.246	1.134	0.242	1.744	0.253	1.638	0.263	1.553	0.251	1.493	0.239
528	18.939	3.079	0.178	2.654	0.199	1.804	0.237	1.402	0.274	1.211	0.252	1.122	0.246	1.741	0.255	1.635	0.265	1.548	0.255	1.488	0.243
526	19.011	3.079	0.179	2.654	0.200	1.799	0.239	1.392	0.279	1.198	0.258	1.108	0.251	1.738	0.258	1.632	0.268	1.544	0.260	1.482	0.248
524	19.084	3.079	0.180	2.654	0.200	1.794	0.242	1.381	0.285	1.186	0.265	1.094	0.257	1.734	0.260	1.628	0.270	1.539	0.265	1.476	0.253
522	19.157	3.079	0.180	2.653	0.201	1.789	0.244	1.369	0.292	1.172	0.272	1.078	0.262	1.730	0.263	1.625	0.273	1.534	0.270	1.469	0.259
520	19.231	3.079	0.181	2.653	0.202	1.784	0.247	1.357	0.299	1.158	0.280	1.062	0.269	1.727	0.266	1.622	0.276	1.529	0.275	1.463	0.265
518	19.305	3.079	0.182	2.652	0.202	1.778	0.251	1.344	0.308	1.142	0.289	1.044	0.276	1.723	0.269	1.618	0.279	1.524	0.281	1.457	0.272
516	19.380	3.079	0.183	2.652	0.203	1.772	0.255	1.330	0.318	1.126	0.300	1.025	0.285	1.719	0.272	1.614	0.282	1.519	0.287	1.450	0.279
514	19.455	3.079	0.183	2.652	0.204	1.766	0.259	1.316	0.329	1.108	0.311	1.004	0.295	1.715	0.276	1.611	0.285	1.514	0.294	1.444	0.287
512	19.531	3.079	0.184	2.651	0.205	1.760	0.263	1.302	0.341	1.090	0.325	0.981	0.306	1.710	0.280	1.607	0.289	1.510	0.302	1.438	0.295
510	19.608	3.079	0.185	2.651	0.205	1.754	0.269	1.287	0.355	1.070	0.340	0.957	0.319	1.706	0.284	1.603	0.293	1.505	0.309	1.431	0.304
508	19.685	3.078	0.186	2.650	0.206	1.747	0.274	1.271	0.372	1.049	0.358	0.931	0.335	1.702	0.288	1.599	0.298	1.501	0.318	1.425	0.314
506	19.763	3.078	0.186	2.650	0.207	1.740	0.280	1.256	0.390	1.028	0.378	0.903	0.354	1.697	0.293	1.595	0.303	1.496	0.327	1.420	0.325
504	19.841	3.078	0.187	2.649	0.208	1.733	0.287	1.240	0.410	1.006	0.402	0.873	0.377	1.693	0.298	1.591	0.308	1.493	0.336	1.414	0.336
502	19.920	3.078	0.188	2.648	0.209	1.726	0.295	1.225	0.433	0.984	0.430	0.842	0.405	1.688	0.303	1.587	0.314	1.489	0.346	1.409	0.348
500	20.000	3.078	0.189	2.648	0.209	1.719	0.303	1.211	0.459	0.962	0.462	0.811	0.439	1.683	0.309	1.583	0.320	1.486	0.357	1.404	0.361
498	20.080	3.078	0.190	2.647	0.210	1.713	0.313	1.198	0.488	0.941	0.499	0.780	0.478	1.679	0.316	1.579	0.326	1.483	0.368	1.400	0.375
496	20.161	3.078	0.191	2.646	0.211	1.706	0.323	1.187	0.519	0.923	0.540	0.751	0.525	1.674	0.322	1.575	0.333	1.481	0.380	1.397	0.389
494	20.243	3.078	0.191	2.645	0.212	1.699	0.334	1.177	0.553	0.907	0.586	0.726	0.579	1.670	0.330	1.571	0.341	1.480	0.392	1.394	0.404
492	20.325	3.077	0.192	2.644	0.213	1.693	0.346	1.171	0.589	0.896	0.637	0.706	0.639	1.665	0.338	1.568	0.349	1.479	0.404	1.393	0.420
490	20.408	3.077	0.193	2.643	0.213	1.687	0.359	1.167	0.628	0.890	0.691	0.693	0.704	1.661	0.346	1.564	0.358	1.479	0.417	1.392	0.436
488	20.492	3.077	0.194	2.642	0.214	1.682	0.373	1.167	0.668	0.891	0.748	0.688	0.773	1.657	0.355	1.562	0.367	1.480	0.431	1.392	0.453
486	20.576	3.077	0.195	2.641	0.215	1.678	0.388	1.170	0.710	0.897	0.807	0.691	0.844	1.653	0.364	1.559	0.377	1.481	0.444	1.393	0.470
484	20.661	3.077	0.196	2.640	0.216	1.675	0.404	1.178	0.753	0.911	0.867	0.702	0.916	1.650	0.374	1.558	0.387	1.484	0.458	1.396	0.488
482	20.747	3.076	0.197	2.639	0.217	1.672	0.421	1.190	0.796	0.931	0.926	0.721	0.987	1.647	0.385	1.556	0.398	1.487	0.472	1.400	0.505
480	20.833	3.076	0.198	2.637	0.218	1.671	0.439	1.206	0.838	0.958	0.984	0.748	1.057	1.644	0.396	1.556	0.409	1.491	0.486	1.405	0.523
478	20.921	3.076	0.199	2.636	0.219	1.671	0.457	1.226	0.880	0.991	1.040	0.781	1.124	1.642	0.408	1.556	0.420	1.497	0.500	1.411	0.541
476	21.008	3.076	0.200	2.634	0.220	1.672	0.477	1.250	0.921	1.030	1.093	0.821	1.189	1.641	0.420	1.556	0.432	1.503	0.514	1.419	0.559
474	21.097	3.076	0.202	2.632	0.221	1.675	0.496	1.277	0.960	1.074	1.142	0.868	1.250	1.641	0.433	1.558	0.444	1.511	0.527	1.428	0.576
472	21.186	3.075	0.203	2.630	0.222	1.680	0.516	1.309	0.997	1.123	1.188	0.920	1.308	1.641	0.446	1.561	0.457	1.519	0.541	1.438	0.593
470	21.277	3.075	0.204	2.628	0.223	1.686	0.536	1.343	1.031	1.177	1.228	0.977	1.360	1.642	0.460	1.564	0.469	1.528	0.553	1.450	0.609
468	21.368	3.075	0.205	2.625	0.224	1.695	0.556	1.381	1.063	1.235	1.264	1.040	1.408	1.644	0.473	1.568	0.481	1.539	0.566	1.462	0.625

Wavenumber (cm^{-1})	Wavelength (μm)	n10	k10	n20	k20	n30	k30	n40	k40	n80	k80	n120	k120	n31	k31	n32	k32	n33	k33	n34	k34
466	21.459	3.075	0.207	2.623	0.225	1.704	0.576	1.421	1.092	1.296	1.295	1.107	1.451	1.647	0.487	1.574	0.494	1.550	0.577	1.476	0.640
464	21.552	3.074	0.208	2.619	0.227	1.716	0.595	1.463	1.117	1.360	1.319	1.178	1.487	1.650	0.502	1.580	0.506	1.562	0.588	1.491	0.654
462	21.645	3.074	0.210	2.616	0.228	1.730	0.613	1.508	1.140	1.426	1.338	1.252	1.517	1.655	0.516	1.588	0.518	1.575	0.599	1.507	0.667
460	21.739	3.074	0.211	2.612	0.230	1.745	0.631	1.554	1.159	1.493	1.351	1.329	1.541	1.661	0.530	1.596	0.529	1.588	0.608	1.524	0.680
458	21.834	3.074	0.213	2.607	0.232	1.762	0.647	1.602	1.174	1.561	1.357	1.407	1.558	1.668	0.544	1.605	0.540	1.602	0.617	1.542	0.690
456	21.930	3.074	0.214	2.602	0.235	1.780	0.662	1.650	1.186	1.630	1.357	1.487	1.568	1.676	0.557	1.615	0.551	1.617	0.624	1.561	0.700
454	22.026	3.074	0.216	2.596	0.238	1.799	0.676	1.700	1.194	1.697	1.351	1.567	1.570	1.686	0.571	1.626	0.560	1.632	0.631	1.580	0.709
452	22.124	3.074	0.218	2.590	0.243	1.820	0.688	1.749	1.198	1.764	1.339	1.648	1.566	1.696	0.583	1.638	0.569	1.648	0.636	1.599	0.716
450	22.222	3.074	0.220	2.583	0.248	1.841	0.699	1.799	1.198	1.828	1.321	1.727	1.554	1.707	0.595	1.650	0.577	1.663	0.641	1.619	0.722
448	22.321	3.074	0.222	2.575	0.254	1.864	0.708	1.848	1.195	1.890	1.297	1.804	1.536	1.719	0.607	1.663	0.584	1.679	0.645	1.640	0.726
446	22.422	3.074	0.224	2.567	0.263	1.887	0.715	1.897	1.189	1.949	1.268	1.879	1.511	1.732	0.617	1.676	0.591	1.696	0.647	1.660	0.730
444	22.523	3.074	0.226	2.559	0.273	1.910	0.720	1.944	1.179	2.004	1.234	1.951	1.479	1.745	0.627	1.690	0.596	1.712	0.648	1.680	0.732
442	22.624	3.075	0.228	2.551	0.286	1.934	0.723	1.991	1.165	2.055	1.196	2.019	1.442	1.760	0.636	1.704	0.600	1.728	0.649	1.701	0.732
440	22.727	3.075	0.230	2.544	0.302	1.958	0.725	2.036	1.148	2.102	1.154	2.083	1.399	1.775	0.644	1.718	0.603	1.744	0.648	1.721	0.732
438	22.831	3.076	0.232	2.539	0.320	1.982	0.724	2.079	1.129	2.143	1.109	2.142	1.351	1.790	0.650	1.733	0.605	1.760	0.647	1.741	0.730
436	22.936	3.076	0.234	2.536	0.341	2.005	0.722	2.120	1.106	2.180	1.061	2.195	1.299	1.806	0.656	1.747	0.606	1.775	0.644	1.761	0.726
434	23.041	3.077	0.236	2.536	0.364	2.028	0.718	2.159	1.081	2.211	1.011	2.243	1.243	1.822	0.661	1.761	0.605	1.790	0.640	1.780	0.722
432	23.148	3.078	0.238	2.541	0.389	2.051	0.713	2.196	1.054	2.237	0.960	2.284	1.184	1.838	0.664	1.775	0.604	1.805	0.636	1.799	0.716
430	23.256	3.078	0.240	2.550	0.414	2.072	0.705	2.230	1.024	2.258	0.909	2.320	1.123	1.855	0.666	1.789	0.602	1.819	0.630	1.817	0.710
428	23.364	3.079	0.242	2.564	0.439	2.093	0.697	2.261	0.993	2.273	0.857	2.349	1.061	1.871	0.667	1.802	0.599	1.833	0.624	1.834	0.702
426	23.474	3.081	0.244	2.583	0.462	2.113	0.686	2.290	0.960	2.283	0.806	2.372	0.998	1.888	0.667	1.815	0.595	1.846	0.617	1.851	0.694
424	23.585	3.082	0.247	2.607	0.482	2.131	0.675	2.316	0.925	2.289	0.755	2.388	0.934	1.904	0.666	1.827	0.590	1.858	0.610	1.867	0.684
422	23.697	3.083	0.249	2.635	0.498	2.149	0.662	2.339	0.890	2.289	0.707	2.399	0.872	1.920	0.664	1.839	0.584	1.870	0.601	1.882	0.674
420	23.810	3.084	0.251	2.666	0.508	2.165	0.649	2.358	0.854	2.286	0.660	2.403	0.810	1.935	0.660	1.850	0.577	1.881	0.593	1.896	0.663
418	23.923	3.086	0.252	2.698	0.513	2.179	0.634	2.375	0.817	2.279	0.615	2.403	0.751	1.950	0.656	1.860	0.570	1.891	0.583	1.910	0.651
416	24.038	3.088	0.254	2.731	0.512	2.193	0.619	2.389	0.780	2.268	0.574	2.397	0.694	1.964	0.651	1.870	0.563	1.900	0.574	1.922	0.638
414	24.155	3.089	0.256	2.763	0.505	2.205	0.603	2.401	0.744	2.255	0.535	2.387	0.640	1.978	0.645	1.878	0.554	1.909	0.563	1.933	0.626
412	24.272	3.091	0.258	2.792	0.493	2.215	0.587	2.409	0.707	2.239	0.499	2.372	0.589	1.991	0.638	1.886	0.546	1.917	0.553	1.943	0.612
410	24.390	3.093	0.259	2.817	0.477	2.224	0.570	2.415	0.671	2.221	0.466	2.354	0.541	2.004	0.630	1.893	0.537	1.924	0.542	1.952	0.599
408	24.510	3.095	0.261	2.839	0.457	2.231	0.554	2.418	0.636	2.202	0.437	2.333	0.498	2.015	0.621	1.899	0.528	1.930	0.531	1.961	0.585
406	24.631	3.097	0.262	2.856	0.435	2.238	0.537	2.419	0.602	2.181	0.411	2.310	0.458	2.026	0.612	1.904	0.518	1.935	0.521	1.968	0.571
404	24.752	3.099	0.263	2.868	0.411	2.242	0.520	2.417	0.568	2.159	0.388	2.285	0.422	2.036	0.603	1.909	0.509	1.939	0.510	1.974	0.557

Wavenumber (cm^{-1})	Wavelength (μm)	n10	k10	n20	k20	n30	k30	n40	k40	n80	k80	n120	k120	n31	k31	n32	k32	n33	k33	n34	k34
402	24.876	3.101	0.265	2.876	0.387	2.246	0.504	2.414	0.537	2.138	0.368	2.258	0.390	2.045	0.593	1.912	0.500	1.943	0.499	1.979	0.542
400	25.000	3.103	0.266	2.879	0.365	2.248	0.487	2.408	0.506	2.116	0.350	2.230	0.362	2.053	0.583	1.915	0.490	1.946	0.488	1.983	0.528
